# A Tautoleptic Approach to Chiral Hydrogen‐Bonded Supramolecular Tubular Polymers with Large Cavity

**DOI:** 10.1002/chem.201803701

**Published:** 2018-09-06

**Authors:** Algirdas Neniškis, Dovilė Račkauskaitė, Qixun Shi, Aiden J. Robertson, Andrew Marsh, Artūras Ulčinas, Ramūnas Valiokas, Steven P. Brown, Kenneth Wärnmark, Edvinas Orentas

**Affiliations:** ^1^ Department of Organic Chemistry Vilnius University Vilnius Lithuania; ^2^ Center for Analysis and Synthesis Department of Chemistry Lund University Lund Sweden; ^3^ Department of Physics and Department of Chemistry University of Warwick Coventry UK; ^4^ Department of Nanoengineering Center for Physical Sciences and Technology Vilnius Lithuania

**Keywords:** fullerenes, hydrogen-bond, molecular tubes, self-assembly, solid-state NMR

## Abstract

A new strategy towards tubular hydrogen‐bonded polymers based on the self‐assembly of isocytosine tautomers in orthogonal directions is proposed and experimentally verified, including by ^1^H fast magic‐angle spinning (MAS) solid‐state NMR. The molecular tubes obtained possess large internal diameter and tailor‐made outer functionalities rendering them potential candidates for a number of applications.

Tubular nanoscale self‐assembled systems represent versatile supramolecular constructs^1^, ^2^, ^3^ wherein the wall of the tube separates the bulk environment into physically and chemically distinct interior and exterior regions. The open‐ended topology of these aggregates allows the entry of molecular or ionic entities making them attractive for applications including transport, flow‐through catalysis, separation and detection.3a The molecular scaffold of such tubes may also act as a protective shell to isolate and stabilize otherwise very sensitive or highly aggregative molecules, such as conjugated polymeric nanowires.^4^ Coaxial inorganic shell coated tubular architectures have resulted from the use of organic molecular tubes as a growth template.^5^ Reliable and flexible access to synthetically modifiable hydrogen‐bond (H‐bond)‐mediated tubular structures will facilitate these and other applications.

Several conceptually different approaches for tubular assemblies based on H‐bonding have been reported (Figure 1 a).3a These include foldamers (I),^6^ barrel‐like assemblies (III),^7^ stacking of covalent (II)3b and non‐covalent cyclic structures (IV).^8^ The main limitation with these strategies is either complicated synthesis (I–III), fixed length of the tube (I, III) or very small cavity size (IV) (Figure 1 a). Herein, we report a new, general and very robust strategy for polymeric tubular self‐assembly based on 2D H‐bonding of exceptionally small *C*_2_‐symmetric monomers, the scaffold of which consist of only nine carbon atoms.

**Figure 1:**
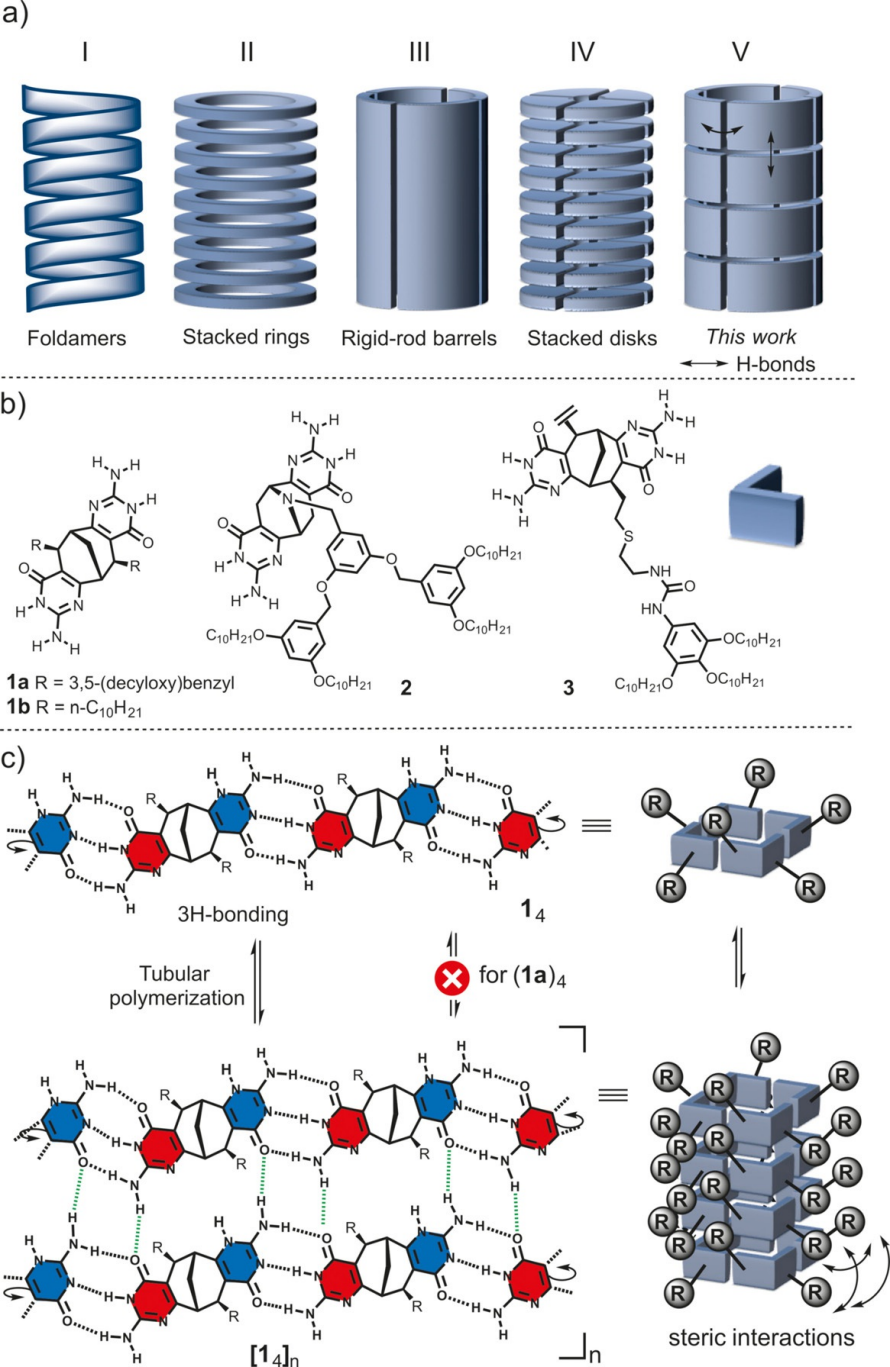
a) Schematic representation of currently available supramolecular approaches toward tubular assemblies (I–IV) and our strategy (V). b) Structure of monomers **1**–**3**. c) Two‐directional aggregation of isocytosine units in **1**–**3** leading to tubular polymers. Repulsive interaction between solubilizing groups are indicated in the schematic representation of a tubular polymer.

The structure of the monomers **1**–**3** and the proposed mechanism of tubular polymerization is outlined in Figure 1 b,c (for synthesis, see Supporting Information). In *C*_2_‐symmetric bicyclic monomers **1**–**3**, the isocytosine moiety is used as a dual purpose H‐bonding motif through tautomerization, while the bicyclo[3.3.1]nonane core allows for orthogonal preorganization of these motifs for cyclic aggregation. The use of enantiopure monomers eliminates the possible competing, non‐cyclic hetero‐chiral aggregation pathways.^9^ The anticipated three donor–donor–acceptor/acceptor–acceptor–donor H‐bonds (3H‐bonds) between two different tautomeric forms of isocytosine would form tetrameric cyclic aggregates and at the same time create new self‐complementary H‐bonding interfaces along both rims of the so‐formed cyclic units. The further stacking of the cyclic aggregates on top of each other (rim‐to‐rim) would result in the formation of a tubular polymer having an unprecedentedly large (*d*≈1.0 nm) internal diameter (Figure 1 c). In our previous studies we have shown that the enantiopure bicyclo[3.3.1]nonane scaffold fused with H‐bonding pyrrolo‐ureidopyrimidinone units is also capable of forming supramolecular tubular polymers, using one single tautomeric form of isocytosine and additional urea moieties appended to isocytosine ring serving as sticky ends.3f However, the supramolecular polymerization was only observed in very non‐polar aromatic solvents, required more complicated synthesis and the possible electronic communication between the side chains was precluded due to a rather large distance between the tetrameric units. The herein proposed tautoleptic aggregation^10^ of two different complementary forms of the same heterocycle provides a means to achieve the maximum number of H‐bonds in systems with odd numbers of H‐bond acceptors and donors in a synthetically economical way.^11^ The practical application of this self‐assembly principle remains largely unexplored with only a few examples known.^10^, ^12^

Recently we have shown that monomer **1 a** in fact does not form tubular polymers according to this proposed mechanism; instead, only cyclic tetrameric aggregates or more complex discrete H‐bonded aggregates were obtained, depending on the solvent used.^10^, ^13^ We reasoned that this is due to the steric repulsion between bulky solubilizing groups in monomer **1 a** that prevents tetrameric units from approaching each other (Figure 1 c). Herein we demonstrate that, by simple manipulation of the size of the solubilizing group, its position or connectivity to the bicyclic core, an efficient tubular polymerization can be successfully realized using orthogonal 3H‐ and 2H‐bonding between two complementary tautomeric forms of an isocytosine ring, generated by symmetry‐breaking of a single *C*_2_‐symmetric monomer.

To prevent the unfavourable steric interaction between H‐bonded tetrameric units and promote polymerization, the size of the solubilizing groups was reduced, first, for monomer **1 b**, which has linear, conformationally flexible decyl chains (Figure 2 a). In a second approach, the aza‐bicyclic^14^ analogue **2** was synthesized, with one large second‐generation Fréchet dendron as solubilizing group, however, here, located at the plane of the cyclic tetramer to minimize steric repulsion. Molecular modelling of the nanotube (**2**_4_)_*n*_ indicates that these large groups can easily be accommodated within the polymer and can interact via π–π stacking to possibly increase stability (page S28). Finally, the monomer **3** having large groups connected via flexible linkers was designed. The increase of the distance between the bicyclic core and the solubilizing group is expected to provide an additional space to incorporate even relatively bulky groups located at the periphery of the tubular polymer.

**Figure 2:**
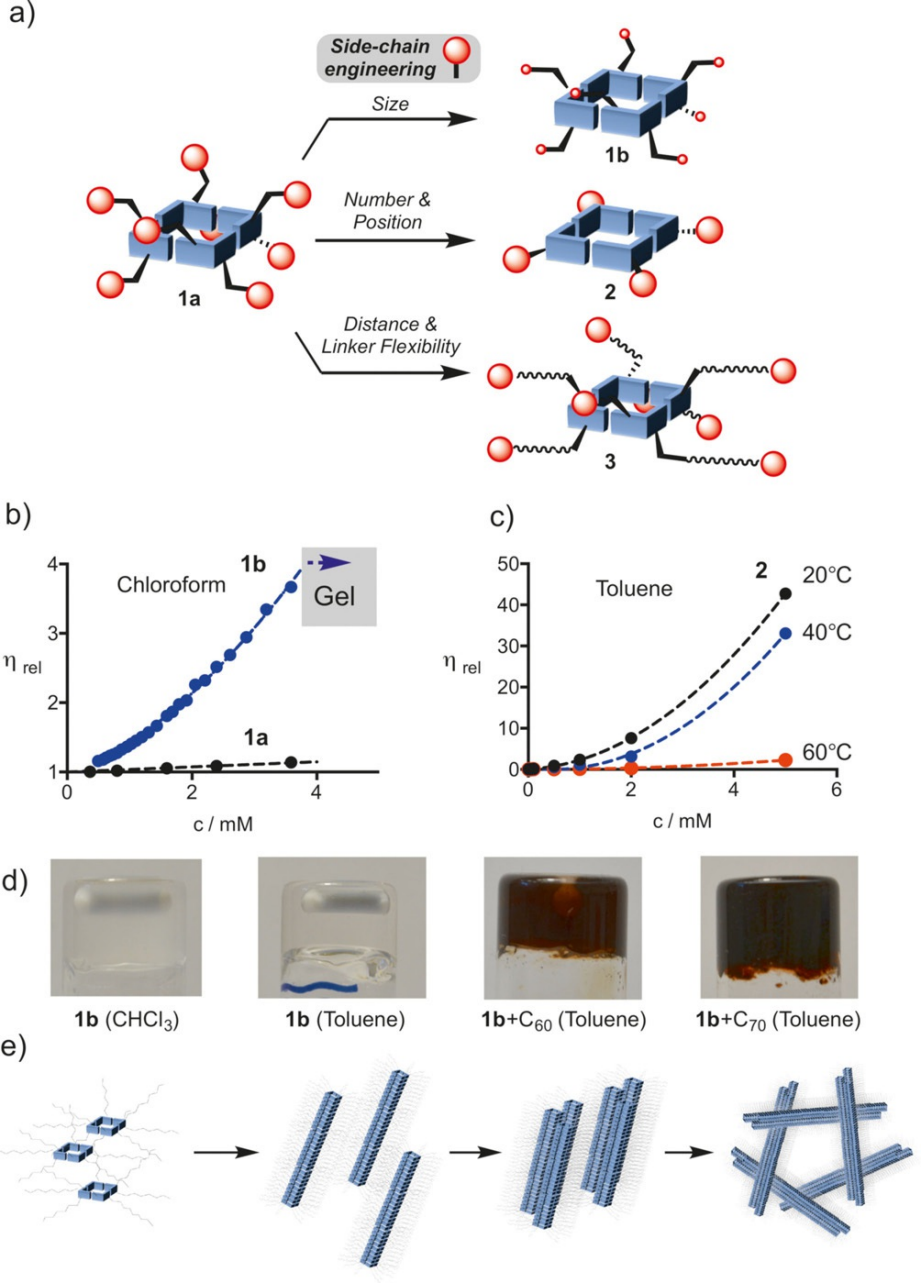
a) Structural modifications of monomer **1 a** to promote the tubular polymerization of cyclic H‐bonded units. b) Relative viscosity‐concentration plot for monomer **1 b** and reference compound **1 a** in chloroform. c) Relative viscosity‐concentration plot for monomer **2** at different temperatures in toluene. d) From left to right: gel of **1 b** in chloroform, gel of **1 b** in toluene, gel of **1 b**/C_60_ (4:1) in toluene, gel of **1 b**/C_70_ (4:1) in toluene. e) A potential gel network assembly model based on lateral aggregation of tubular polymers into bundles and their subsequent entanglement.

Indeed, the formation of polymeric aggregates from monomers **1 b**‐**3** in chloroform or toluene was immediately evident from the extremely broad and featureless ^1^H NMR spectra (see Supporting Information) as well as high solution viscosity, even at very low concentrations (Figure 2 b,c). For instance, compared to non‐polymerizing tetrameric control (**1 a**)_4_, a 4‐fold increase of relative viscosity was observed for a solution **1 b** in CHCl_3_ already at concentration below 4 mm. The tetramer (**1 a**)_4_ showed negligible increase of viscosity with concentration, typical for the solution of weakly interacting solutes. Upon increasing the concentration further, compound **1 b** formed a slightly turbid thixotropic gel (Figure 2 d). Remarkably, already a 5.0 mm solution of monomer **2** in toluene had a relative viscosity of 40, indicating the formation of long polymeric aggregates despite the bulkiness of the solubilising chains (Figure 2 c). The viscosity dropped only slightly by increasing the temperature to 40 °C, whereas at 60 °C the polymer dissociated significantly into the corresponding cyclic tetramers. After re‐cooling to ambient temperature, the viscous solution was restored. The viscosity of the solution of monomer **3** in CHCl_3_ and toluene was lower than those of **1 b** and **2**. The evaluation of the size of the aggregates in a dilute solution of **1b** (3.0 mm), **2** (0.1 mm)^15^ and **3** (3.0 mm) by dynamic light scattering (DLS) revealed distributions of hydrodynamic radius centred on 250, 113 and 50 nm, respectively (page S21). The observed trend correlates well with the viscosity data and aggregation propensity of each monomer. In addition to the turbid gel produced by **1 b** in chloroform, a transparent gel was obtained in toluene as well (Figure 2 d). The gelation mechanism mostly likely involves initial intertwining of the individual tubular polymer chains followed by the entanglement of the resulting fibers into a network (Figure 2 e). Interestingly, the interior of the tubular polymers constituting the gel network of [(**1 b)_4_]**_***n***_ in toluene can be easily filled with fullerene molecules such as C_60_ or C_70_. The encapsulation process can be followed by the colour change from magenta to brown in case of C_60_ and from brown‐red to deep‐red in case of C_70_ guests (Figures 2 d, S14). It is important to stress that racemic samples of monomers **1 b**‐**3** were completely insoluble and failed to provide viscous solutions, gels or inclusion complexes with either C_60_ or C_70_ in a variety of solvents. This observation strongly supports the preferential heterochiral aggregation of monomers in the racemate, most likely into two‐dimensional polymeric zig‐zag sheet‐like structures, via analogous 3H‐bonding between two tautomeric forms of the isocytosine (see page S20). The new modified gels obtained that are composed of ordered pea‐pod‐like tubular polymeric complexes with fullerenes are very appealing for their application in constructing soft, gel‐based electronic devices.^16^

In order to further demonstrate the proposed tubular polymerization mechanism, the dried gel of [(**1 b)_4_]**_***n***_ was analysed by high‐resolution ^1^H solid‐state magic angle spinning (MAS) NMR spectroscopy (Figure 3).^17^, ^18^ Monomer **1 b** was selected as a representative compound due to the inherent simplicity of its molecular structure and the absence of significantly overlapping cross‐peaks above 4.0 ppm. A Double Quantum–Single Quantum correlation (^1^H–^1^H DQ/SQ) experiment presented in Figure 3 b revealed several important correlations indicative of two 3H‐bonded tautomeric forms of the isocytosine (see Figure 3 a). Namely, there are two distinct proton resonances observed at 17.0 and 12.1 ppm, respectively, corresponding to the NH protons in the two tautomeric forms of the isocytosine (Figure 3 a). The observed chemical shift of proton H2 is consistent with that expected for the NH proton in the *H*[1] isocytosine tautomer, whereas the chemical shift of proton H1 is indicative of its involvement in a hydrogen bond. Moreover, the two separate resonances observed for the NH_2_ protons Ha and Hb are also consistent with the proposed aggregation mode. Unfortunately, the two different amino groups of two tautomeric forms were not discriminated, most likely due to the similar values of their respective ^1^H chemical shifts. Additional evidence for the assignment of the H2 resonance was obtained from the observed through‐space dipolar coupling between proton H2 and the bridgehead C−H proton of the bicyclic framework (Figure 3 a,b, cross‐peak CH–H2). Likewise, the dipolar couplings between protons Ha and Hb, Ha and H2 and Hb and H1 were consistent with the model of the two isocytosine tautomers connected via 3H‐bonding (Figure 3 a). A ^14^N–^1^H HMQC spectrum, presented in Figure 3 c, confirmed that all proton resonances observed above ≈5.0 ppm correspond to those protons directly bonded to nitrogen nuclei. Therefore, the involvement of the enolic form of isocytosine in H‐bonding can be ruled out. The ^13^C{^1^H} cross‐polarization (CP) MAS spectrum further supports the proposed aggregation mode (Figure 3 d). The observed splitting of the ^13^C resonances assigned to the C2 and C4 carbon atoms and the values of the chemical shifts are in line with the data, reported for the 3H‐bonded dimer of the parent isocytosine.^19^

**Figure 3:**
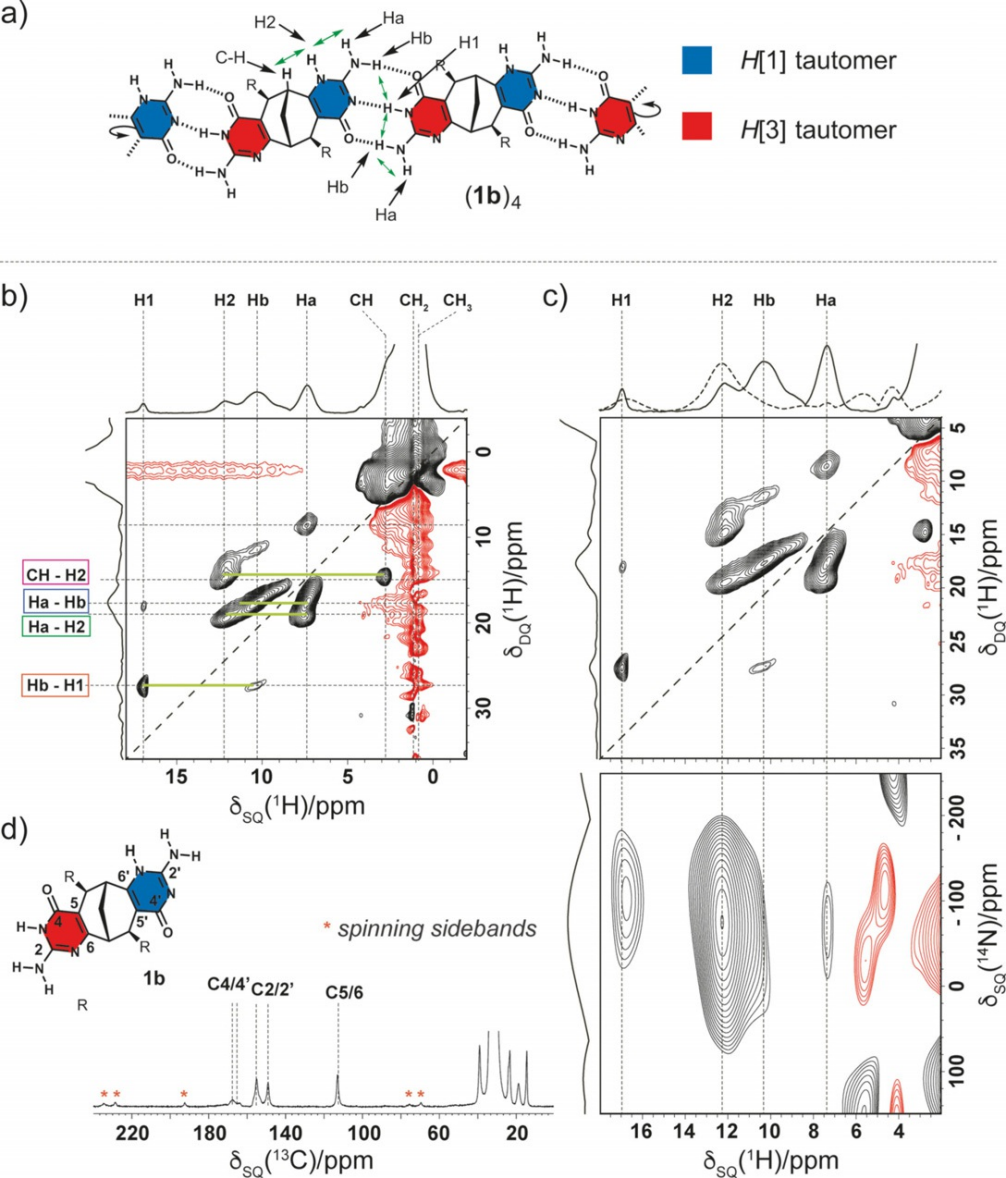
a) Schematic structure of the H‐bonded cyclic tetramer (**1 b**)_4_ with proton labels and through‐space interactions (green arrows) indicated. b) A two‐dimensional solid state ^1^H–^1^H DQ/SQ MAS (600 MHz, 60 kHz MAS) spectrum of **1 b** with assignments. Cross peaks corresponding to specific through‐space interactions are indicated with green lines. c) *Top*–zoomed‐in region of the ^1^H–^1^H DQ/SQ MAS spectrum from b). *Bottom*–A ^14^N–^1^H HMQC spectrum (600 MHz, 60 kHz MAS). A 1D HMQC‐filtered spectrum (dashed line) is overlaid with the a 1D DQ‐filtered spectrum (solid line). d) A ^13^C{^1^H} CP MAS (500 MHz, 10 kHz MAS) spectrum of **1 b** with assignments.

Finally, the formation of the tubular polymeric aggregates on a solid surface was investigated by atomic force microscopy (AFM). The chloroform solution of **1 b** was cast on freshly prepared mica surface by evaporation of the solvent with a nitrogen gas stream (Figure 4). Inspection of an AFM image (Figure 4 a) of the film formed from a solution of **1 b** of higher concentration revealed a gel‐like fibrous network structure composed of entangled bundles of tubular polymers and provided an indirect proof for the gelation mechanism outlined in Figure 2 d. On the other hand, further dilution of the stock solution allowed the direct visualization of laterally arranged individual molecular tubes (Figure 4 b). According to the cross‐section profile, two types of aggregates were present having a height of 1.0 and 0.5 nm, respectively. Based on molecular models, the dimensions of the former aggregates correspond well to intact molecular tubes, whereas smaller aggregates would most likely result from a collapse of molecular tubes due to surface attraction (Figure 4 b, bottom). Interestingly, when not entangled within the fibril network, the molecular tubes [(**1 b**)_4_]_*n*_ and the half‐sized aggregates were found to arrange themselves on the surface preferentially along the hexagonal cell unit axes of mica (Figure 4 b, top). This surface templation effect can be rationalized by charge‐dipole interactions between potassium ions on a mica surface and the heteroatoms of the isocytosine ring, in particular the oxygen of the carbonyl groups that are concentrated in the longitudinal seam of H‐bonds along the length of the polymer backbone (Figure 4 c).^20^ The very same attractive electrostatic force might be responsible for the collapse of some of the tubular polymers, especially, if the stabilizing encapsulated solvent molecules are removed from the interior of the tube during the preparation of the film.

**Figure 4:**
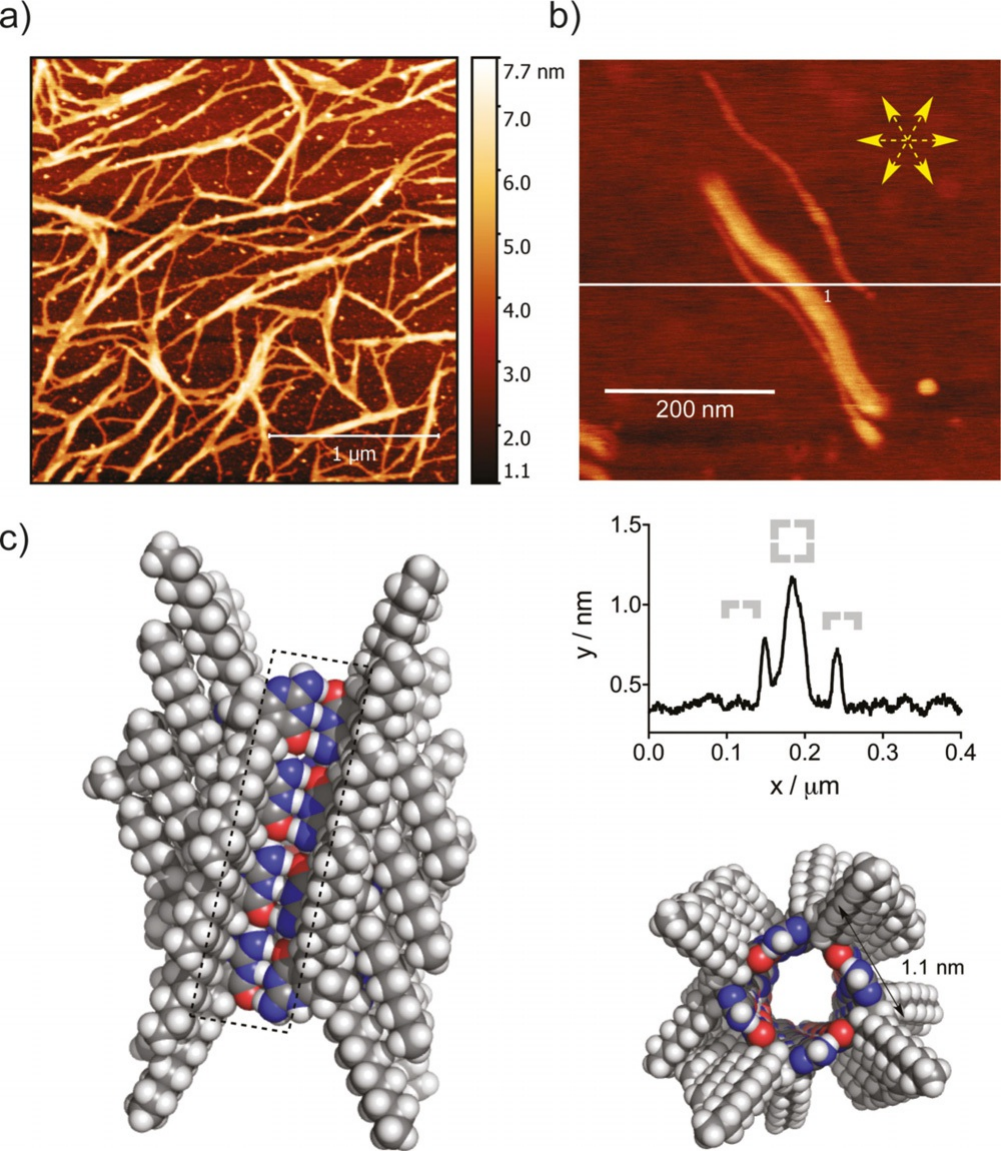
a) AFM image of the fibrous network obtained from diluted sol of **1 b** in chloroform. b) *Top*–AFM image of laterally aggregated tubular aggregates. The white line indicates the location of the cross‐section scan, and the yellow star indicates the crystallographic axes of mica; *Middle*–cross‐section profile showing intact and partially collapsed nanotubes; *Bottom*–top‐view of the molecular model of the tubular polymer (**1 b**)_*n*_. c) Side‐view of the molecular model of the tubular polymer with the polar H‐bonding interface indicated by a dashed rectangle.

In conclusion, for the first time, it has been demonstrated that small *C*_2_‐symmetric enantiopure bicyclic molecules with embedded tautomerizable isocytosine H‐bonding units can be used to obtain tubular polymeric structures with a large cavity enabling encapsulation of fullerenes. Three different strategies to synthetically modify the surface of the nanotube by structural variation of the monomer were proposed. The approach described herein not only allows access to a new family of supramolecular tubes of broad applicability, but also presents a new way to explore H‐bonding in self‐assembly by using one single H‐bonding motif to create another orthogonal H‐bonding interface upon dimerization. The use of tautoleptic aggregation, where two different tautomeric forms of the same heterocycle are forming the maximum possible number of H‐bonds, enormously simplifies the synthesis of the required ditopic monomer.

## Conflict of interest

The authors declare no conflict of interest.

## Supporting information

As a service to our authors and readers, this journal provides supporting information supplied by the authors. Such materials are peer reviewed and may be re‐organized for online delivery, but are not copy‐edited or typeset. Technical support issues arising from supporting information (other than missing files) should be addressed to the authors.

Supplementary
