# Supplementary material for: A Tautoleptic Approach to Chiral Hydrogen‐Bonded Supramolecular Tubular Polymers with Large Cavity
Source: Chemistry. 2018 Sep 6;24(53):14028–33. doi: 10.1002/chem.201803701 (PMC6391958; doi:10.1002/chem.201803701)
Supplement: Supplementary file 1 — Supplementary [file CHEM-24-14028-s001.pdf]

CHEMISTRY
A European Journal

Supporting Information

A Tautoleptic Approach to Chiral Hydrogen-Bonded Supramolecular Tubular Polymers with Large Cavity

Algirdas Neniškis+,[a] Dovilė Račkauskaitė+,[a] Qixun Shi,[b] Aiden J. Robertson,[c] Andrew Marsh,[c] Artūras Ulčinas,[d] Ramūnas Valiokas,[d] Steven P. Brown,[c] Kenneth Wärnmark,*[b] and Edvinas Orentas*[a, d]

chem_201803701_sm_miscellaneous_information.pdf

Contents

Materials and Methods S5
S1. Synthesis S6
Synthesis of 7 . . . . S7
Synthesis of 10 . . . . S8
Synthesis of 2 . . . . S9
Synthesis of 11 . . . . S10
Synthesis of 12 . . . . S11
Synthesis of 14 . . . . S12
Synthesis of 3 . . . . S14
S2. Solid State NMR S15
S2.2 Racemic vs chiral monomer S18
S3. Dynamic Light Scattering S21
S4. Viscosimetry S22
S5. Preparation of Gels S24
S6. Atomic Force Microscopy S25
S7. Host-guest chemistry S27
S8. Molecular modelling S28
S9. Copies of NMR spectra S29
S9. References S61

List of Figures

S1 Synthetic routes to target compounds. S6
S2 ^{1}H DQ/SQ MAS spectrum of enantiopure and racemic **1b** S18
S3 ^{15}N CP MAS spectra of enantiopure and racemic **1b** S19
S4 Schematic representation of the formation of insoluble corrugated sheets via 2D 3H- heterochiral and 2H-homochiral bonding between the enantiomers of **1b**. Solubilizing chains are omitted for clarity. S20
S5 Dynamic light scattering data for **1b**, **2** and **3** in toluene. S21
S6 Concentration dependence of hydrodynamic radii of $(\mathbf{2}_4)_n$ aggregates in chloroform. S21
S7 log-log plot of specific viscosity *vs* concentration for **2** in toluene S22
S8 log-log plot of specific viscosity vs concentration for **1b** in chloroform. S22
S9 Viscosity-concentration plot for **1b** in chloroform in the presence of chain stopper . S23
S10 Self-sustainable gels of C_{60}@$(1b_4)_n$ in toluene (a), C_{70}@$(1b_4)_n$ in toluene (b), $(1b_4)_n$ (c) in toluene, $(1b_4)_n$ in chloroform (d) and suspension of racemic $(1b_4)_n$ and C_{60} in toluene. S24
S11 AFM images (toluene solution) of $(1b_4)_n$ (a-b), phase-contrast AFM image of $(1b_4)_n$ (c) and $(3_4)_n$ (d). S25
S12 Histogram of oriented gradients analysis performed on AFM image in Fig.S13 . . . . S26
S13 Fibril network of entangled $(1b_4)_n$ nanotubes obtained from chloroform solution on mica surface. S26
S14 Normalized UV spectra of C_{60}@$(1b_4)_n$ (a) and C_{70}@$(1b_4)_n$ (b) in toluene. S27
S15 Molecular models of tetramers $\mathbf{2}_4$ S28
S16 Molecular models of octamers $(\mathbf{2}_4)_2$ S28
S17 ^{1}H NMR spectrum of **7**. S30
S18 ^{13}C NMR spectrum of **7**. S31
S19 COSY NMR spectrum of **7**. S32
S20 HSQC NMR spectrum of **7**. S33
S21 ^{1}H NMR spectrum of **10**. S34
S22 ^{13}C NMR spectrum of **10**. S35
S23 COSY NMR spectrum of **10**. S36
S24 HSQC NMR spectrum of **10**. S37
S25 ^{1}H NMR spectrum of **2**. S38

S26 ^{13}C NMR spectrum of **2**. S39
S27 HSQC NMR spectrum of **2**. S40
S28 ^{1}H NMR spectrum of **11**. S41
S29 ^{13}C NMR spectrum of **11**. S42
S30 COSY NMR spectrum of **11**. S43
S31 HSQC NMR spectrum of **11**. S44
S32 ^{1}H NMR spectrum of **12**. S45
S33 ^{13}C NMR spectrum of **12**. S46
S34 COSY NMR spectrum of **12**. S47
S35 HSQC NMR spectrum of **12**. S48
S36 HMBC NMR spectrum of **12**. S49
S37 ^{1}H NMR spectrum of **14**. S50
S38 ^{13}C NMR spectrum of **14**. S51
S39 COSY NMR spectrum of **14**. S52
S40 HSQC NMR spectrum of **14**. S53
S41 HMBC NMR spectrum of **14**. S54
S42 ^{1}H NMR spectrum of **3**. S55
S43 ^{13}C NMR spectrum of **3**. S56
S44 COSY NMR spectrum of **3**. S57
S45 HSQC NMR spectrum of **3**. S58
S46 HMBC NMR spectrum of **3**. S59
S47 ^{1}H NMR spectra of **1-3** in polymeric state S60

List of Tables

S1 DQ correlations extracted from the ^{1}H – ^{1}H DQ/SQ MAS spectrum of **1b** S17

S2 Single quantum (SQ) ^{1}H chemical shifts for both the chiral monomer and racemic samples, extracted from the DQ/SQ MAS data. (Fig. S2) S19

Materials and Methods

All chemicals were used as received from commercial suppliers. Compounds **1a**[1] and **1b**[2] were prepared according to reported procedures. All moisture sensitive reactions were carried out under an atmosphere of dry nitrogen or argon using oven-dried glassware. Anhydrous tetrahydrofuran was distilled from benzophenone-sodium, dichloromethane was distilled from calcium hydride and toluene was distilled from sodium. Yields refer to chromatographically and spectroscopically homogeneous materials. Reactions were monitored by thin-layer chromatography (TLC) carried out on 0.25 mm Merck silica gel plates (60F-254) using UV light (general), aq. $KMnO_4$ solution (for unsaturated compounds) and vanilin solution (general). For beta-ketoesters, ferric chloride ($FeCl_3$) alcohol solution was used as selective developing agent. Flash column chromatography was performed on Merck silica gel (60, particle size 0.0430-0.663 mm). Melting points were recorded with a Gallenkamp apparatus and are not corrected. ^{1}H and ^{13}C spectra were recorded on Bruker 400 MHz spectrometer. Chemicals shifts are given in parts per million, relative to TMS using the residual solvent peaks at δ= 7.27 (^{1}H NMR) and 77.06 (^{13}C NMR) ppm in $CDCl_3$. The following abbreviations (or combinations thereof) were used to describe ^{1}H NMR multiplicities: s – singlet, d – doublet, t – triplet, q – quartet, m – multiplet, br – broad, app.– apparent. High resolution mass spectra (HRMS) were recorded on Bruker Daltonics microTOF-II or Waters QTOF XEVO-G2 spectrometer equipped with ESI ion source. IR spectra recorded on Perkin Elmer Spectrum BX FT-IR System. Optical rotations were obtained on a KRUSS P3001RS automatic digital polarimeter at 589 nm and $[\alpha]^{20}$ values are given in 10^{-1} deg cm^{-1} g^{-1} and concentrations are given in units of g/100 cm^3.

Abbreviations of chemicals: DCM - dichloromethane, DMPU -1,3-dimethyl-3,4,5,6-tetrahydro-2(1H)-pyrimidinone, EtOAc - ethylacetate, HMPA - Hexamethylphosphoramide, LHMDS - lithium bis(trimethylsilyl)amide, MeOH - methanol, PE - petrol ether (b.p. 40-60°C fraction), TEA - triethylamine, TFA - trifluoroacetic acid, THF - tetrahydrofuran.

S1. Synthesis

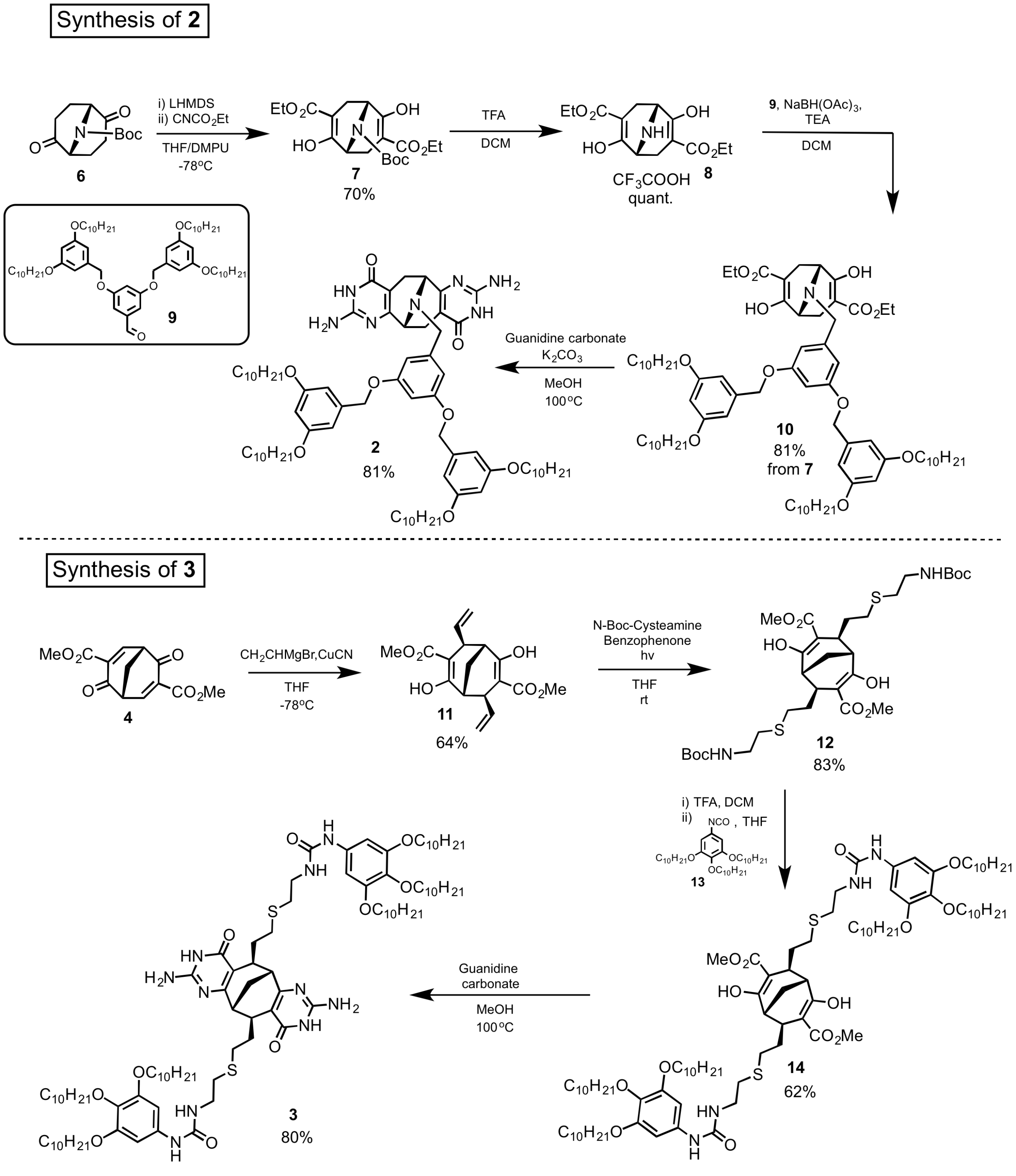

Figure S1: Synthetic routes to target compounds.

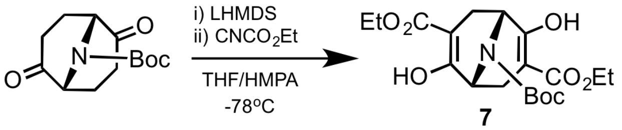

7
70%

Synthesis of 7

To a solution of LiHMDS (15.6 ml, 15.6 mmol, 1M, 2.5 eq.) in THF (60 ml) diketone **6** (1.59 g, 6.26 mmol, 1.0 eq.) in THF (20 ml) was added dropwise at -78°C. Some white precipitate of dienolate of **6** formed shortly after addition and the viscosity of the solution markedly increased. To complete enolization, the mixture was allowed to warm to -40°C and then cooled down to -78°C. HMPA (2.71 ml, 15.6 mmol, 2.5 eq.) was added followed by ethyl cyanoformate (1.54 ml, 15.6 mmol, 2.5 eq.). Reaction mixture was stirred for 15 min at -78°C and quenched by pouring cool reaction mixture into water. Crude product was extracted with DCM (3x10 ml), dried over Na_2SO_4 and concentrated under reduced pressure. Column chromatography on silica gel (35.0 g) eluting with EA/PE (1:50 - 1:30) gradient solvent system afforded 1.76 g (70%) of **7** as a colorless oil.
Note: HMPA can be replaced with less toxic DMPU with *c.a*. 10% reduction in yield.
$[\alpha]_D$ = +20° (c 1.0 in $CHCl_3$)

FTIR (neat, cm^{-1}): 2981, 2933, 1701, 1662, 1284, 1208.

^{1}H NMR (400 MHz, $CDCl_3$/TFA) δ 1.30 (t, *J* = 7.2 Hz, 6Hz, CH_3), 1.46 (s, 9H), 2.55 (dd, *J* = 16.1 Hz, *J* = 7.3 Hz, 2H, CH_2), 2.67 (m, 2H, CH_2), 4.21 (q, *J* = 7.2 Hz, 4H, CH_2(Et)), 4.66 (d, *J* = 5.6 Hz, 1H, CH), 4.83 (d, *J* = 5.6 Hz, 1H, CH), 12.08 (s, 1H, OH), 12.14 (s, 1H, OH).

^{13}C NMR (100 MHz, $CDCl_3$) δ 14.2, 26.3, 26.6, 28.3, 48.3, 49.9, 60.8, 80.9, 95.2, 95.7, 152.8, 169.0, 169.8, 171.9, 172.1.
click here to see Copies of NMR Spectra
HRMS (ESI) calc. for $C_{19}H_{28}NO_8$ $(M+H^+)$: 398.1809, Found: 398.1814.

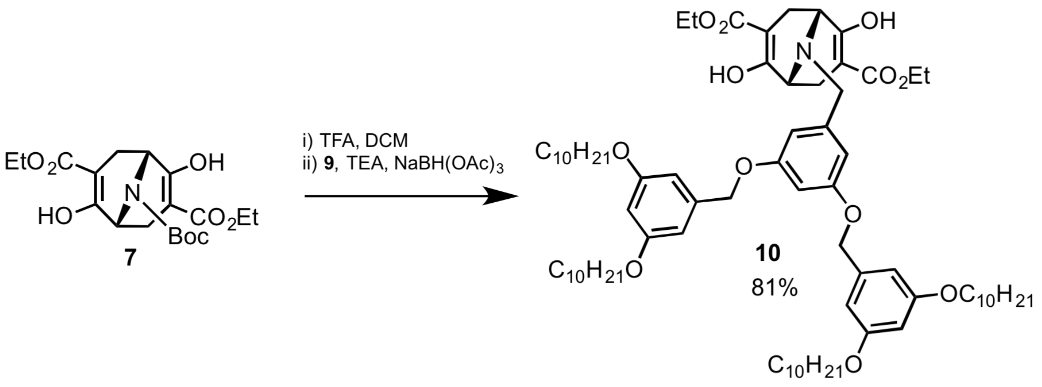

Synthesis of 10

To a solution of **7** (500 mg,1.26 mmol, 1.0 eq.) in DCM (20 ml) was added TFA (5 ml, 50 eq.) dropwise. The mixture was stirred at rt for 3h and then evaporated to dryness. The solid residue was dissolved in dry DCM (20 ml) followed by the addition of **9** (1.78 g, 1.89 mmol, 1.5 eq.) (*Scheme S1*), triethylamine (0.27 ml, 1.89 mmol, 1.5 eq.) and $NaBH(OAc)_3$ (800 mg, 3.77 mmol, 3.0 eq.). The reaction mixture was stirred at rt for 3 days until TLC (EtOAc) shows full consumption of the deprotected **7** ($FeCl_3$ TLC stain). Reaction mixture was then evaporated to dryness and purified by column chromatography on silica gel (PE/EtOAc 35 : 1) to afford 1.25 g (81 %) of **10** as a yellowish solid.

$[\alpha]_D$ = +65° (c 1.51 in $CHCl_3$)

FTIR (neat, cm^{-1}): 2924, 2854, 1660, 1596, 1207, 1164.

^{1}H NMR (400 MHz, $CDCl_3$/TFA) δ 12.03 (s, 2H, OH), 6.61 (d, J = 2.2 Hz, 2H, CH (arom.)), 6.55 (d, *J* = 2.2 Hz, 4H, CH (arom.)), 6.52 (t, J = 2.2 Hz, 1H, CH (arom.)), 6.4 (t, *J* = 2.2 Hz, 2H, CH (arom.)), 4.93 (s, 2H, CH_2 (benzylic)), 4.22 (q, *J* = 7.1 Hz, 4H, OCH_2), 3.93 (t, *J* = 6.6 Hz, 8H, CH_2), 3.64 (ABq, $\Delta\delta$AB= 0.08, *J* = 13.5 Hz, 2H, CH (benzylic)), 3.43 (d, *J* = 5.9 Hz, 2H, CH), 2.64 (dd, *J* = 16.0 Hz, *J* = 7.0 Hz, 2H, CH_2), 2.41 (d, *J* = 16 Hz, 2H, CH_2), 1.76 (p, *J* =6.6 Hz, 8H, CH_2), 1.18-1.50 (m, 60H, CH_2 and CH_3(Et)), 0.88 (t, *J* = 6.6 Hz, 12H, CH_3).

^{13}C NMR (100 MHz, $CDCl_3$) δ 1172.2, 170.5, 160.96, 160.5, 160.1, 140.2, 139.0, 107.6, 105.8, 101.0, 100.9, 94.8, 70.2, 68.1, 60.6, 56.0, 54.0, 31.9, 29.6, 29.5, 29.4, 29.3, 29.2, 26.1, 24.8, 22.7, 14.2, 14.1.

click here to see Copies of NMR Spectra

HRMS (ESI) calc. for $C_{75}H_{118}NO_2$ (M+H$^+$): 1224.8649; Found: 1224.8654.

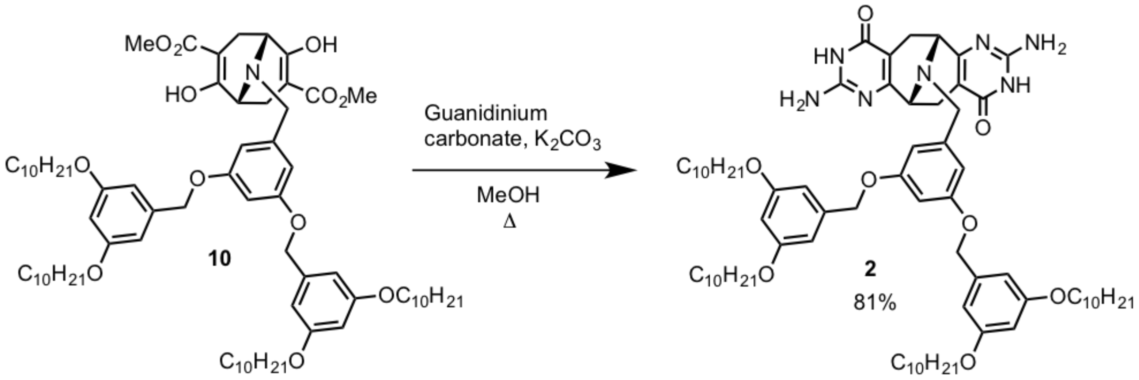

Synthesis of 2

A mixture of β-keto ester **10** (1.156 g, 0.92 mmol, 1.0 eq.), guanidine carbonate (442 mg, 4.61 mmol, 5.0 eq.) and K_2CO_3 (442 mg, 4.61 mmol, 5.0 eq.) in MeOH (20 ml) was heated under reflux overnight. Reaction mixture was cooled down, diluted with water and extracted with CH_2Cl_2 (3 x 5 ml). Combined organic phases were dried over Na_2SO_4 and concentrated under reduced pressure. Crude material was dissolved in minimum amount $CHCl_3$, precipitated with MeOH and filtered. Filter cake was washed with MeOH to afford 906 mg (81 %) of **2** as off-white powder.

$[\alpha]_D$ = +15° (c 0.1 in $CHCl_3$)

mp (enantiopure) > 230 °C (decomp.), (rac) > 280 °C (decomp.)

FTIR (neat, cm^{-1}): 3329, 3152, 2924, 2853, 1654, 1596, 1458, 1166, 1054.

^{1}H NMR (400 MHz, $CDCl_3$/TFA) δ 11.14 (s, 2H, NH), 6.75 (s, 1H, CH (arom.)), 6.61 (s, 6H, CH (arom.)), 6.54 (s, 2H, CH (arom.)), 5.19 (br, 2H, CH_2), 4.49 (d, *J* =13.0 Hz, 1H, CH), 4.36 (d, *J* =13.0 Hz, 1H, CH), 4.03 (t, *J* =7.0 Hz, 8H, CH_2), 3.34 (d, *J* =18.0 Hz, 2H, CH_2), 3.12 (d, *J* =18.0 Hz, 2H, CH_2), 1.78 (t, *J* =7.0 Hz, 8H, CH_2), 1.16-1.50 (m, 56 H, CH_2), 1.87 (t, *J* =7.0 Hz, 12H, CH_3).

^{13}C NMR (100 MHz, $CDCl_3$) δ 162.8, 162.3, 161.9, 161.5, 160.8, 160.2, 159.7, 152.1, 144.4, 138.1, 127.86, 118.5, 115.7, 112.9, 110.0, 107.5, 106.54, 104.3, 102.6, 70.5, 57.6, 51.5, 31.9, 29.6, 29.3, 28.9, 25.8, 22.7, 13.9.

click here to see Copies of NMR Spectra

HRMS (ESI) calc. for $C_{73}H_{112}N_7O_8$ (M+H$^+$): 1214.8567; Found: 1214.8582.

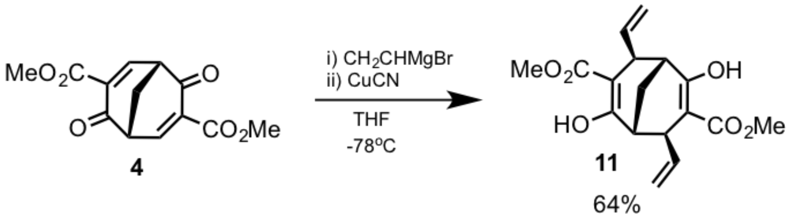

Synthesis of 11

To a suspension of CuCN (301 mg, 3.36 mmol, 4.2 eq.) in dry THF (4.0 ml) vinyl magnesium soliution (3.2 ml, 1.0 M in THF, 3.32 mmol, 4.15 eq.) was added dropwise at -20°C. The temperature was gradually raised to c.a. -10°C resulting in deep brown colour. The mixture was cooled to -78°C and a solution of bicyclo[3.3.1]nona-3,7-diene-2,6-dione **4** (0.21 g, 0.8 mmol) in dry THF (4.0 ml) was added dropwise. After 10 min, cooling bath was removed and the reaction mixture was quenched with 1.0 M HCl solution (15 mL). The mixture was extracted with EtOAc, combined organic phase was diluted with H_2O and filtered through CELITE. Organic phase was washed with $NaHCO_3$, dried over Na_2SO_4 and concentrated. The residue was purified by flash chromatography (PE/EtOAc 95:5) to afford 175 mg (64%) of **11** as a colourless solid.

$[\alpha]_D$ = +103° (c 1.0 in $CHCl_3$)

FTIR (neat, cm^{-1}): 2955, 1645, 1603, 1442,1210.

mp 81-82 °C

^{1}H NMR (400 MHz, $CDCl_3$) δ 12.49 (s, 2H, OH), 6.00-5.85 (m, 2H, C=CH), 5.15 (dt, *J* =10.2 Hz, *J* =1.2 Hz, 2H, C=CH_2), 5.01 (dt, *J* =17.1 Hz, *J* =1.2 Hz, 2H, C=CH_2), 3.73 (s, 6H, $COOCH_3$), 3.50 (app. d, *J* =5.3 Hz, 2H, CH), 2.49-2.45 (m, 2H, CH), 1.79 (app. t, *J* =3.0 Hz, 2H, CH_2).

^{13}C NMR (100 MHz, $CDCl_3$) δ 174.1, 173.0, 139.8, 115.9, 97.7, 51.5, 40.7, 38.3, 17.5.

click here to see Copies of NMR Spectra

HRMS (ESI) calc. for $C_{17}H_{21}O_6$ ($M+H^+$): 321.1338; Found: 321.1331.

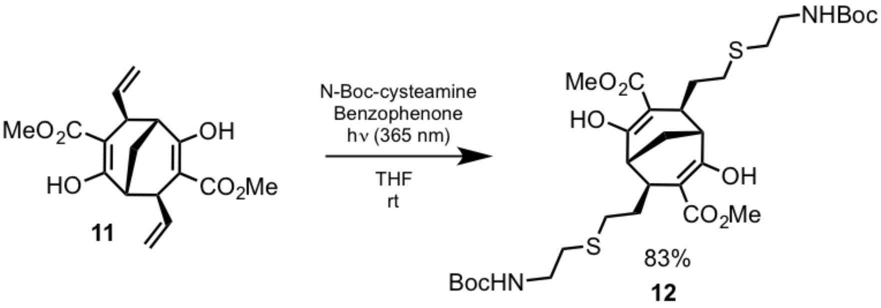

Synthesis of 12

A glass test tube was charged with **11** (386 mg, 1.20 mmol, 1.00 eq.), N-Boc-cysteamine (963 mg, 5.43 mmol, 4.50 eq.) and benzophenone (227 mg, 1.24 mmol, 1.03 eq.) under N_2. Dry THF (13 mL) was added and the solution was irradiated with UV lamp (365 nm, 8W) for 8 h. Then, solvent was removed under reduced pressure and the residue was purified by flash chromatography (PE/EtOAc 40 : 1 to 8: 1) to afford 677 mg (84%) of **12** as a colourless oil.

$[\alpha]_D$ = +60.3° (c 1.16 in $CHCl_3$)

FTIR (neat, cm^{-1}): 3324, 2922, 2853, 1645, 1113.

^{1}H NMR (400 MHz, $CDCl_3$) δ 12.33 (s, 2H, OH), 5.01 (bt, 2H, NH), 3.77 (s, 6H, $COOCH_3$), 3.39-3.29 (m, 4H, N-CH_2), 2.78 (app.d, J =9.8 Hz_3, 2H, CH), 2.68 (overlaping t, 6H, CH(H) and CH_2), 2.65-2.55 (m, 2H, CH(H)), 2.49 (br. s, 2H, CH), 2.01-1.89 (m, 2H, CH(H)), 1.83 (br. s, 2H, CH_2), 1.66-1.54 (m, 2H, CH(H)), 1.46 (s, 9H, Boc).

^{13}C NMR (100 MHz, $CDCl_3$) δ 173.7, 172.8, 100.1, 79.4, 51.6, 39.7, 36.9, 36.0, 33.4, 32.3, 30.0, 28.4, 18.4.

click here to see Copies of NMR Spectra

HRMS (ESI) calc. for $C_{31}H_{50}N_2O_{10}S_2$ ($M+Na^+$): 697.2805, Found: 697.2817.

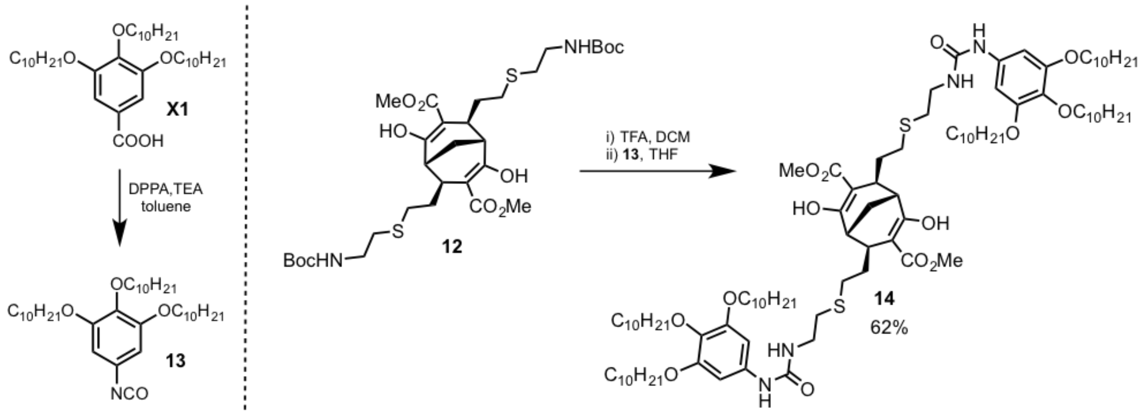

Synthesis of 14

*Preparation of isocyanate **13***. A mixture of acid **X1** (315 mg, 0.57 mmol, 1.0 eq.), triethylamine (148 μl, 1.04 mmol, 2.0 eq.) and diphenylphosphoryl azide (133 μl, 0.61 mmol, 1.15 eq.) in dry toluene (7.0 ml) was heated at 40°C for 1.5 h. Then, the temperature was increased to 90°C and heating was continued for 4 h. After cooling to room temperature, the solvent was removed under reduced pressure and the residue was dried in high vacuum. The crude isocyanate **13** so-obtained was used in the next step without further purification.

Compound **12** (80 mg, 0.13 mmol, 1.0 eq.) was dissolved in dry DCM (5.0 ml) and TFA (0.5 ml) was added dropwise while cooling reaction mixture in an ice bath. After addition, the mixture was allowed to slowly reach the room temperature (without removing the ice bath). Then, the reaction mixture was stirred for an additional hour. The volatiles were removed under reduced pressure and the residue was dried under high vacuum. The crude salt obtained above was dissolved in THF (8.0 ml) followed by addition of triethylamine (0.2 ml). The solution obtained was added to isocyanate **13** (see the procedure above) and the mixture was heated at 50°C in a sealed vial overnight. The solvent was removed under reduced pressure and the residue was purified by flash chromatography (PE to DCM/MeOH 60 : 1) to afford 121 mg (62%) of **14** as a colourless oil.

FTIR (neat, cm^{-1}): 2923, 2854, 1606, 1504, 1225, 1203.

^{1}H NMR (400 MHz, $CDCl_3$) δ 12.52 (s, 2H, OH), 6.78 (s, 2H, NH), 6.58 (s, 4H, CH (arom.)), 5.49 (t, *J* =5.5 Hz, 2H, NH), 3.96-3.87 (m, 12H, -OCH_2), 3.76 (s, 6H, $COOCH_3$), 3.61-3.34 (m, 4H, CH_2), 2.82 (app. d, 2H, CH), 2.78-2.64 (m, 6H, CH_2 and CH(H)), 2.62-2.52 (m, 2H, CH(H)), 2.50 (br. s, 2H, CH), 1.98-1.87 (m, 2H, CH(H)1, 1.85-1.66 (m, 14H, CH_2), 1.65-1.54 (m, 2H, CH(H)), 1.52-1.39 (m, 12H, CH_2), 1.39-1.18 (m, 72H, CH_2), 0.95-0.83 (m, 18H, CH_3).

^{13}C NMR (100 MHz, $CDCl_3$) δ 173.6, 172.9, 155.8, 153.4,134.4, 134.1, 100.5, 99.8, 73.6, 69.1, 51.8, 39.28, 36.6, 35.9, 33.1, 32.3, 31.9, 30.3, 30.1, 29.8, 29.7, 29.5, 29.42, 29.38, 26.1, 22.7, 18.3 18.4.

click here to see Copies of NMR Spectra

HRMS (ESI) calc. for $C_{95}H_{164}N_4O_{10}S_2$ (M+H$^+$): 1650.1764, Found: 1650.1772.

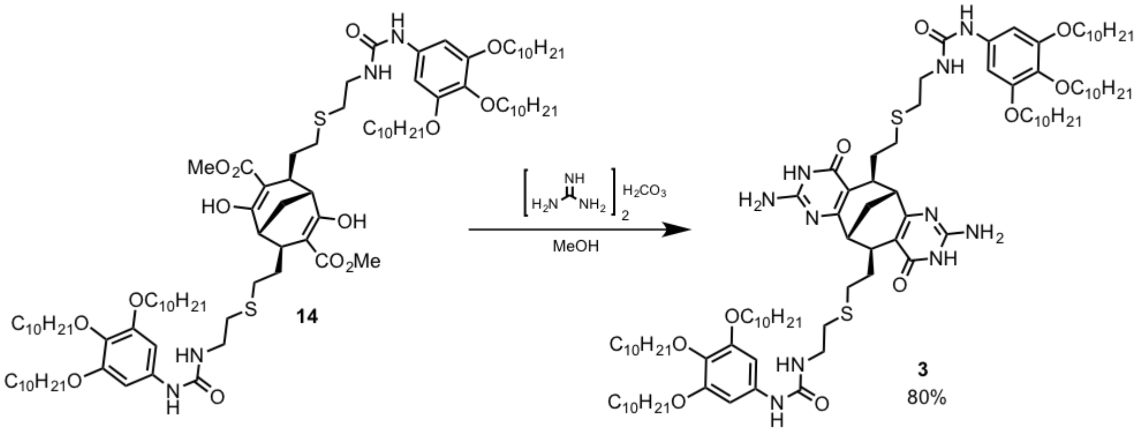

Synthesis of 3

A mixture of **14** (573 mg, 0.347 mmol, 1.0 eq.) and guanidinium carbonate (625 mg, 6.94 mmol, 20.0 eq.) in MeOH (15 ml) was heated at 80°C in a sealed vial overnight. The solvent was removed under reduced pressure and the residue was quenched with excess of 1.0 M HCl (aq.) solution. The water phase was extracted with Et_2O several times. The organic phase was washed with sat. $NaHCO_3$ solution and dried with Na_2SO_4. The solution was concentrated under reduced pressure and the residue was purified by flash chromatography (DCM/MeOH from 250 : 1 to 30 : 1) to afford 450 mg (80%) of **3** as a colourless glass.

FTIR (neat, cm^{-1}): 2922, 2853, 1644, 1603, 1503, 1226, 1113.

^{1}H NMR (400 MHz, $CDCl_3$/TFA) δ 6.46 (s, 4H, CH (arom.)), 4.03 (t, J =6.8 Hz, 4H, -OCH_2-), 3.94 (t, J =5.9 Hz, 8H, -OCH_2-), 3.49 (br.t, 2H, NH), 3.02 (br. s, 2H, CH), 2.92-2.80 (m, 4H, CH (H) and CH), 2.79-2.63 (m, 6H, CH (H) and CH_2), 2.10 (br. s, 2H, CH_2), 2.06-1.96 (m, 2H, CH (H)), 1.84-1.71 (m, 12H, CH_2), 1.71-1.57 (m, 2H, CH(H)), 1.50-1.39 (m, 12H, CH_2), 1.39-1.19 (m, 72H, CH_2), 0.95-0.84 (m, 18H, CH_3).

^{13}C NMR (100 MHz, $CDCl_3$/TFA) δ 160.8, 159.1, 153.6, 151.9, 151.2, 135.4, 131.3, 113.0, 103.6, 74.7, 69.4, 39.5, 36.6, 32.7, 31.90, 31.87, 31.6, 29.8, 29.7, 29.61, 29.57, 29.55, 29.5, 29.3, 29.1, 26.0, 25.9, 22.7, 22.6, 14.1 .

click here to see Copies of NMR Spectra

HRMS (ESI) calc. for $C_{95}H_{162}N_{10}O_{10}S_2$ (M+H$^+$): 1668.1990; Found: 1668.1995.

S2. Solid State NMR

All solid-state magic-angle spinning (MAS) NMR experiments were performed at either 20.0 (Bruker Avance III) or 14.1 (Bruker Avance II+) T, operating at a ^{1}H Larmor frequency of 850 and 600 MHz, respectively. Experiments were performed using a 1.3 mm triple-resonance probe (operating in double-resonance mode), except for CP MAS experiments whereby a 3.2 or 4 mm probe was used. In all cases, the ^{1}H $\pi/2$ pulse duration was 2.5 μs. Sign discrimination in the F1 dimension of 2D experiments was achieved using the States-TPPI (DQ/SQ MAS) or States methods. Note that, in 2D spectra, positive and negative contours are shown in black and red, respectively. For ^{1}H - ^{1}H DQ/SQ MAS experiments, a 16-step phase cycle was used to select $\Delta p = \pm 2$ on the DQ excitation pulses (4 steps) and $\Delta p = \pm 1$ (4 steps) on the z-filter /2 pulse, where p is the coherence order. For ^{14}N – ^{1}H HMQC experiments a 4-step nested phase cycle was used to select changes in coherence order $\Delta p = \pm 1$ (on the first ^{1}H pulse, 2 steps) and $\Delta p = \pm 1$ (on the last ^{14}N pulse, 2 steps). In all experiments presented, ^{1}H and ^{13}C chemical shifts were referenced with respect to neat TMS using L-alanine as a secondary reference: 1.3 ppm for the CH_3 ^{1}H resonance, corresponding to 1.85 ppm for adamantane, [3], and 20.5 ppm for the CH_3 ^{13}C resonance, corresponding to 38.5 ppm for adamantane [4]. ^{14}N chemical shifts were referenced relative to neat CH_3NO_2 using the ^{14}N resonance of NH_4Cl (powdered solid) at 341.2 ppm as an external reference [5]. To convert to the chemical shift scale frequently used in protein NMR, where the alternative IUPAC reference (see Appendix 1 of ref [6]) is liquid ammonia at 50 °C, it is necessary to add 379.5 ppm to the given values.[7]. ^{1}H, ^{13}C, ^{14}N and ^{15}N shifts can be specified to an accuracy of ±0.2, ±0.1, ±5 and ±0.1 ppm, respectively.

^{1}H - ^{1}H DQ/SQ MAS experiments.[8], [9] One rotor period of BABA recoupling[10], [11] was used for the excitation and reconversion of DQ coherence. For each of 256 t_1 FIDs, 16 transients were co-added with a recycle delay of 6 seconds. The $F_1 = 2F_2$ diagonal is indicated as a dashed black line. The base contour level is shown at 1% of the maximum peak intensity.

^{14}N - ^{1}H HMQC experiments.[9],[12], [13] The spectrum was recorded using the R^3 recoupling scheme [14] for the recoupling of the ^{14}N – ^{1}H heteronuclear dipolar couplings for a τ_{RCPL} = 107 μs. For each of 32 t_1 FIDs, 128 transients were coadded with a recycle delay of 6 seconds. The base contour level is shown at 26% of the maximum peak intensity.

^{1}H - ^{13}C and ^{1}H - ^{15}N CP MAS experiments. Ramped CP[15] was employed for a contact time of 1 ms (^{1}H – ^{13}C), 5 ms (^{1}H – ^{15}N, enantiopure) or 2 ms (^{1}H – ^{15}N, racemic). SPINAL-64 ^{1}H decouping[16] with a pulse length of 6.0 μs (^{1}H – ^{13}C), 5.8 μs (^{1}H – ^{15}N, enantiopure) or 4.8 μs (^{1}H – ^{15}N, racemic) at a ^{1}H nutation frequency of 100 kHz was applied during the acquisition of the FID. The ^{1}H – ^{13}C spectrum was recorded with 11264 co-added transients and a recycle delay of 6 seconds. The ^{1}H – ^{15}N spectra were recorded with 30720 (enantiopure) or 22528 (racemic) co-added transients and

a recycle delay of 3 seconds.

Table S1: DQ correlations extracted from the ^1H – ^1H DQ/SQ MAS spectrum of **1b** (see Fig.3)

Entry	correlation	Sum of SQ freq. (ppm)	DQ freq. (ppm)
1	CH_3-CH_3	0.9+0.9	1.8
2	CH_2-CH_2	1.1+1.1	2.2
3	CH_2-CH	1.1+2.8	3.9
4	CH_2-H_a	1.1+7.4	8.5
5	CH_2-H_b	1.1+10.3	10.4
6	CH-H2	2.8+12.1	14.9
7	H_a-H_b	7.4+10.3	17.7
8	CH_3-H1	0.9+17.0	17.9
9	H_a-H2	7.4+12.1	19.5
10	H_b-H1	10.3+17.0	27.3

^{14}N chemical shifts

NH_2 : -65 ppm

N – H2 : -75 ppm

N – H1 : -105 ppm

S2.2 Racemic vs chiral monomer

Compared to enantiopure **1b**, racemic **1b** is completely insoluble in chlorinated and aromatic solvents, indicating its different aggregation mode. Very similar ^1H and ^{15}N solid-state NMR spectra (see Figs.S2 and S3) were obtained for racemic **1b** and enantiopure **1b**, with slightly different chemical shift values (Table S2) and better resolution of Ha and Hb protons of the isocytosine amino group. The presence of two tautomeric forms of isocytosine in racemic **1b** is easily explained assuming an energetically favourable 3H-bonding interaction of isocytosine units between two enantiomers that enable the formation of 1D zig-zag polymeric structures. These polymers can further assemble in orthogonal directions to form insoluble corrugated sheets (Fig. S4).

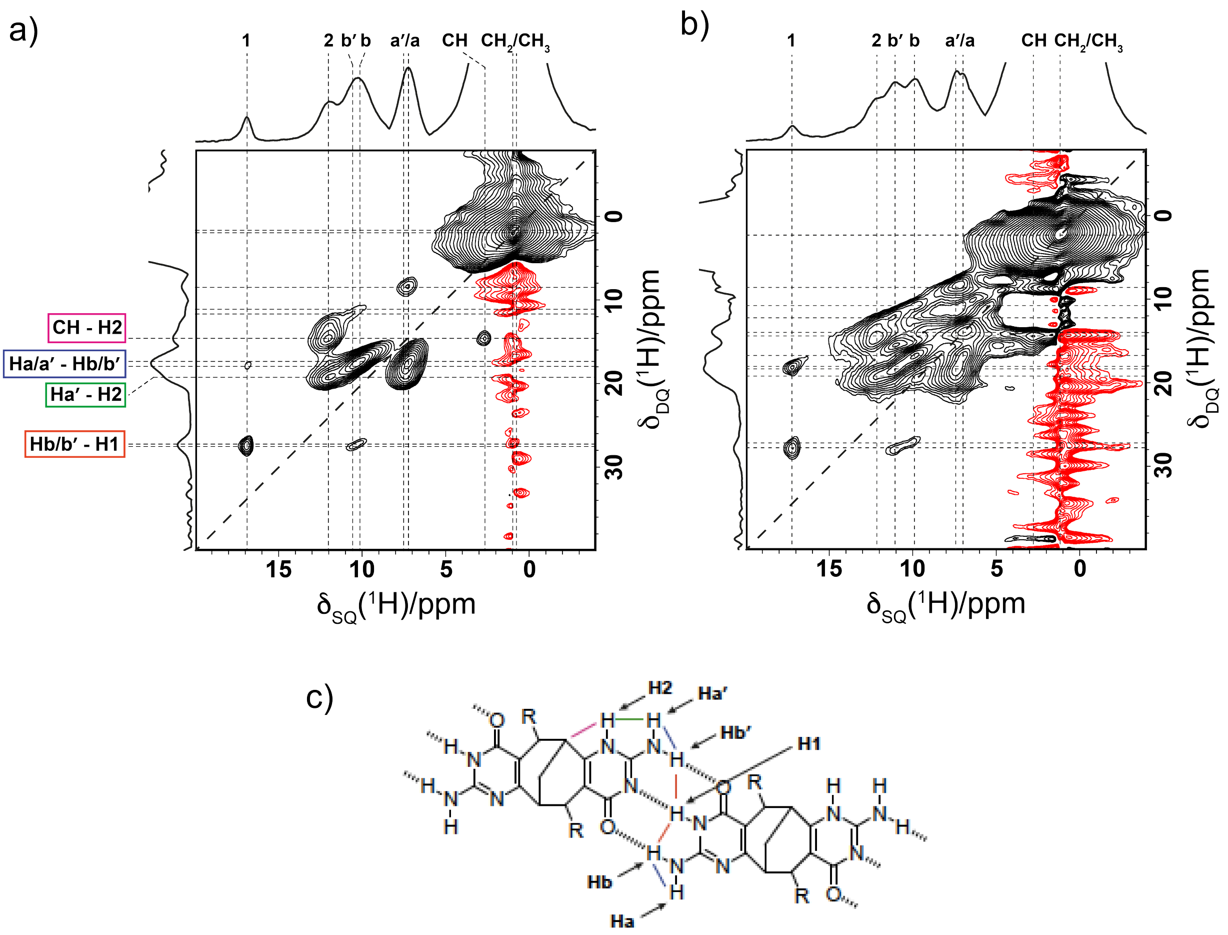

Figure S2: Two-dimensional solid state ^1H - ^1H DQ/SQ MAS (60 kHz MAS) spectrum of (a) enantiopure **1b** (850 MHz) and (b) racemic **1b** (600 MHz) with assignments. Characteristic through-space interactions are indicated with dashed lines.

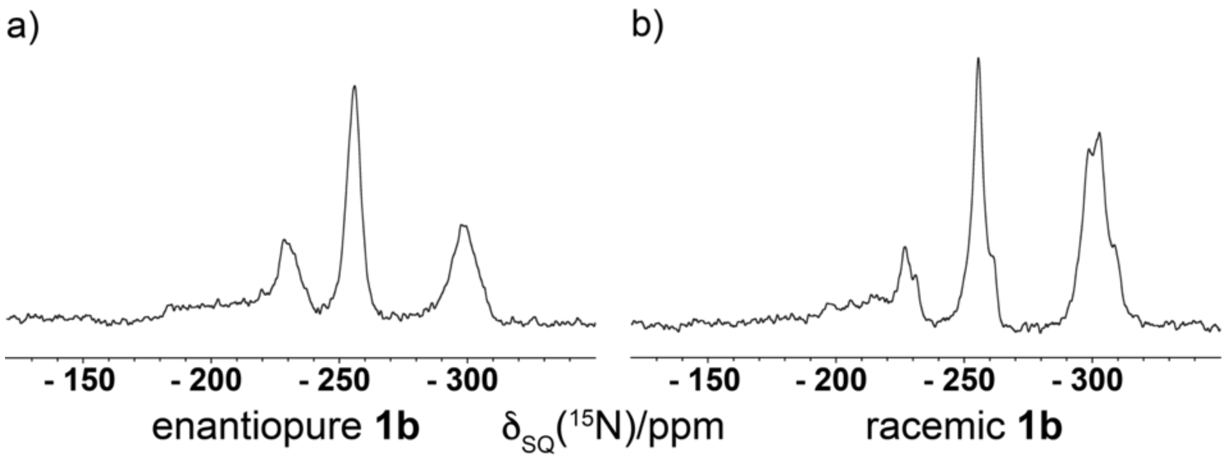

Figure S3: ^{1}H (600 MHz)-^{15}N CP MAS (10 kHz) spectra of (a) enantiopure **1b** and (b) racemic **1b**

Table S2: Single quantum (SQ) ^{1}H chemical shifts for both the chiral monomer and racemic samples, extracted from the DQ/SQ MAS data. (Fig. S2)

Enantiopure **1b**		Racemic **1b**	
Site	$\delta_{SQ}(^{1}H)$/ppm	Site	$\delta_{SQ}(^{1}H)$/ppm
H1	16.9	H1	17.2
H2	11.9	H2	12.2
H_b'	10.6	H_b'	11.1
H_b	10.1	H_b	9.9
H_a'	7.5	H_a'	7.3
H_a	7.2	H_a	7.0
CH	2.7	CH	2.8
CH_2	1.0	CH_2	1.2
CH_3	0.8	CH_3	1.0

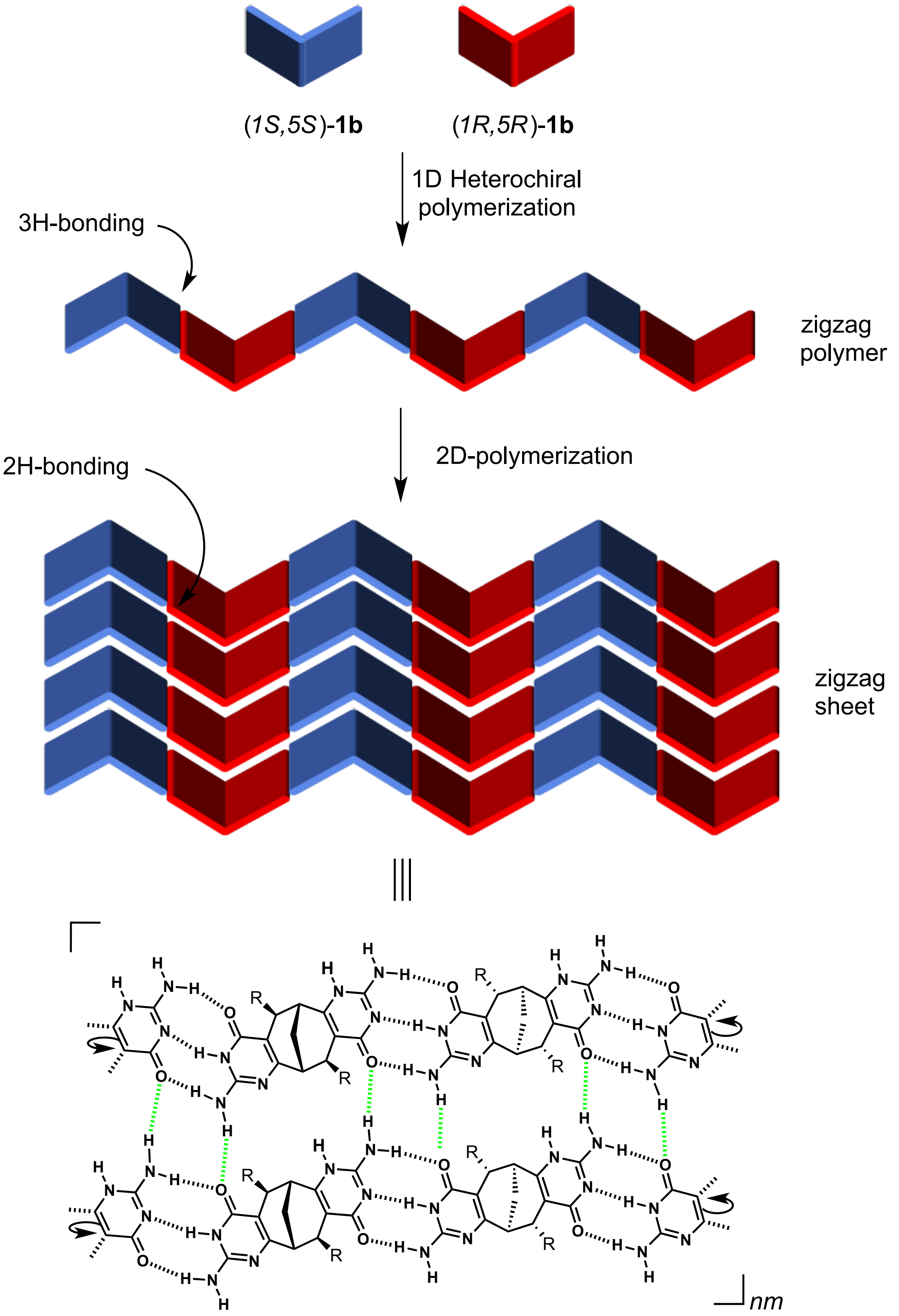

Figure S4: Schematic representation of the formation of insoluble corrugated sheets via 2D 3H- heterochiral and 2H-homochiral bonding between the enantiomers of **1b**. Solubilizing chains are omitted for clarity.

S3. Dynamic Light Scattering

Dynamic light scattering (DLS) experiments were carried out on a Zetasizer Nano Z (Malvern) instrument at 298K. Samples were prepared by dissolving the corresponding amount of monomers **1b-3** in toluene, filtering through PTFE membrane filter (AcroDisc, 0.12 micron) and aging for 24 hours. Measurements were at least duplicated and the data with good quality correlograms were used.

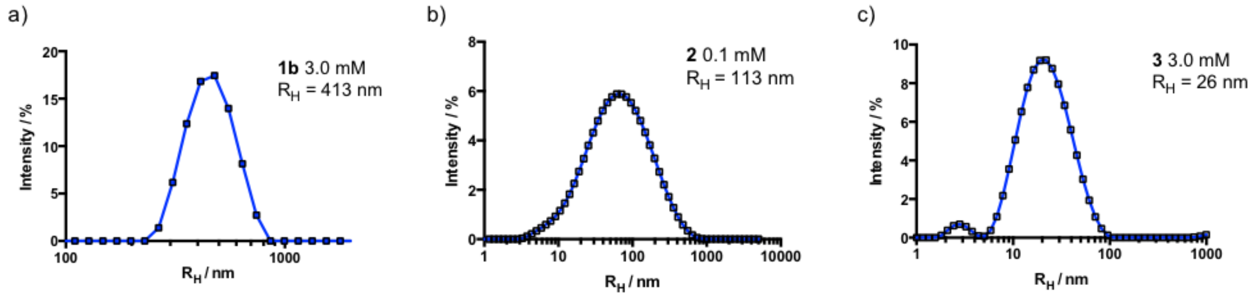

Figure S5: DLS data (R_H – hydrodynamic radius) for monomers **1b** (a), **2** (b) and **3** (c) in toluene solution. Concentrations and mean sizes of aggregates are indicated on the graphs. In case of monomer **3**, bimodal distribution was observed, showing the presence of small amount of cyclic tetramers (R_H 2-3 nm).

It should be noted that for compound **2**, much smaller aggregates with $R_H < 10$ nm were obtained in chloroform solution as opposed to large polymeric assemblies in toluene (Fig. S6). This observation is in line with significantly weaker H-bonds in chlorinated solvents as compared to aromatic ones. Smaller degree of polymerization in this solvent was also evident from more resolved ^{1}H NMR spectrum as opposed to very broad spectrum in toluene (see Fig. S47).

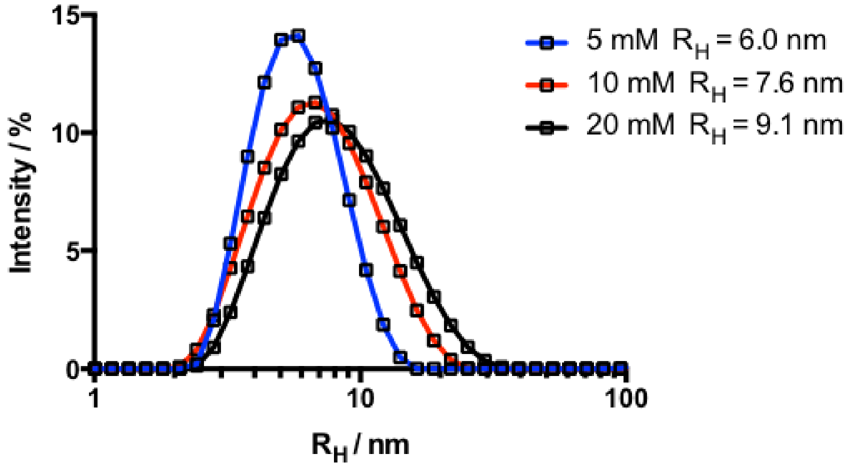

Figure S6: Concentration dependence of hydrodynamic radii of $(\mathbf{2}_4)_n$ aggregates in chloroform solution. Concentrations and mean sizes of aggregates are indicated on the graph

S4. Viscosimetry

Viscosity was measured on AMVn Automated Micro Viscosimeter (Antor Paar) using 2.5 mm diameter gold coated ball at 298 K except for monomer **2**, viscosity of which was probed at three different temperatures (298K, 338K and 258K). The corresponding solutions of monomers **2** and **3** were made in toluene, whereas chloroform was used for **1b**due to solubility reasons.

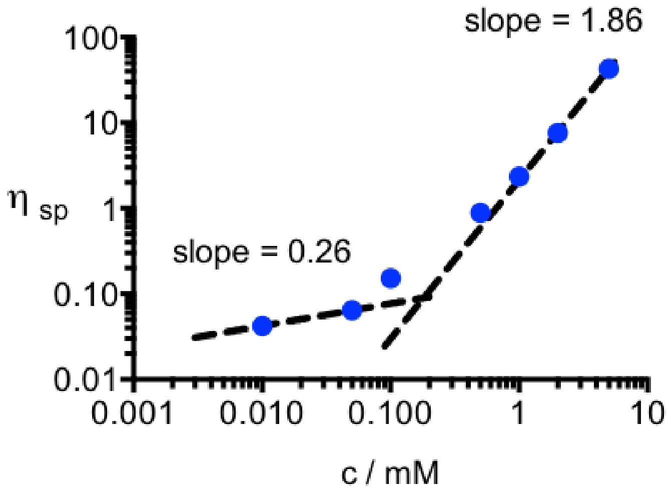

Figure S7: log-log plot of specific viscosity *vs* concentration for **2** in toluene (298K).

Two distinct regimes can be identified in double logarithmic plot of **2** in toluene (Fig. S7). The low-slope regime can be attributed to a solution dominated by cyclic tetramers, whereas higher-slope dependence is caused by supramolecular polymerization of cyclic tetramers. In case of monomer **1b** in chloroform, single-slope regime is observed, starting at very low concentration, which indicates very efficient supramolecular polymerization (Fig. S8).

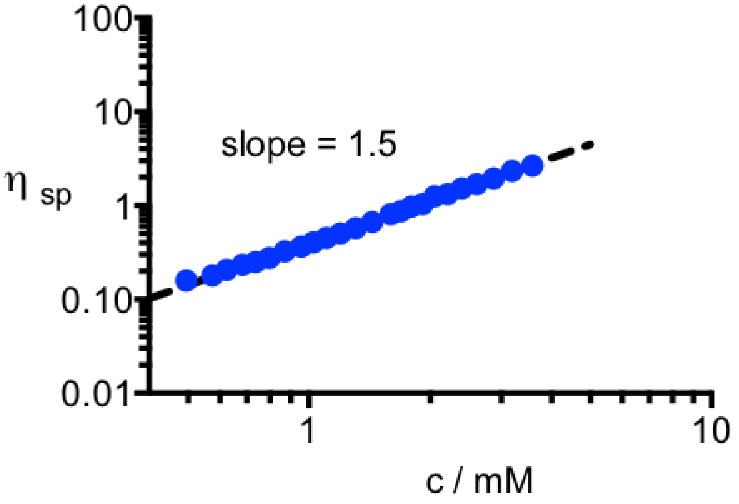

Figure S8: log-log plot of specific viscosity vs concentration for **1b** in chloroform (298K).

High cooperativity of aggregation of monomer 1b in chloroform was demonstrated using viscosity measurements in the presence of simple isocytosine derivative ChS (Fig. S9), which might serve as a chain stopper for both tautomeric forms of the isocytosine. No change in viscosity was observed even in the presence of 10 mol % (solubility limit of ChS) of chain stopper.

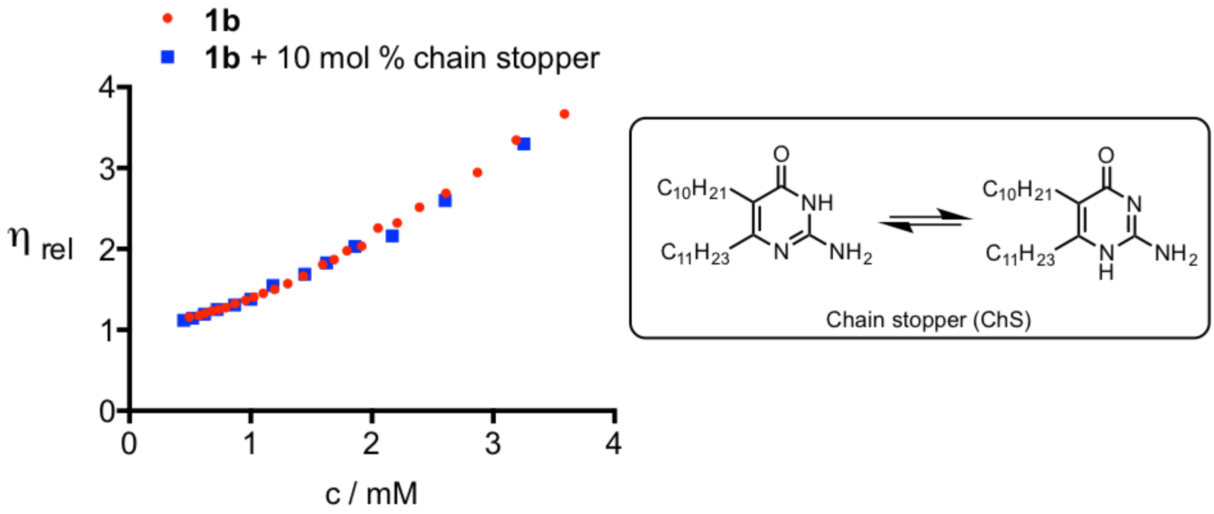

Figure S9: Viscosity-concentration plot for **1b** in chloroform (red) and in the presence of chain stopper (ChS) (blue).

S5. Preparation of Gels

Known amount of monomers **1b** or **2** were added into 4.0 ml glass vial containing a stirring bar followed by solvent (methanol-free $CHCl_3$ or toluene). The vial was screw-capped with a plastic cap containing heat resistant PFET membrane. The vial was immersed into preheated oil bath (80°C for $CHCl_3$ and 100°C for toluene) and stirred until clear solution was obtained. The sol produced was cooled to ambient temperature and left undisturbed for 48 hr. The gelation concentration corresponds to the lowest concentration of monomer at which self-supporting gel is obtained. Gelation experiments in the presence of C_{60}/C_{70} were done by adding known amount of toluene to a mixture of 4.0 equiv. of **1b** or **2** and 1.0 equiv. C_{60}/C_{70} (17.6 mg of **1b** (19.00 mmol) and 5.6 mg C_{60} (4.7 mmol) in 1.4 ml toluene; 9.3 mg of **1b** (14.0 mmol) and 3.4 mg C_{70} (3.5 mmol) in 1.0 ml toluene; 10.0 mg of **2** (8.2 mmol) and 1.5 mg C_{60} (2.05 mmol) in 1.0 ml toluene). Heating of the mixture as above was continued until clear sol was obtained (Fig. S10). Significant increase of C_{60} and C_{70} solubility in toluene was observed.

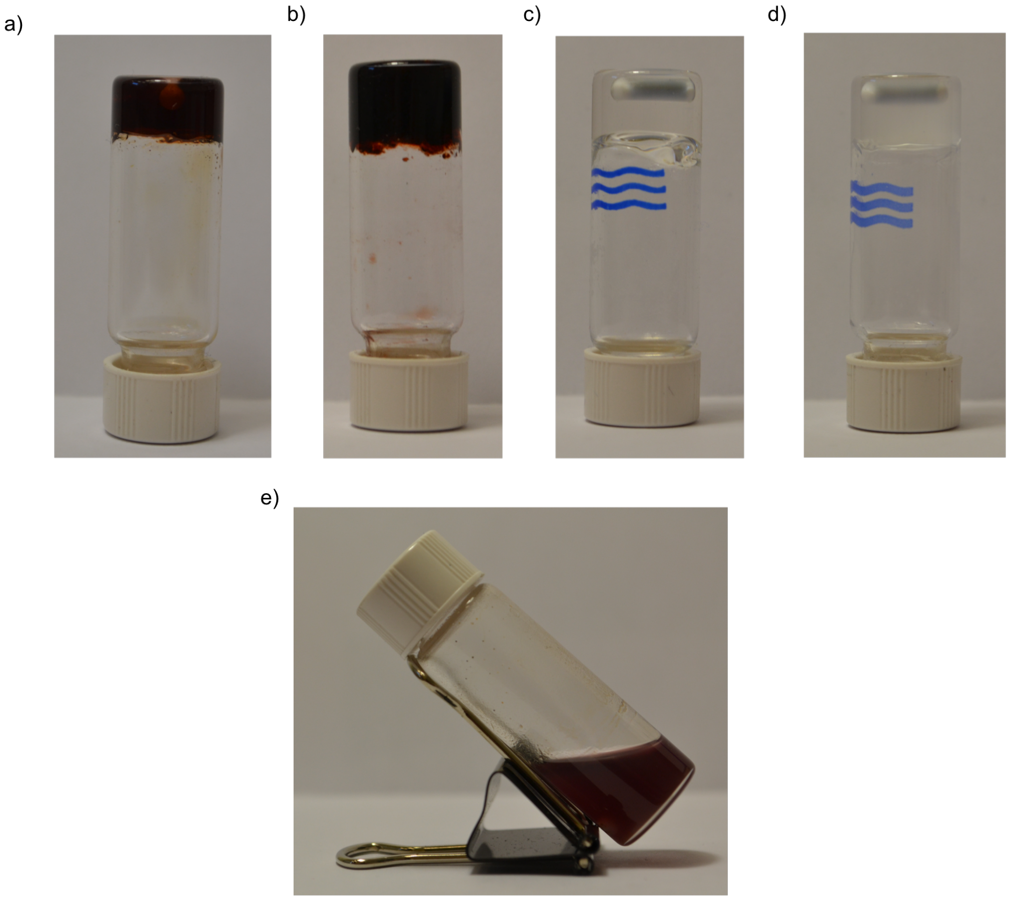

Figure S10: Self-sustainable gels of C_{60}@$(1b_4)_n$ in toluene (a), C_{70}@$(1b_4)_n$ in toluene (b), $(1b_4)_n$ (c) in toluene, $(1b_4)_n$ in chloroform (d) and suspension of racemic $(1b_4)_n$ and C_{60} in toluene.

S6. Atomic Force Microscopy

AFM imaging was carried out in ambient conditions using the JPK NanoWizard 3 AFM in intermmitent contact mode using the RTESPA (Bruker), Scout 350 (Nu Nano) and Tap300Al-G (Budget-Sensors) probes. The samples were prepared by drop casting the solution on the freshly cleaved muscovite mica and drying in nitrogen stream. The sample in manuscript Fig. 4a was dried 30s after drop casting. The sample in manuscript Fig. 4b was prepared by ultrasonicating the solution prior drop casting and drying in nitrogen stream after 10 s. Fibril structures were also observed in AFM images of $(1b_4)_n$ and $(3_4)_n$ from toluene solutions (Fig. S11).

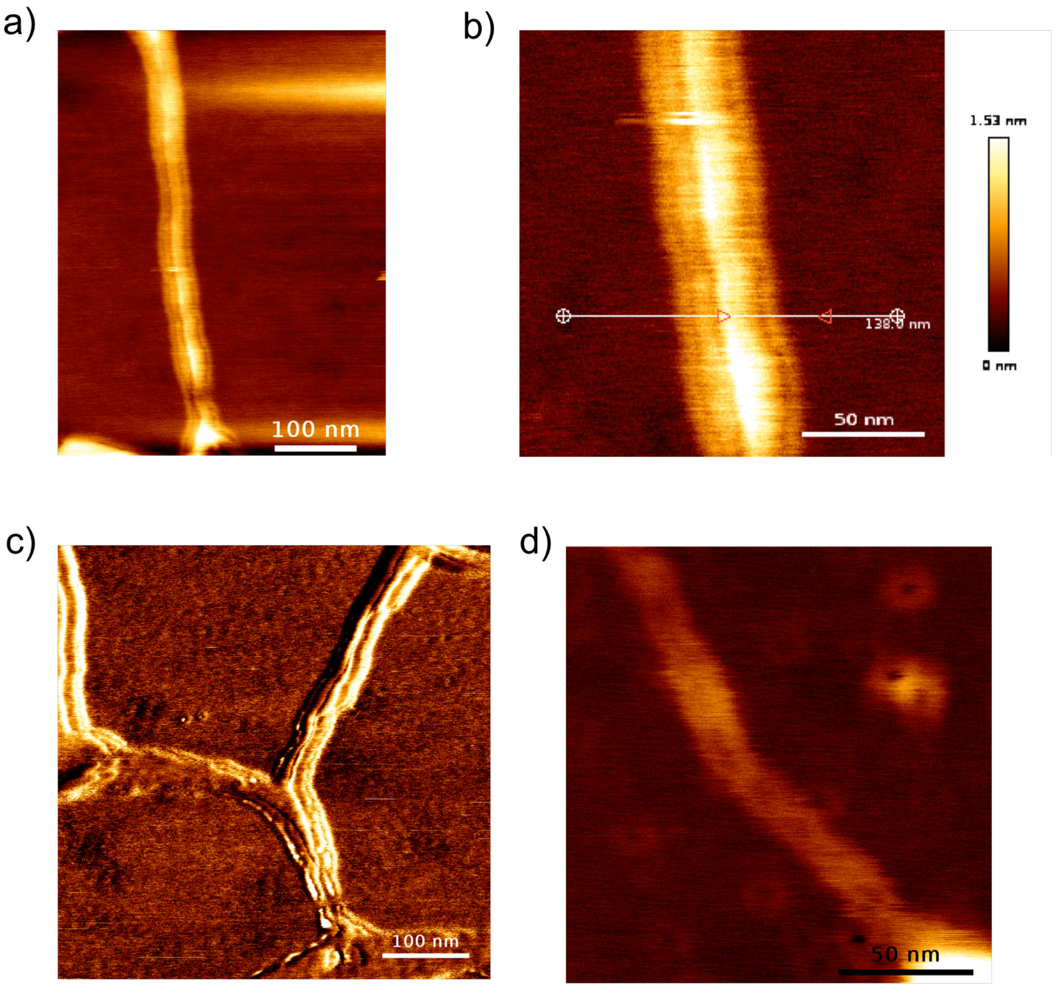

Figure S11: AFM images (toluene solution) of $(1b_4)_n$ (a-b), phase-contrast AFM image of $(1b_4)_n$ (c) and $(3_4)_n$ (d).

The AFM images of molecular tubes at lower concentrations suggest that the mica substrate has a templating effect on the fibrils. In case where individual fibrils are observed, the preferential alignment of fibrils along the mica crystallographic axes is seen. In case of entangled network of fibrils at higher concentration, the preferential angles seem to be determined by a combined action of surface templation and the fibril-fibril interaction, resulting in broader distribution of

preferred alignment angles. These preferences were revealed by applying histogram of oriented gradients [17] analysis (Fig. S12) on Fig. S13, with notable peaks at 63, 122, 55, 115 degrees. Large peaks at 0 and 90 degrees most likely result from the areas in the image where the fibrils are overlaid.

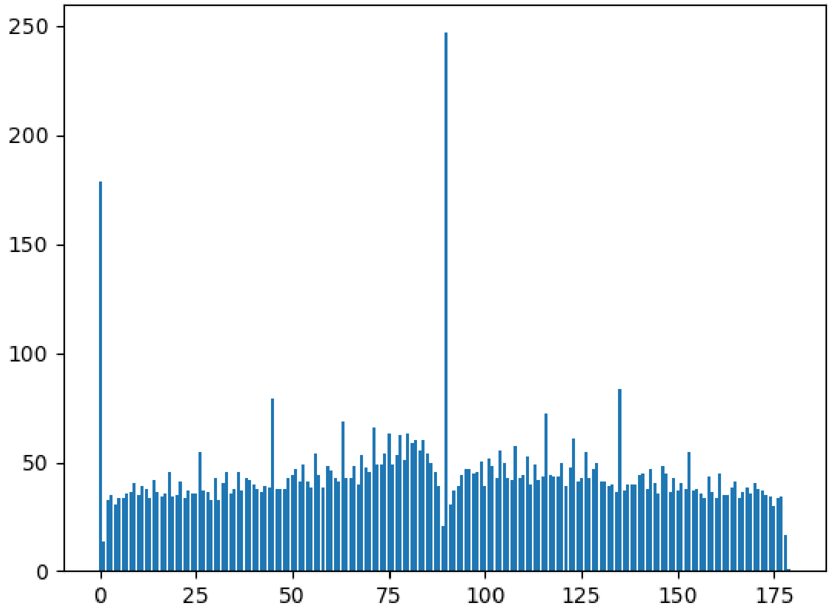

Figure S12: Histogram of oriented gradients analysis performed on AFM image in Fig.S13

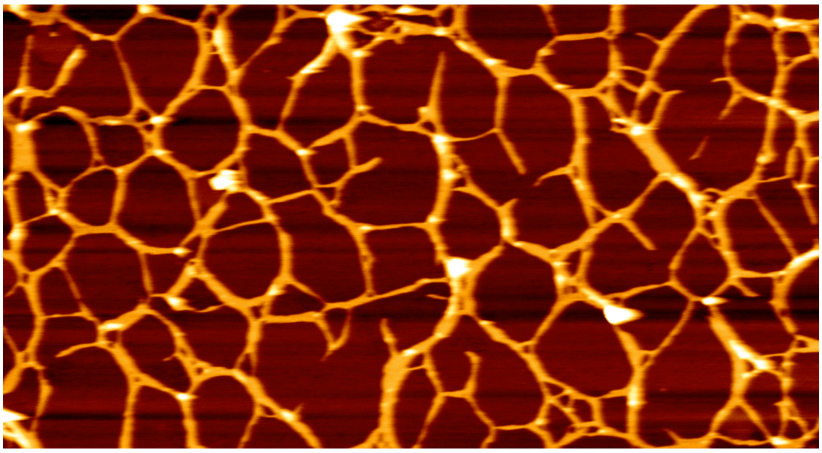

Figure S13: Fibril network of entangled $(1b_4)_n$ nanotubes obtained from chloroform solution on mica surface.

S7. Host-guest chemistry

The complexation of fullerenes by nanotubes $(1b_4)_n$ was assessed by using UV spectroscopy. The solid sample of the gels C_{60}@$(1b_4)_n$ and C_{70}@$(1b_4)_n$, prepared at 1:4 molar ratio, were diluted in toluene and UV spectra were collected (Fig. S14). Although only marginal shift of absorbance maximum (1-2 nm) was observed, the clear change in relative intensity of the absorption bands and the appearance of isobestic points was noted using both, C_{60} and C_{70}. The very small change in absorbance maximum is in line with the results from our previous studies on fullerene complexation with structurally related tetrameric supramolecular host.[2]

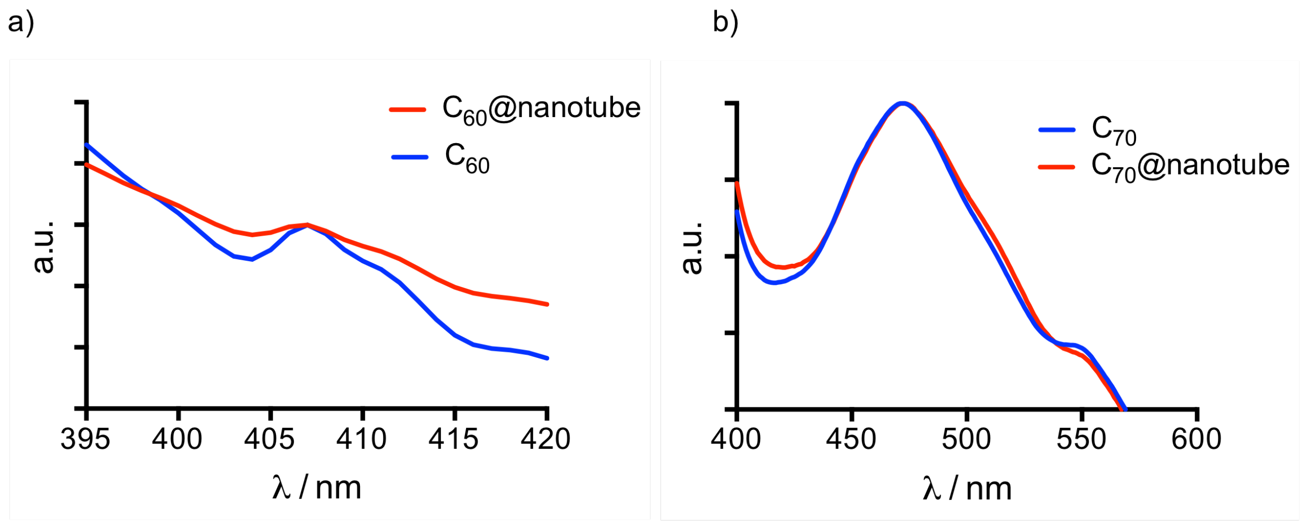

Figure S14: Normalized UV spectra of C_{60}@$(1b_4)_n$ (a) and C_{70}@$(1b_4)_n$ (b) in toluene.

S8. Molecular modelling

The geometry of tetramer $\mathbf{2}_4$ was first calculated at semi-empirical level of theory (PM3, as implemented in Spartan 10) (Fig. S15).[18] The optimized structure of tetramer $\mathbf{2}_4$ was then used to construct an octameric fragment of the nanotube $(\mathbf{2}_4)_n$. Due to the very large number of atoms, the resulting structure was optimized using molecular mechanics. The results of the molecular modelling show that despite the large size of solubilizing chains, a stable tubular polymer, potentially benefiting from favourable π- π interactions, can form (Fig. S16).

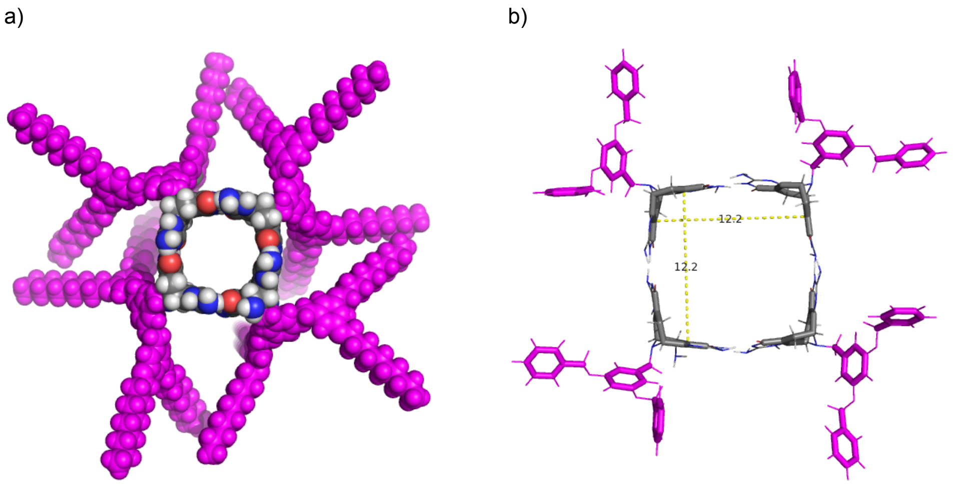

Figure S15: a) Top-view of tetramer $\mathbf{2}_4$. b) Minimalistic representation of tetramer $\mathbf{2}_4$ without decyl chains with the diameter indicated (in Å). The solubilizing chains are labelled in magenta for clarity.

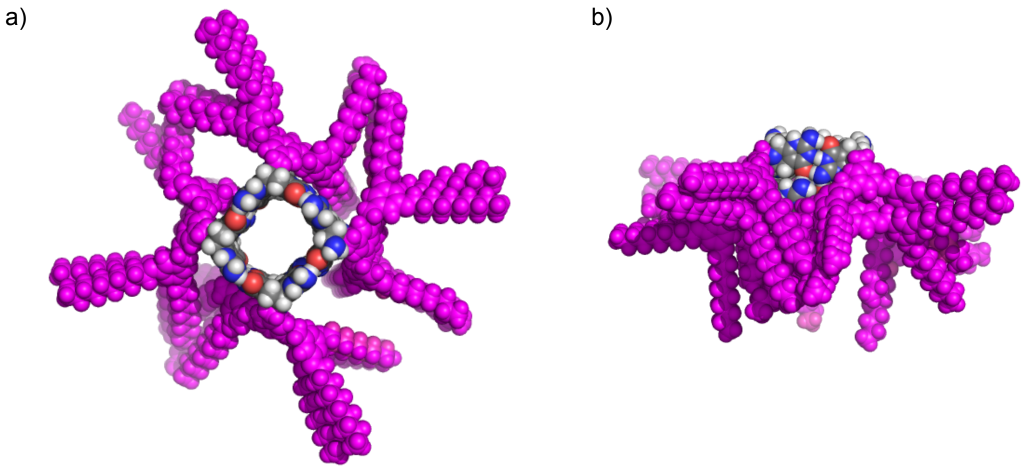

Figure S16: a) Top-view of octamer $(\mathbf{2}_4)_2$. b) Side-view of octamer $(\mathbf{2}_4)_2$.

S9. Copies of NMR spectra

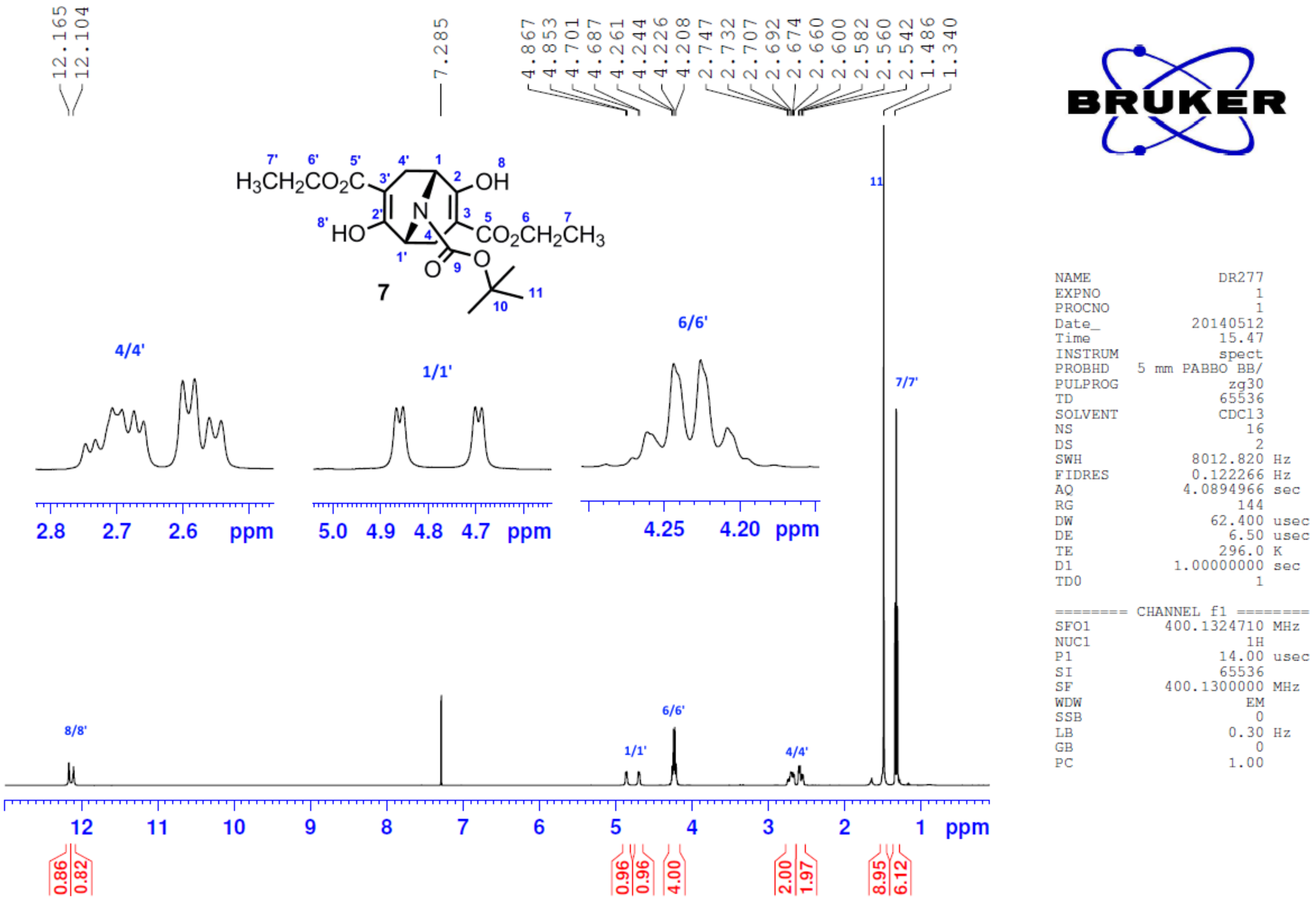

7

Figure S17: ^{1}H NMR spectrum of **7**.

return to Synthesis

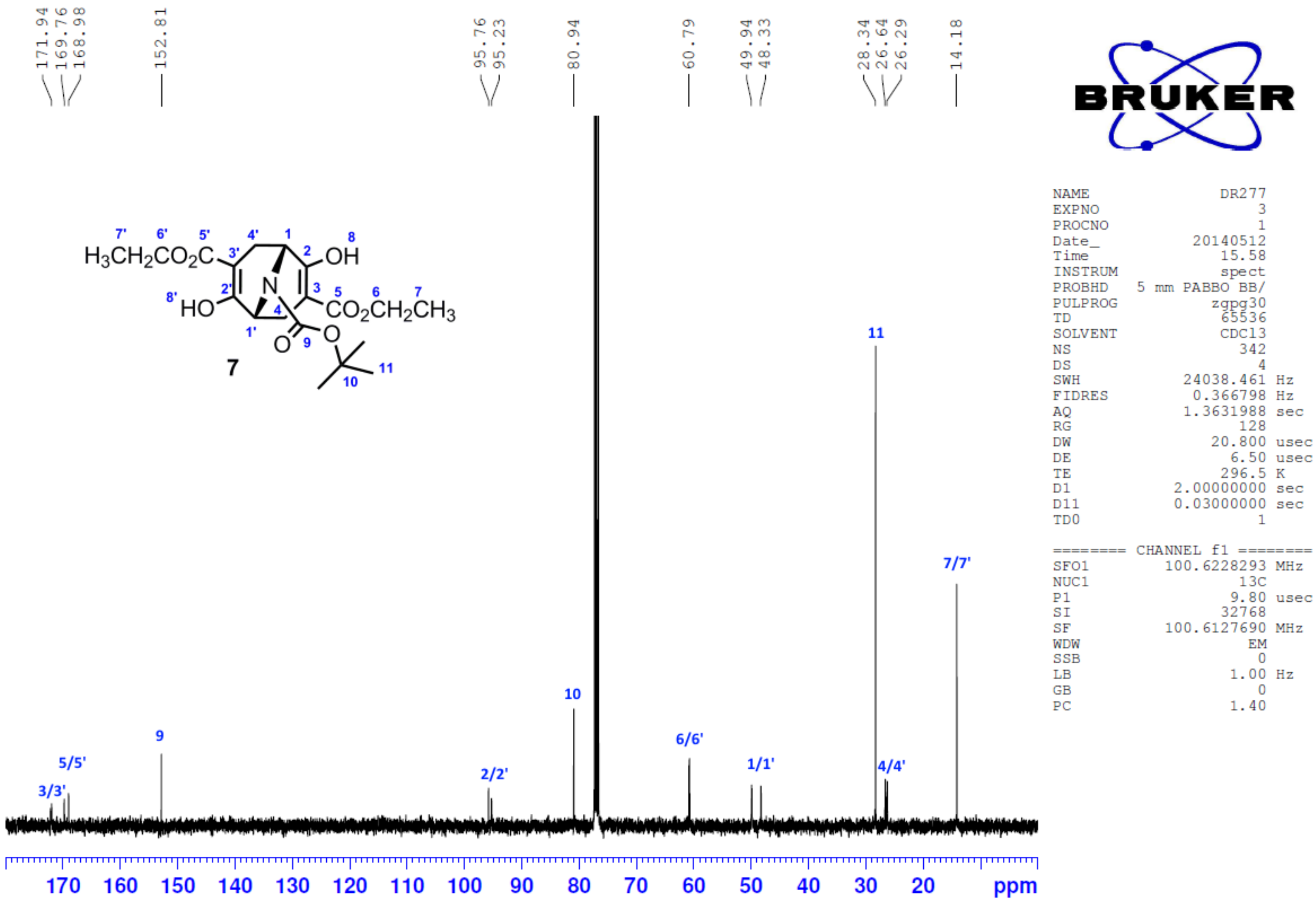

Figure S18: ^{13}C NMR spectrum of **7**.

return to Synthesis

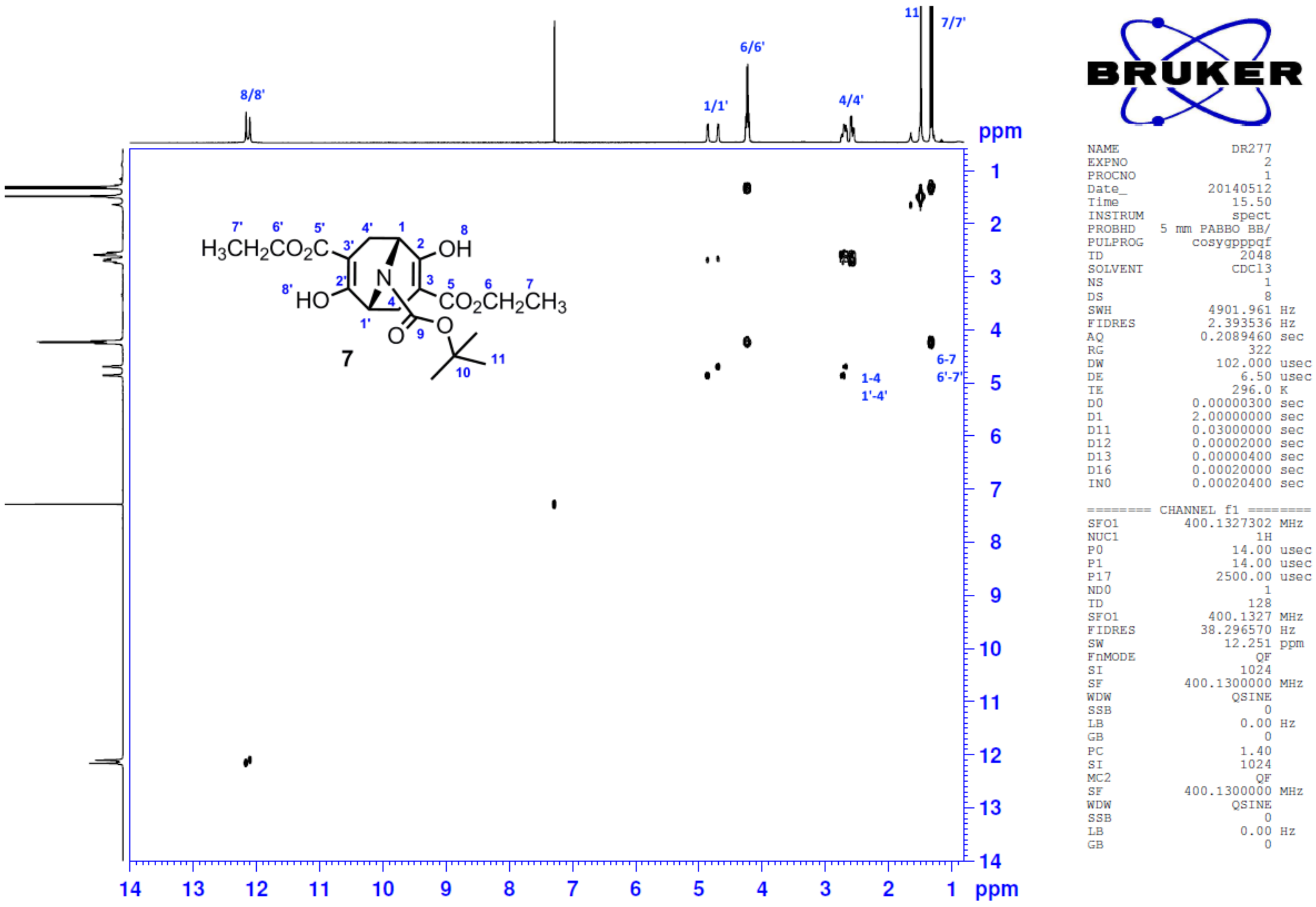

Figure S19: COSY NMR spectrum of **7**.

return to Synthesis

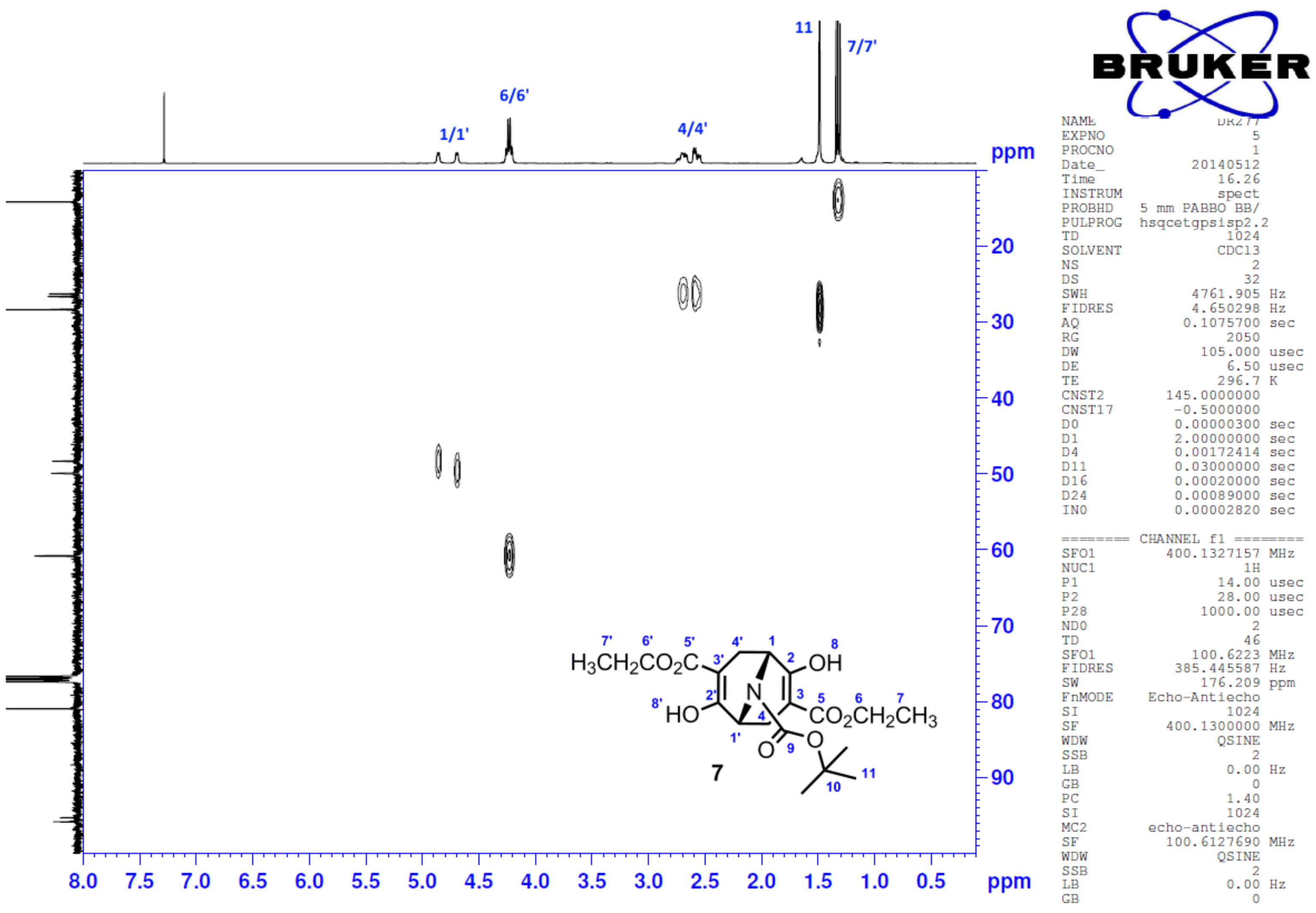

Figure S20: HSQC NMR spectrum of **7**.

return to Synthesis

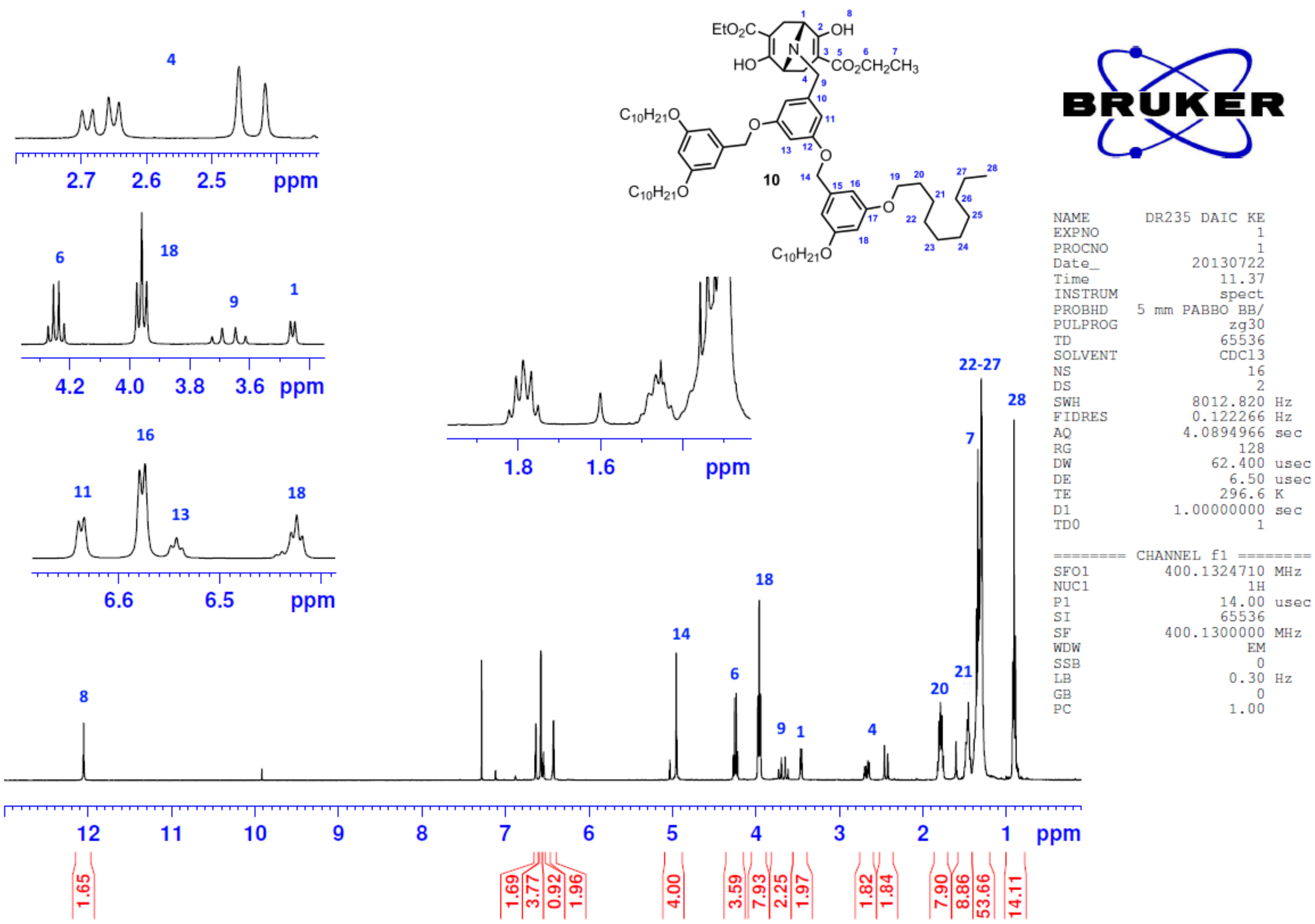

Figure S21: ^{1}H NMR spectrum of **10**.

return to Synthesis

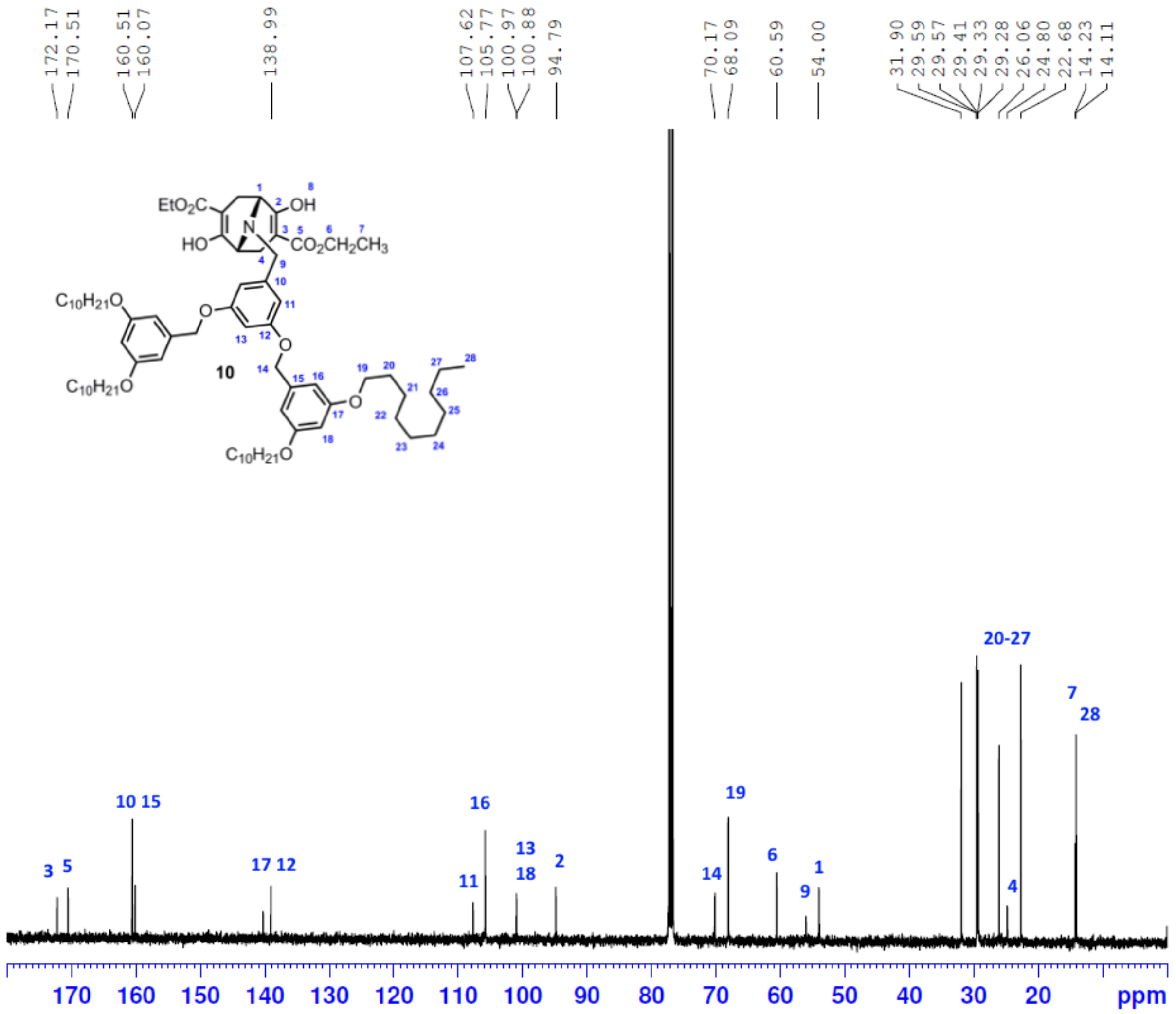

NAME	DR235 DAIC KE	
EXPNO	2	
PROCNO	1	
Date_	20130722	
Time	11.45	
INSTRUM	spect	
PROBHD	5 mm PABBO BB/	
PULPROG	zgpg30	
TD	65536	
SOLVENT	CDCl3	
NS	1024	
DS	4	
SWH	24038.461	Hz
FIDRES	0.366798	Hz
AQ	1.3631988	sec
RG	144	
DW	20.800	usec
DE	6.50	usec
TE	297.5	K
D1	2.00000000	sec
D11	0.03000000	sec
TD0	1	
======== CHANNEL f1 ========		
SFO1	100.6228293	MHz
NUC1	13C	
P1	9.80	usec
SI	32768	
SF	100.6127690	MHz
WDW	EM	
SSB	0	
LB	1.00	Hz
GB	0	
PC	1.40	

Figure S22: ^{13}C NMR spectrum of **10**.

return to Synthesis

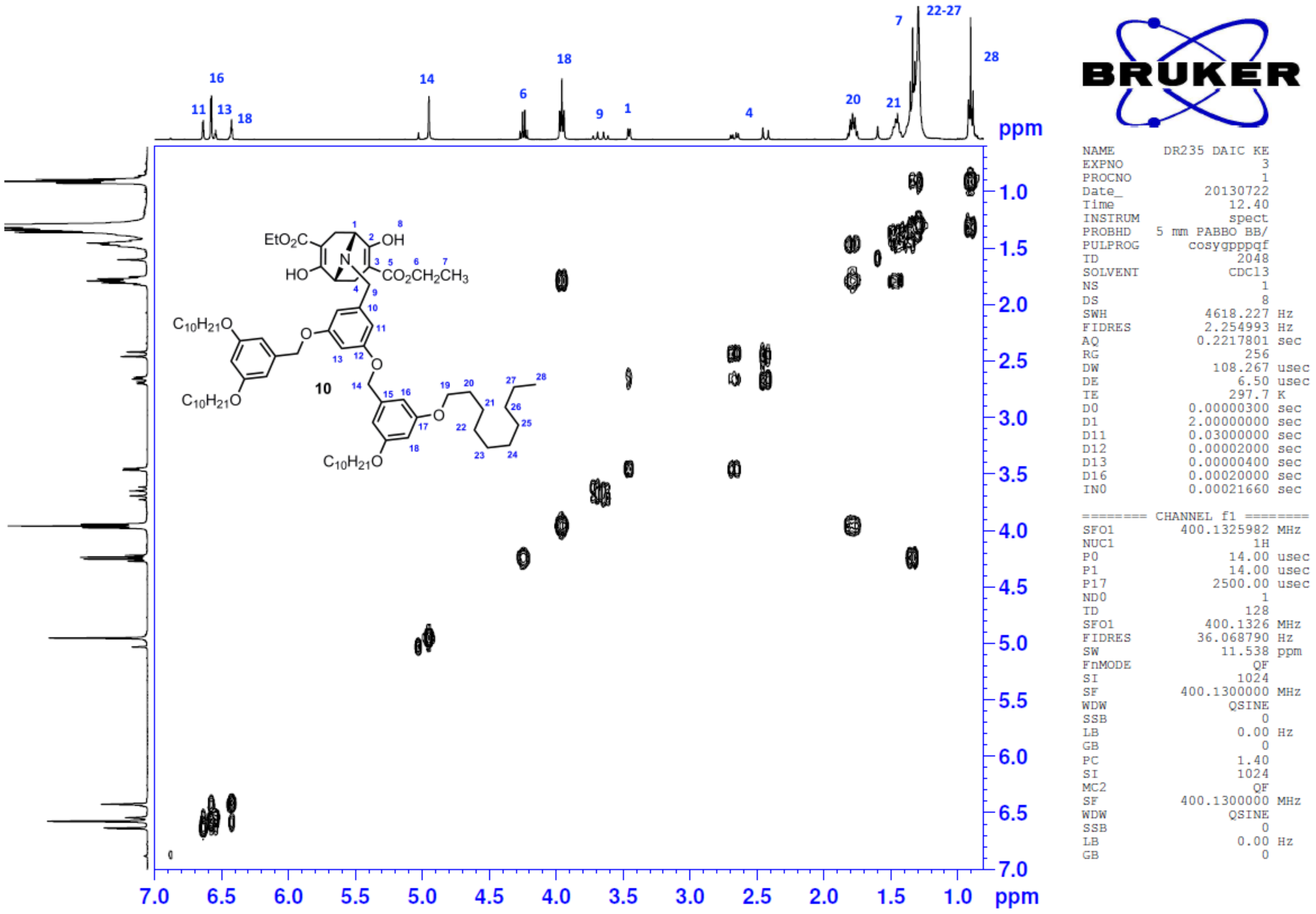

Figure S23: COSY NMR spectrum of **10**.

return to Synthesis

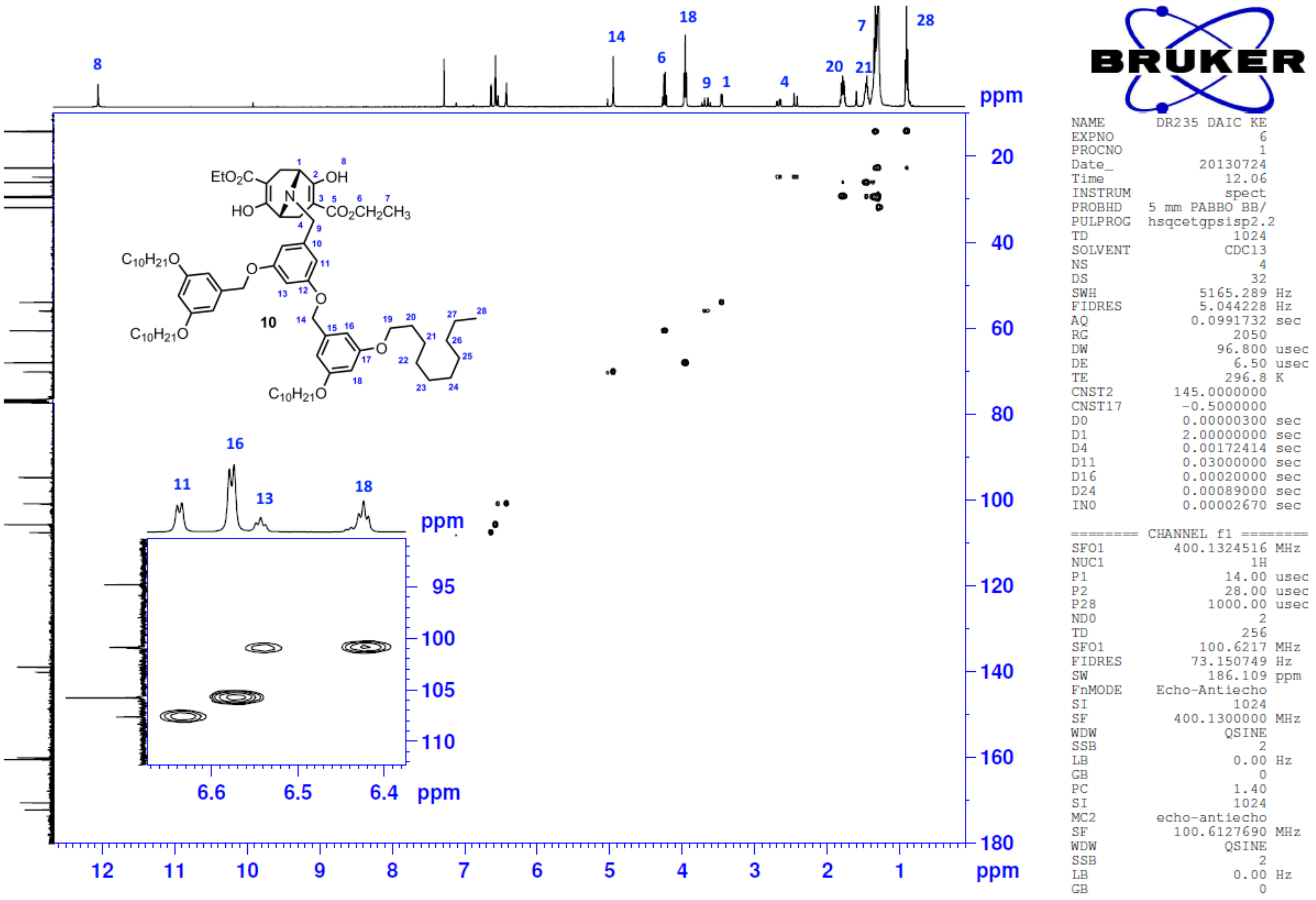

Figure S24: HSQC NMR spectrum of **10**.

return to Synthesis

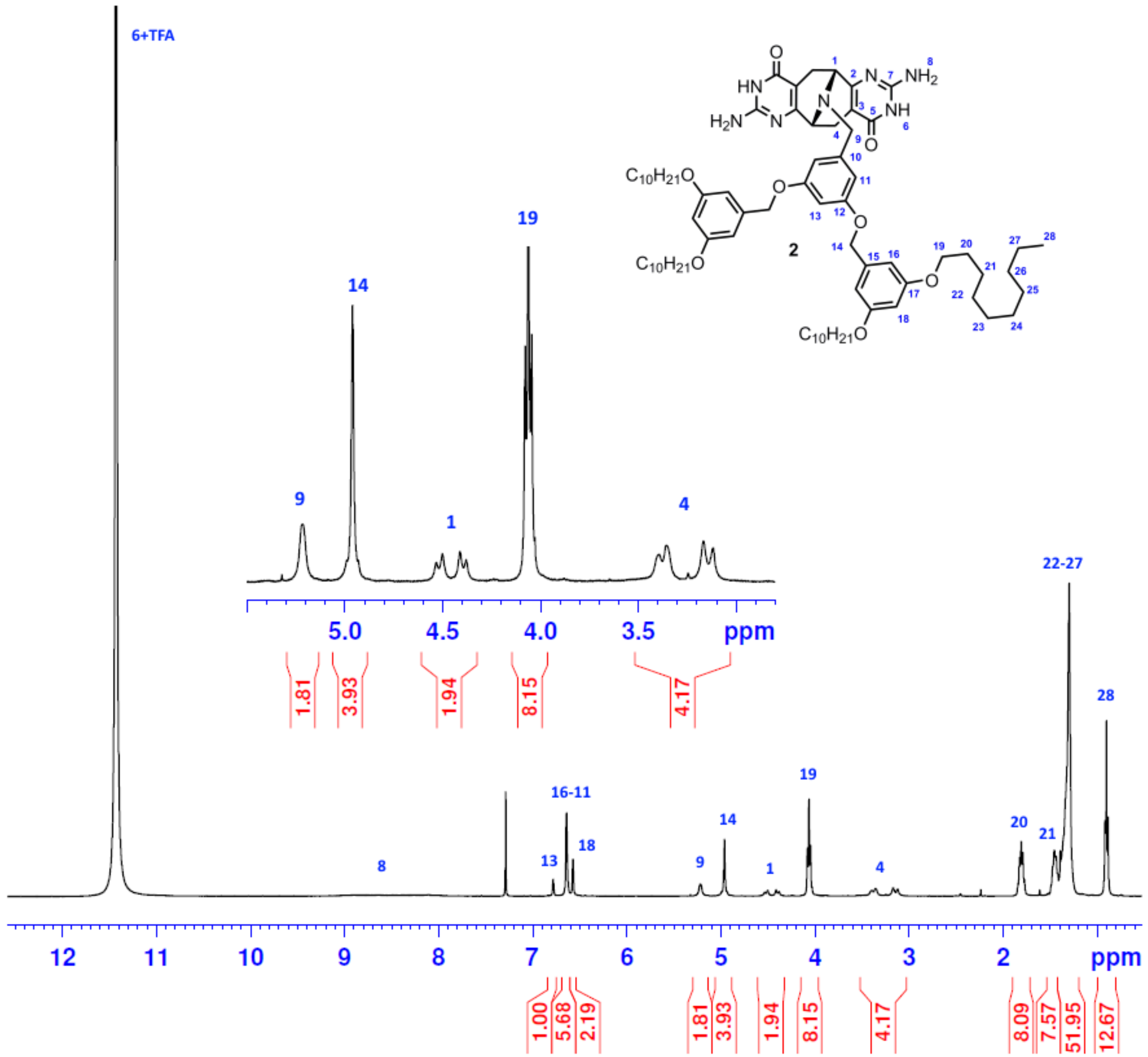

Figure S25: ^{1}H NMR spectrum of **2**.

return to Synthesis

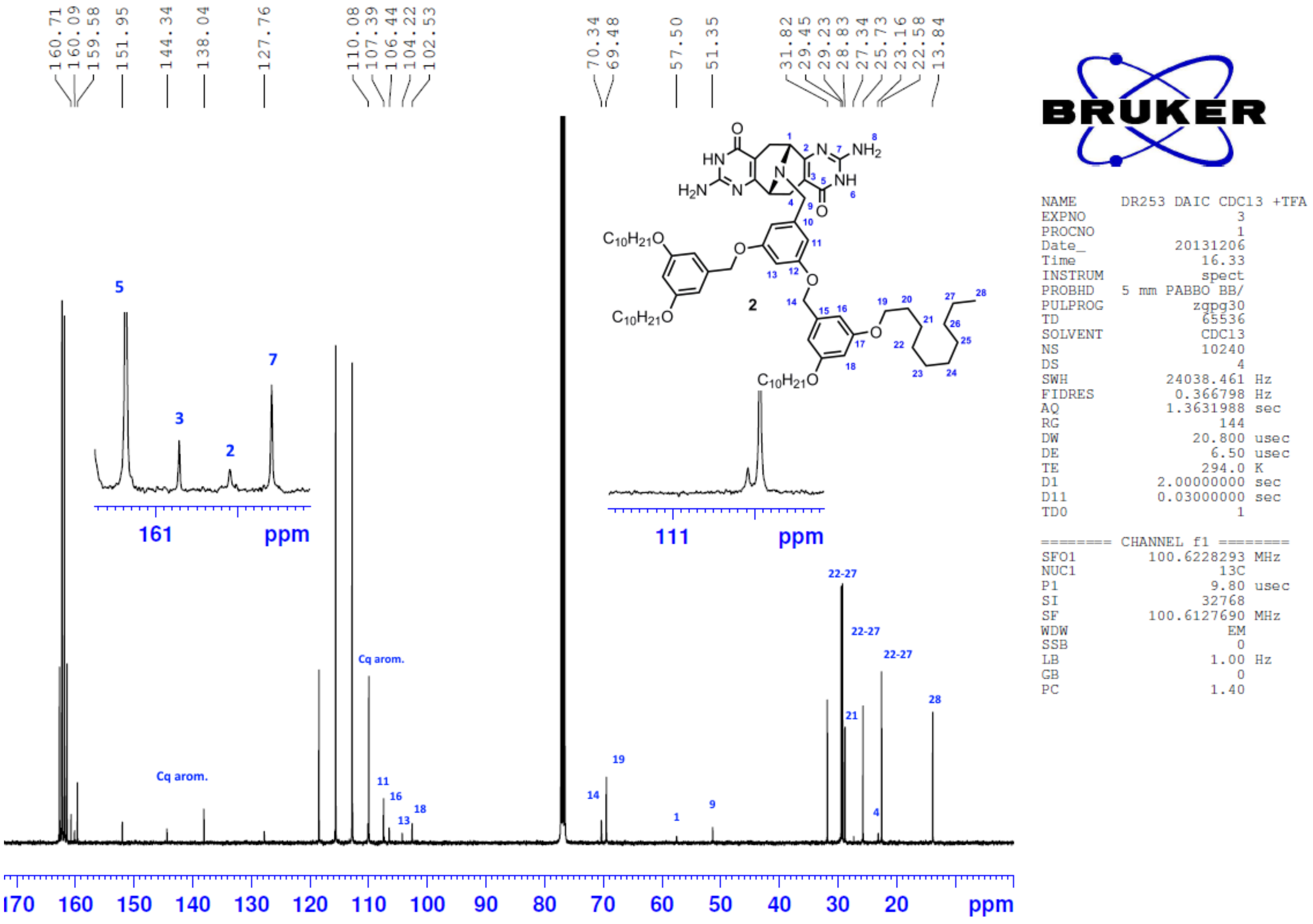

Figure S26: ^{13}C NMR spectrum of **2**.

return to Synthesis

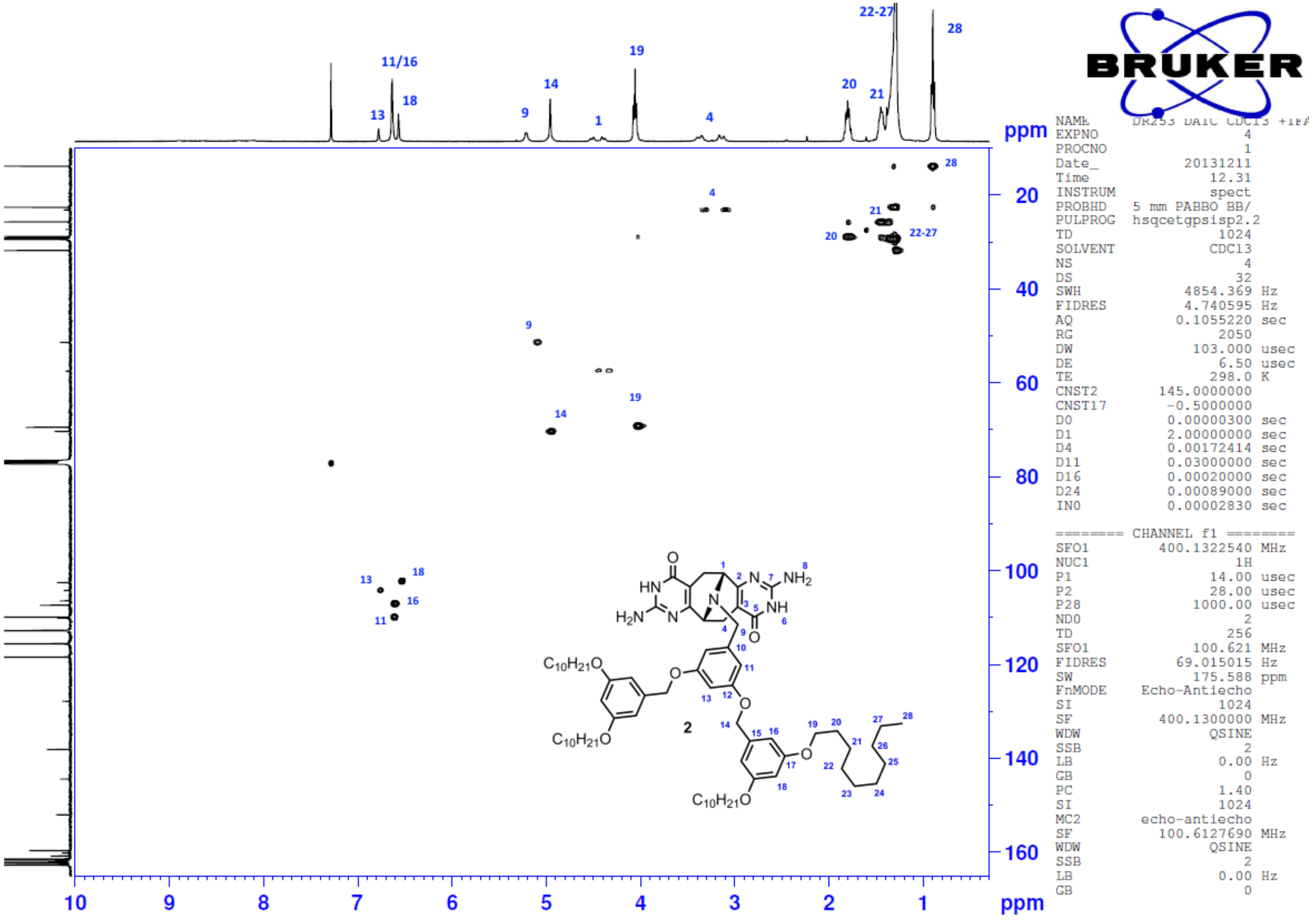

Figure S27: HSQC NMR spectrum of **2**.

return to Synthesis

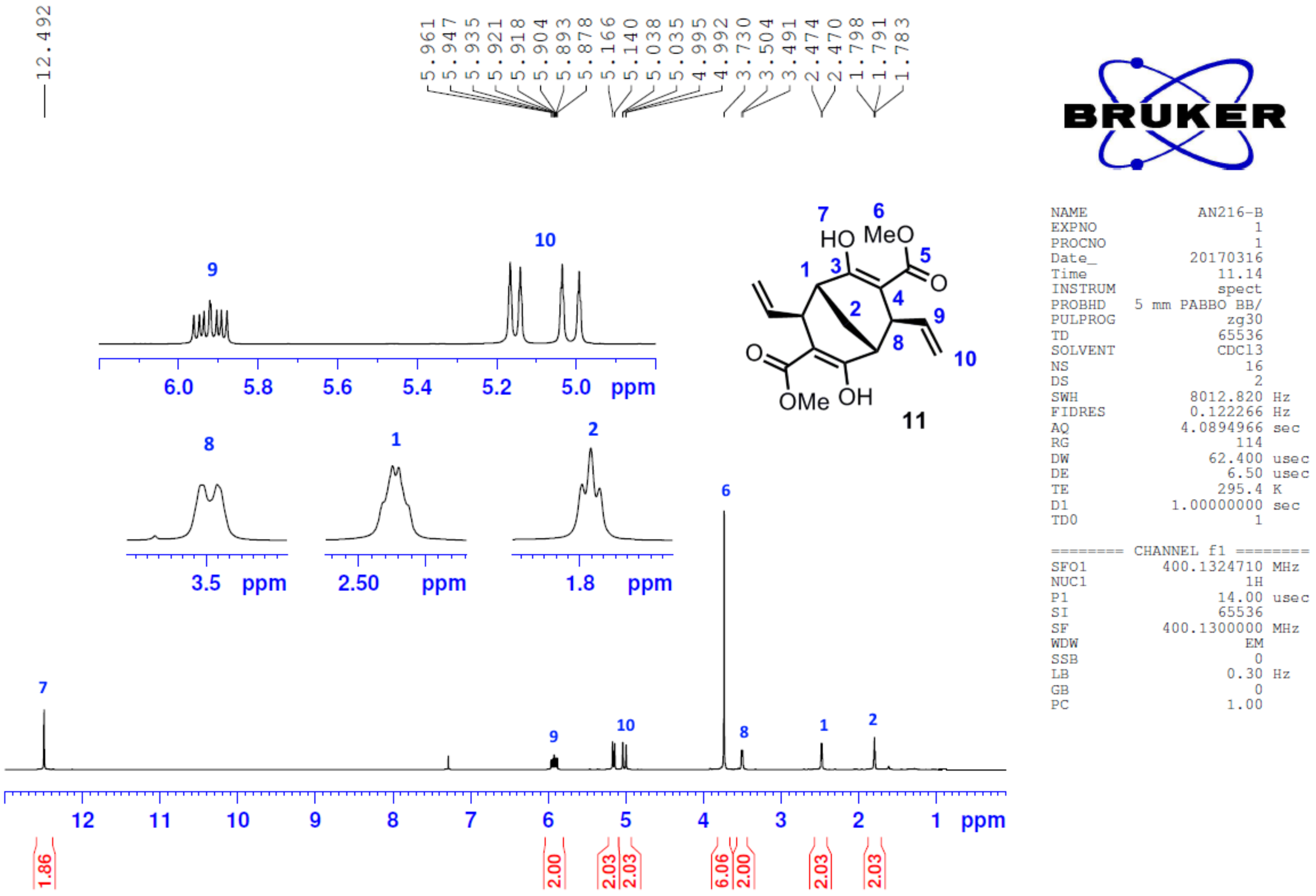

Figure S28: ^{1}H NMR spectrum of **11**.

return to Synthesis

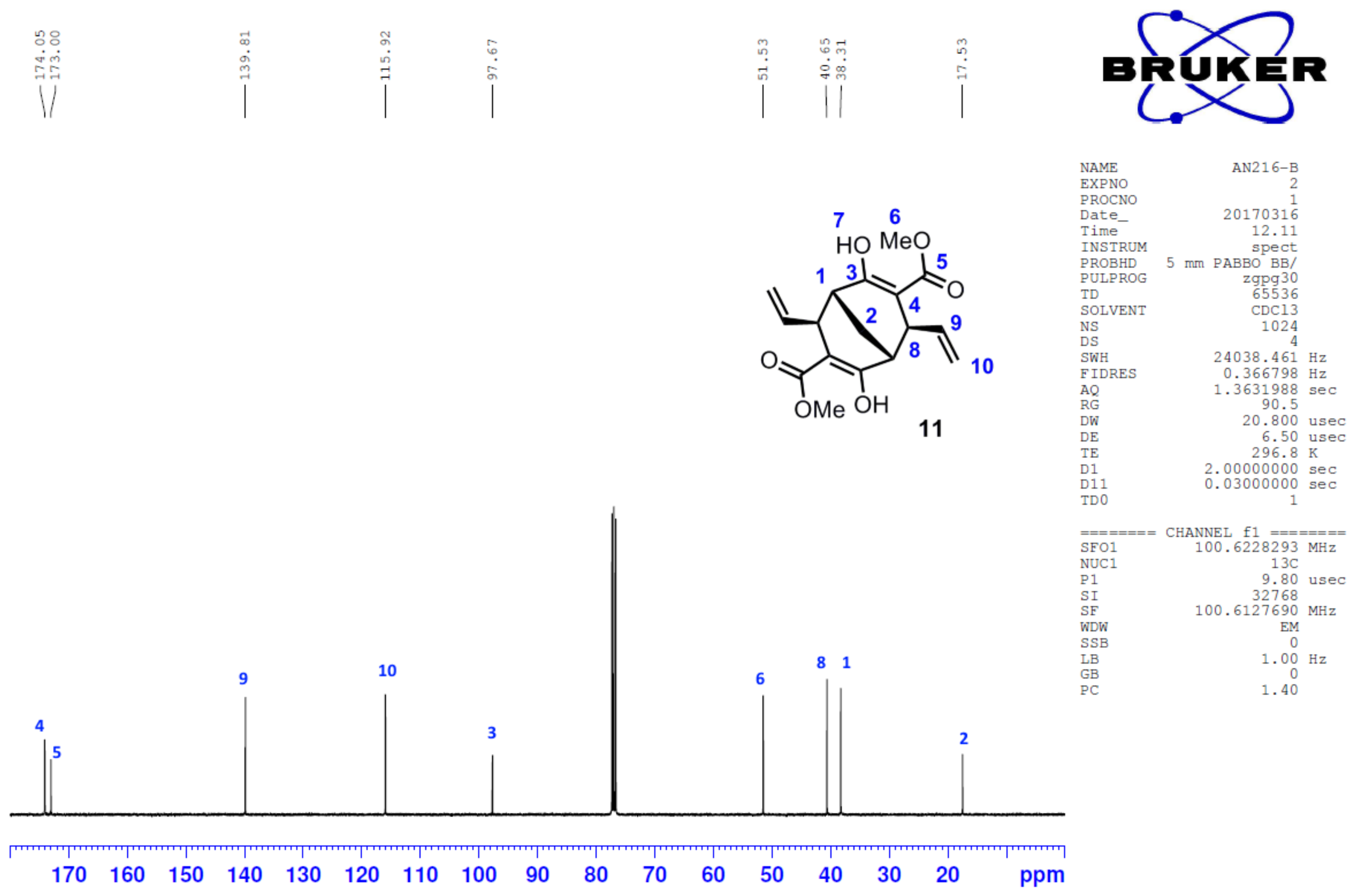

Figure S29: ^{13}C NMR spectrum of **11**.

return to Synthesis

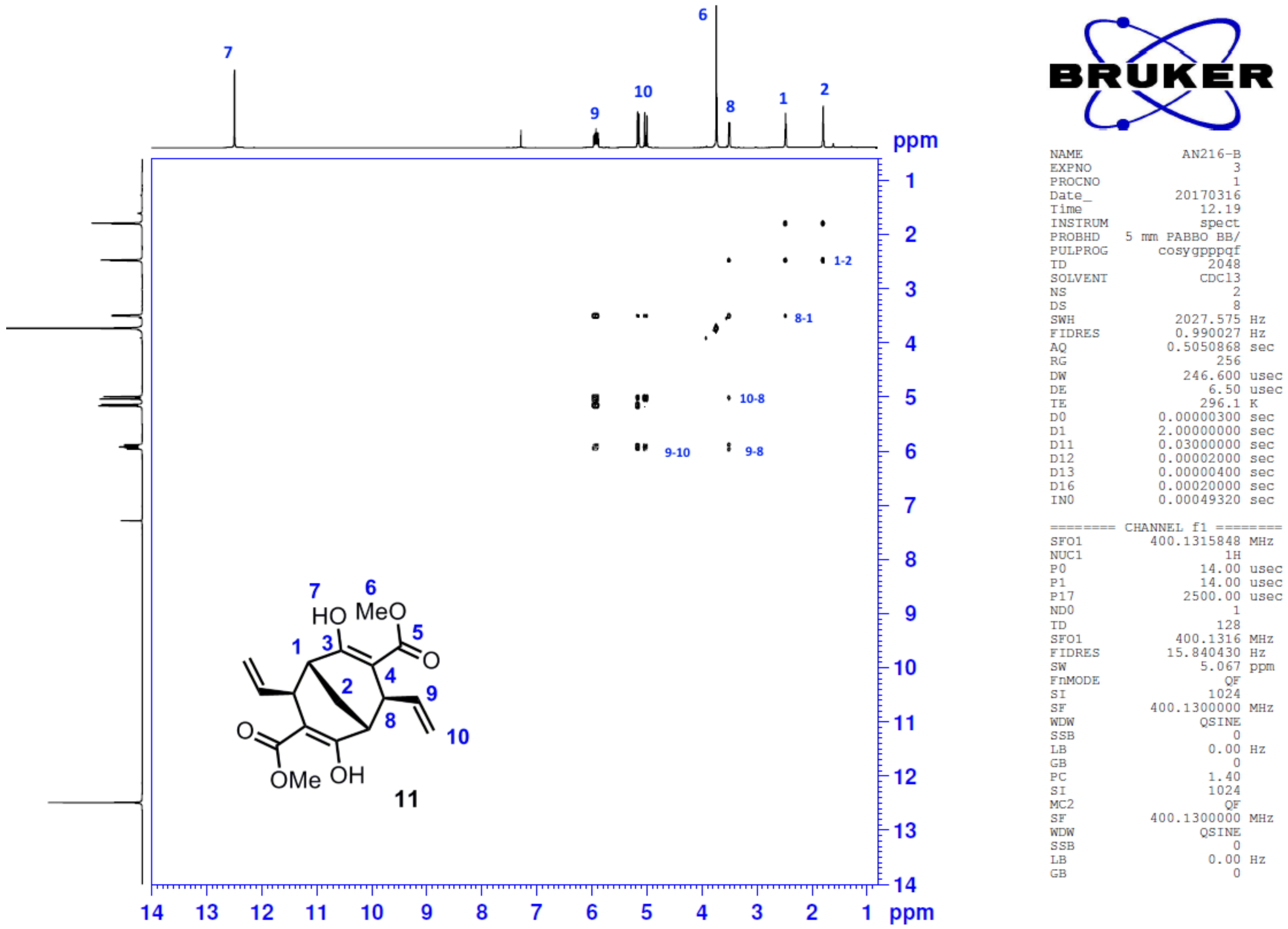

Figure S30: COSY NMR spectrum of **11**.

return to Synthesis

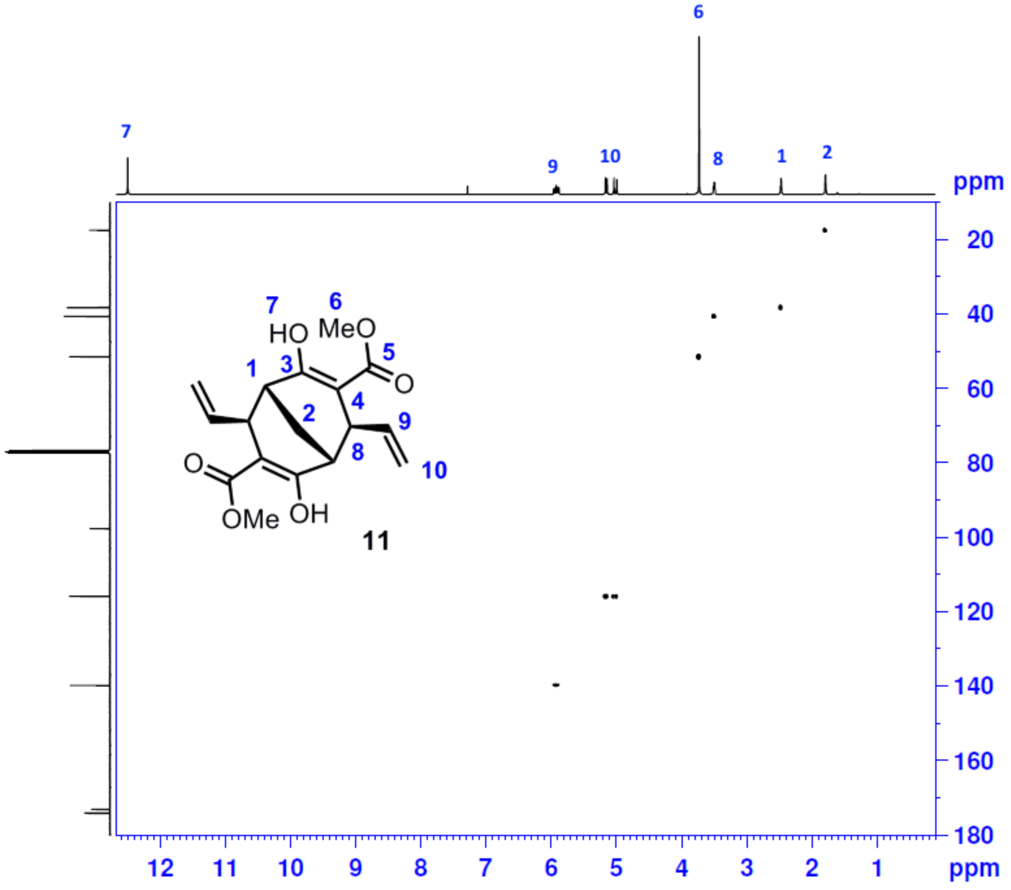

NAME	AN216-B	
EXPNO	4	
PROCNO	1	
Date_	20170316	
Time	12.31	
INSTRUM	spect	
PROBHD	5 mm PABBO BB/	
PULPROG	hsqcetgpsi2	
TD	1024	
SOLVENT	CDCl3	
NS	4	
DS	16	
SWH	2027.575	Hz
FIDRES	1.980054	Hz
AQ	0.2525684	sec
RG	2050	
DW	246.600	usec
DE	6.50	usec
TE	295.6	K
CNST2	145.0000000	
D0	0.00000300	sec
D1	1.50000000	sec
D4	0.00172414	sec
D11	0.03000000	sec
D16	0.00020000	sec
D24	0.00086207	sec
IN0	0.00003310	sec
ZGOPTNS		
======== CHANNEL f1 ========		
SFO1	400.1315848	MHz
NUC1	1H	
P1	14.00	usec
P2	28.00	usec
P28	1000.00	usec
ND0	2	
TD	256	
SFO1	100.6203	MHz
FIDRES	59.006798	Hz
SW	150.126	ppm
FnMODE	Echo-Antiecho	
SI	1024	
SF	400.1300000	MHz
WDW	QSINE	
SSB	2	
LB	0.00	Hz
GB	0	
PC	1.40	
SI	1024	
MC2	echo-antiecho	
SF	100.6127690	MHz
WDW	QSINE	
SSB	2	
LB	0.00	Hz
GB	0	

Figure S31: HSQC NMR spectrum of **11**.

return to Synthesis

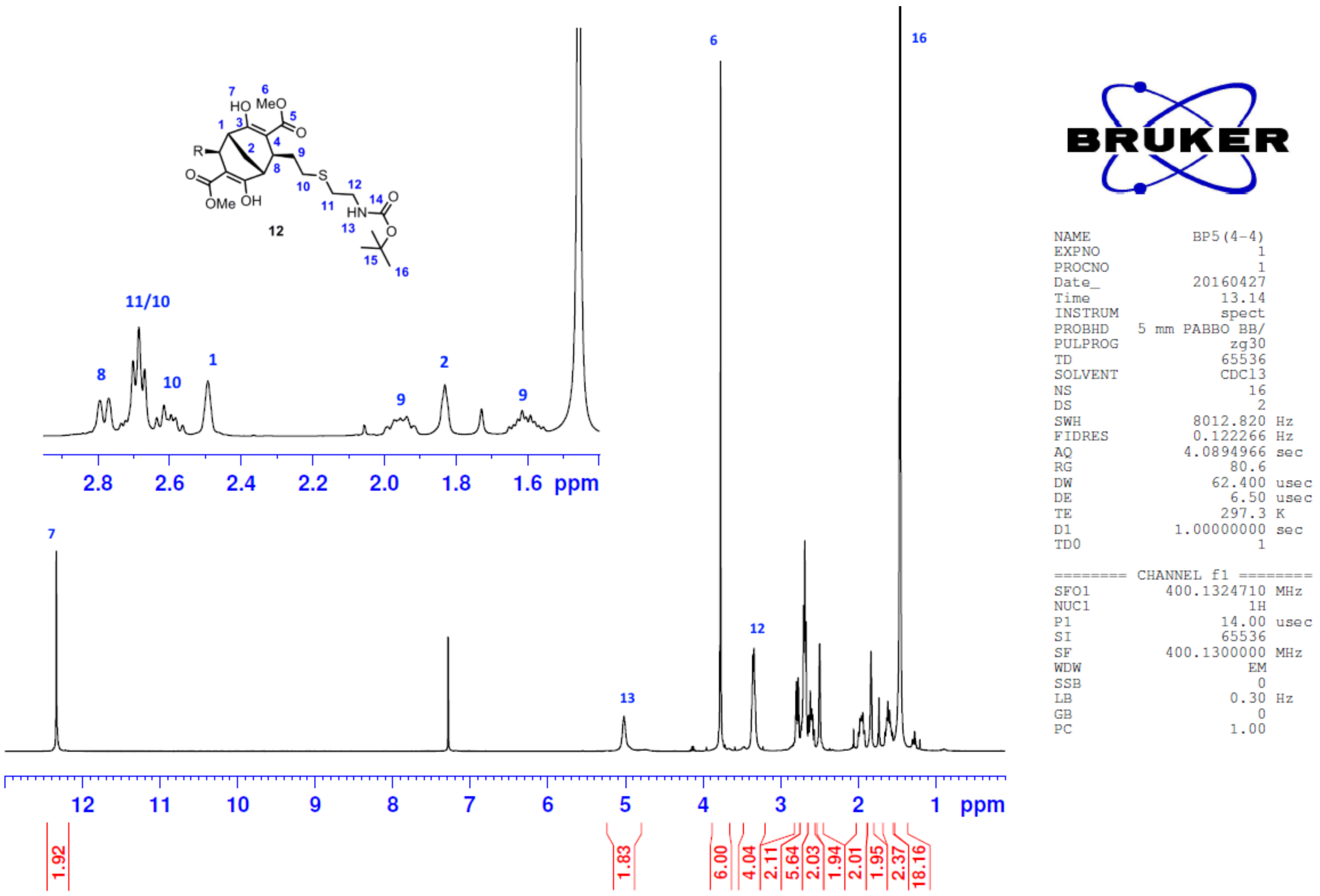

Figure S32: ^{1}H NMR spectrum of **12**.

return to Synthesis

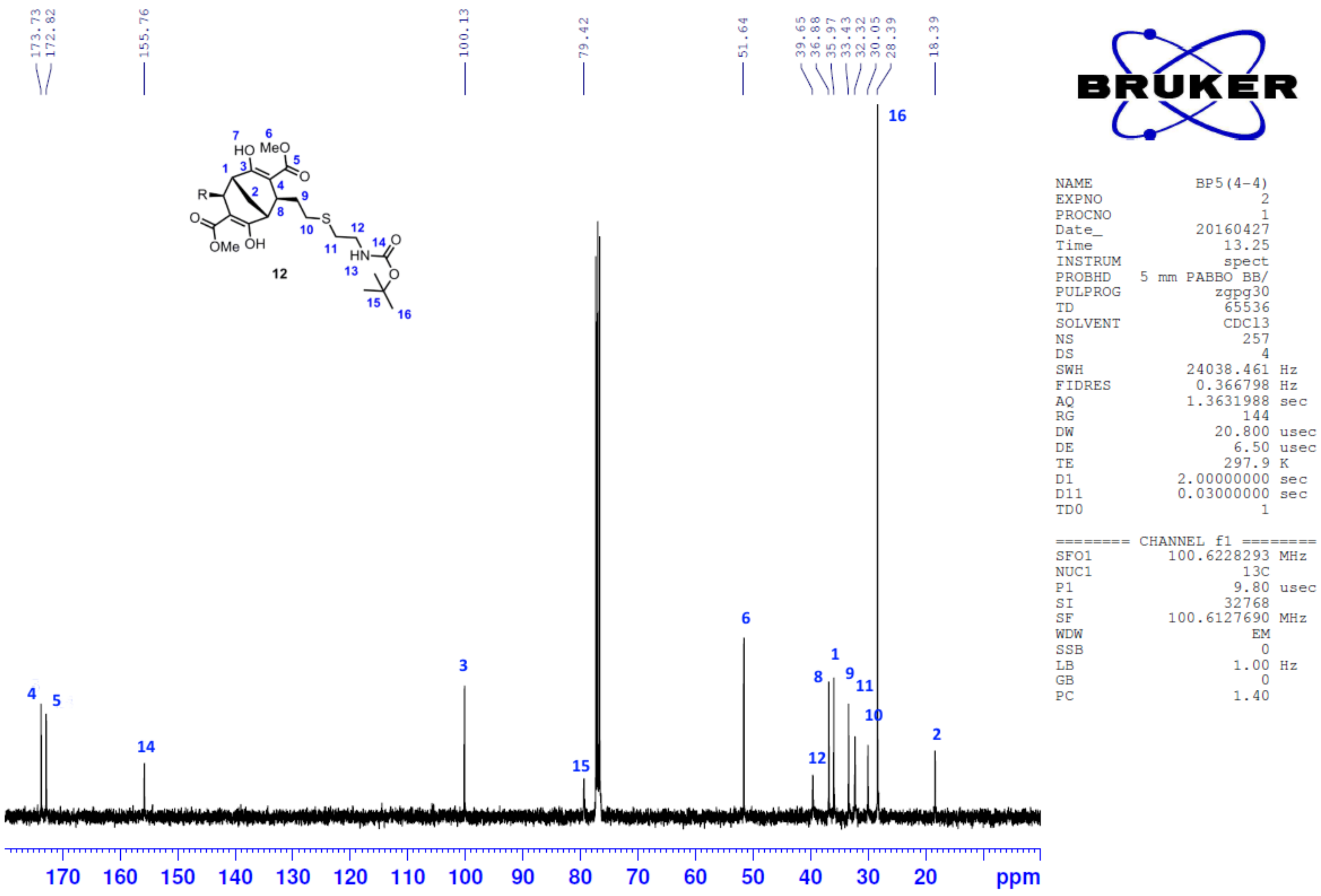

Figure S33: ^{13}C NMR spectrum of **12**.

return to Synthesis

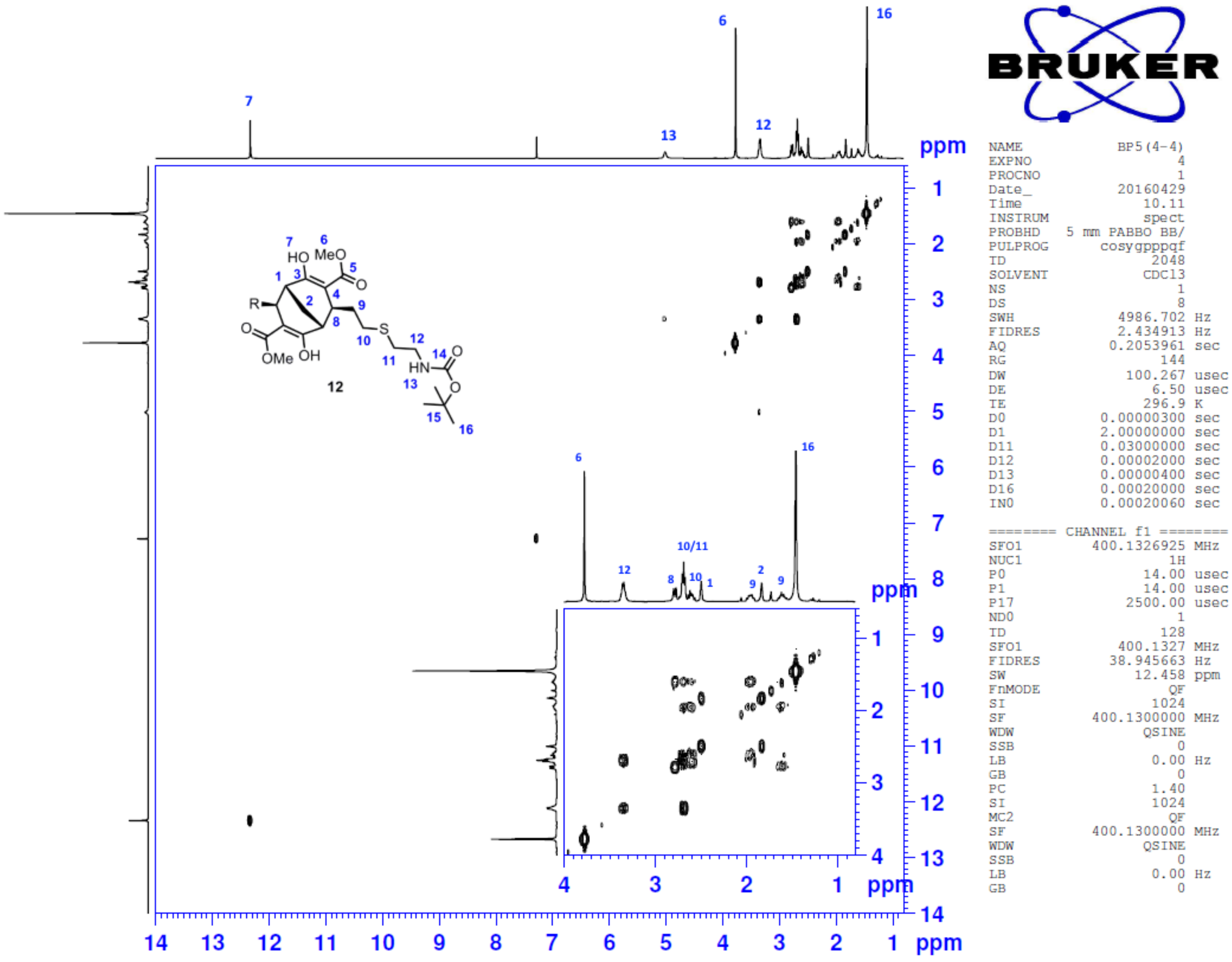

Figure S34: COSY NMR spectrum of **12**.

return to Synthesis

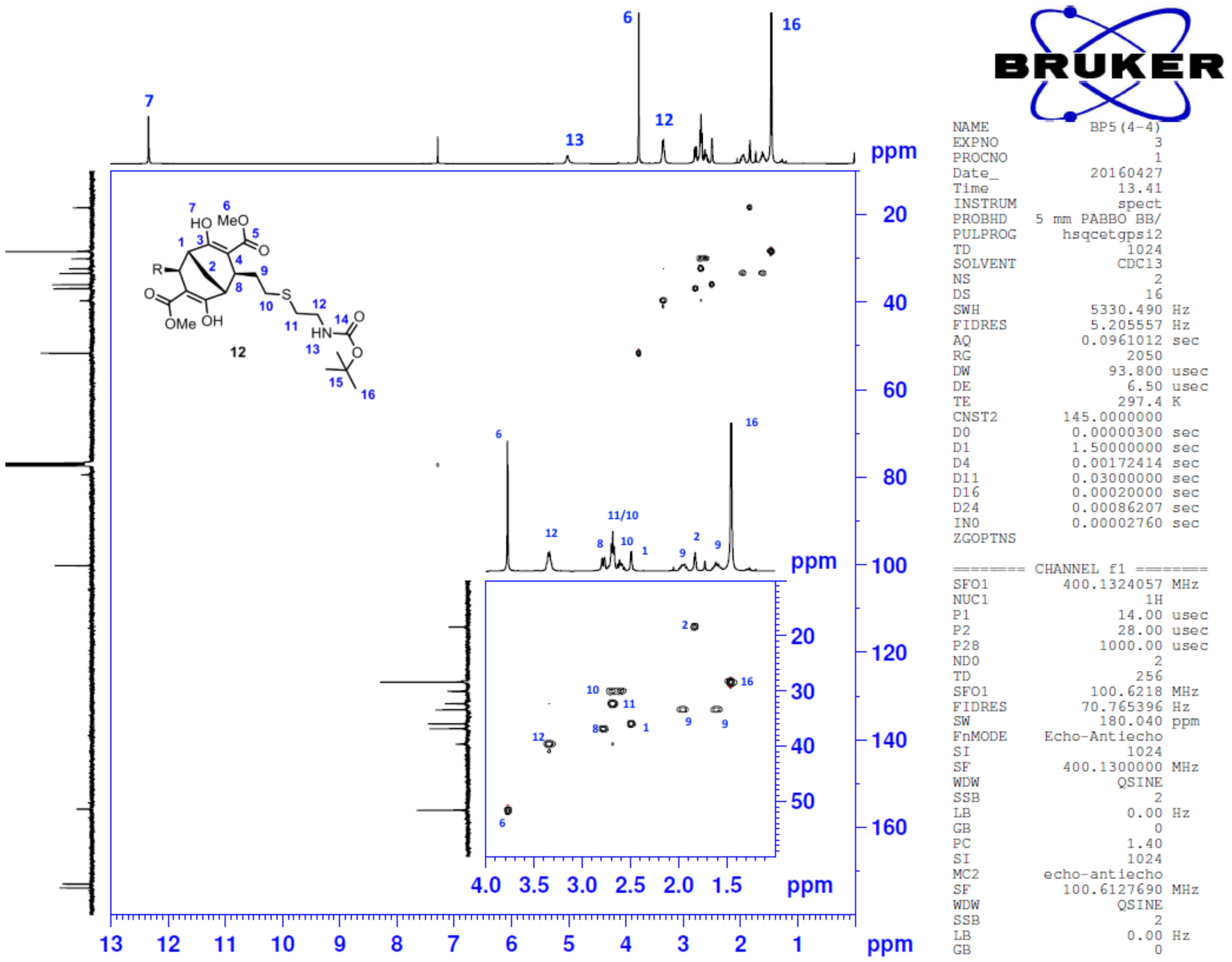

Figure S35: HSQC NMR spectrum of **12**.

return to Synthesis

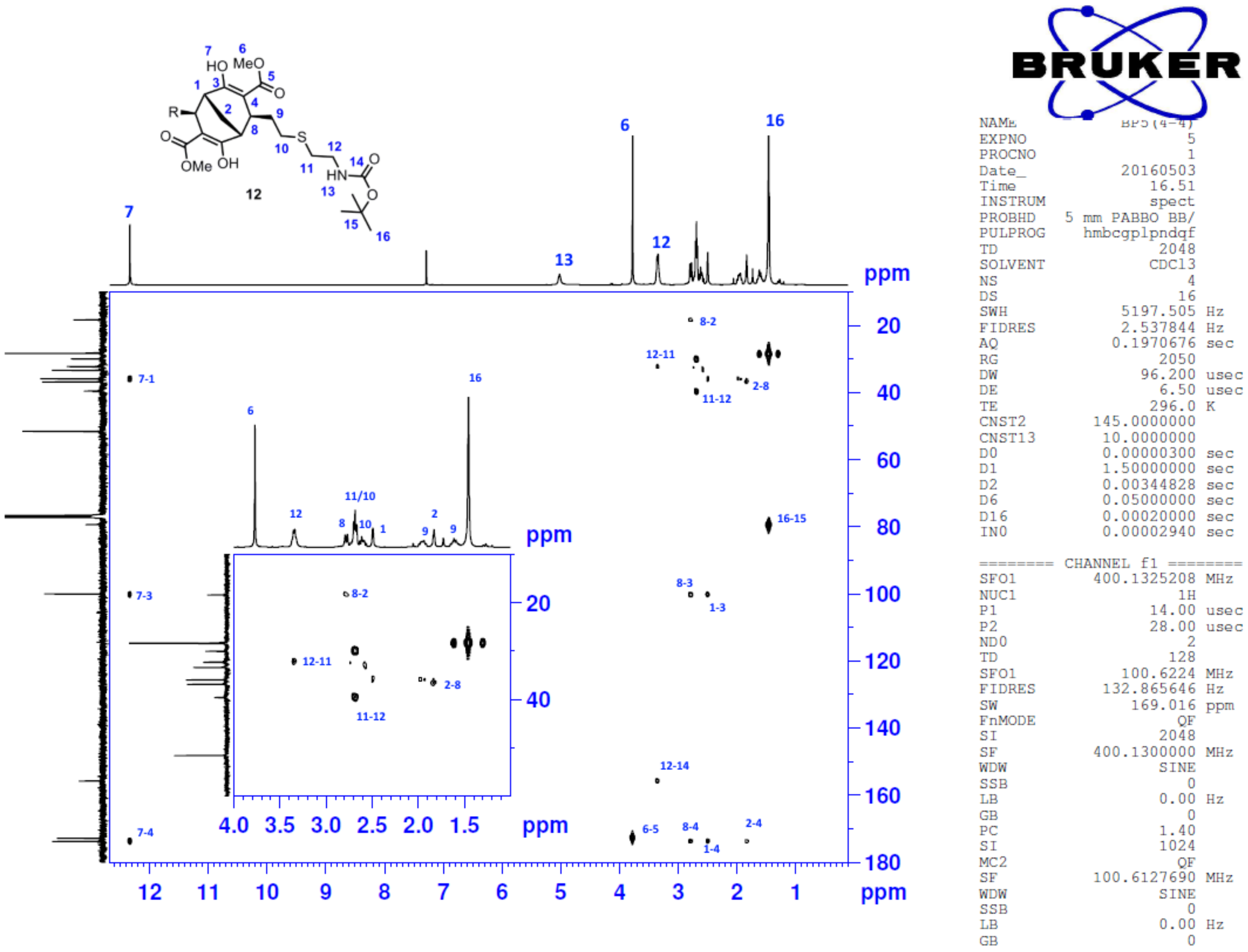

Figure S36: HMBC NMR spectrum of **12**.

return to Synthesis

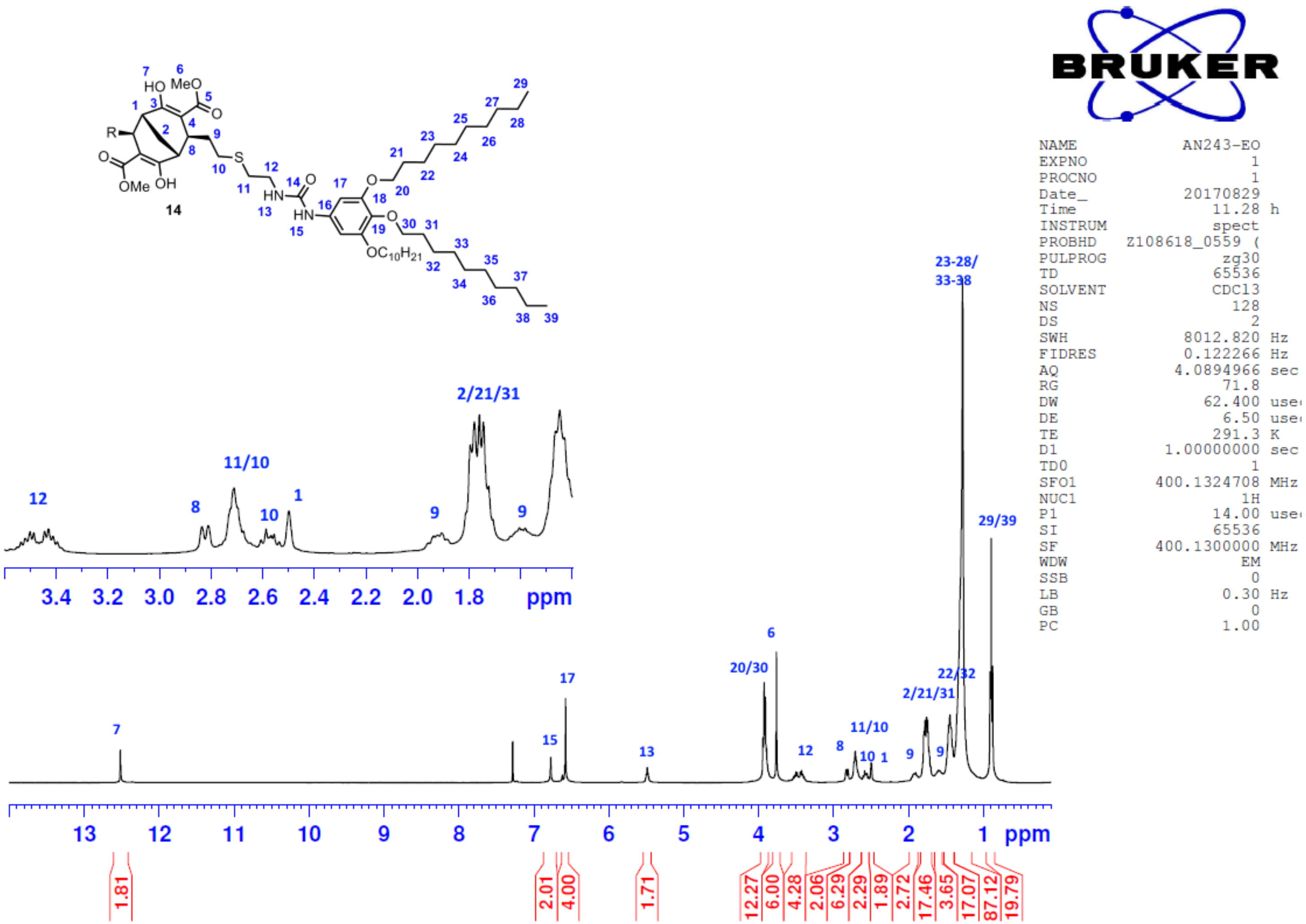

Figure S37: ^{1}H NMR spectrum of **14**.

return to Synthesis

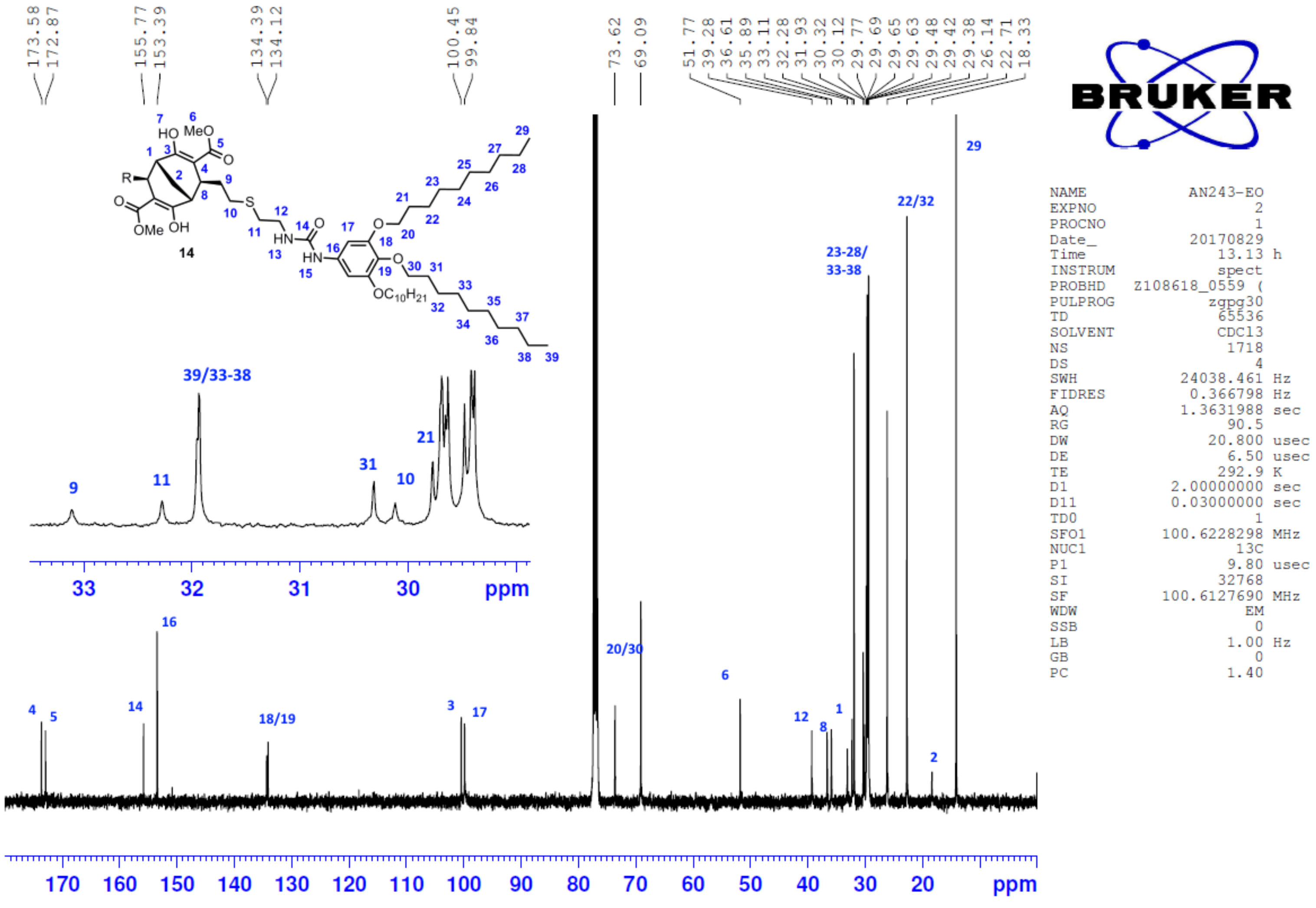

Figure S38: ^{13}C NMR spectrum of **14**.

return to Synthesis

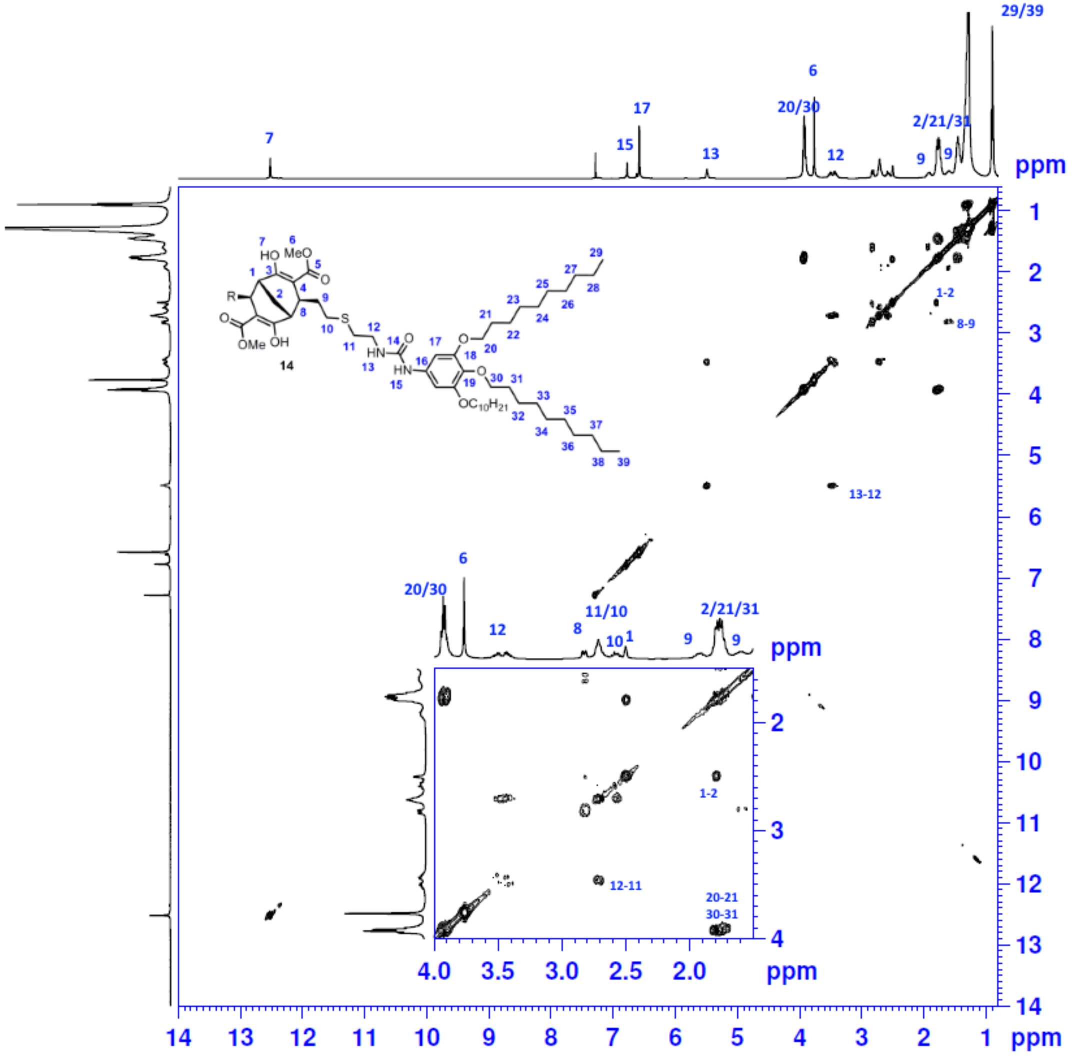

NAME	AN243-EO
EXPNO	3
PROCNO	1
Date_	20170831
Time	10.59 h
INSTRUM	spect
PROBHD	Z108618_0559 (
PULPROG	cosygpppqf
TD	2048
SOLVENT	CDCl3
NS	2
DS	16
SWH	5136.986 Hz
FIDRES	2.508294 Hz
AQ	0.1993887 sec
RG	101
DW	97.333 usec
DE	6.50 usec
TE	291.6 K
D0	0.00000300 sec
D1	2.00000000 sec
D11	0.03000000 sec
D12	0.00002000 sec
D13	0.00000400 sec
D16	0.00020000 sec
IN0	0.00019460 sec
ND0	1
TD	256
SFO1	400.1325 MHz
FIDRES	20.073227 Hz
SW	12.843 ppm
FnMODE	QF
SI	1024
SF	400.1300000 MHz
WDW	QSINE
SSB	0
LB	0.00 Hz
GB	0
PC	1.40
SI	1024
MC2	QF
SF	400.1300000 MHz
WDW	QSINE
SSB	0
LB	0.00 Hz
GB	0

Figure S39: COSY NMR spectrum of **14**.

return to Synthesis

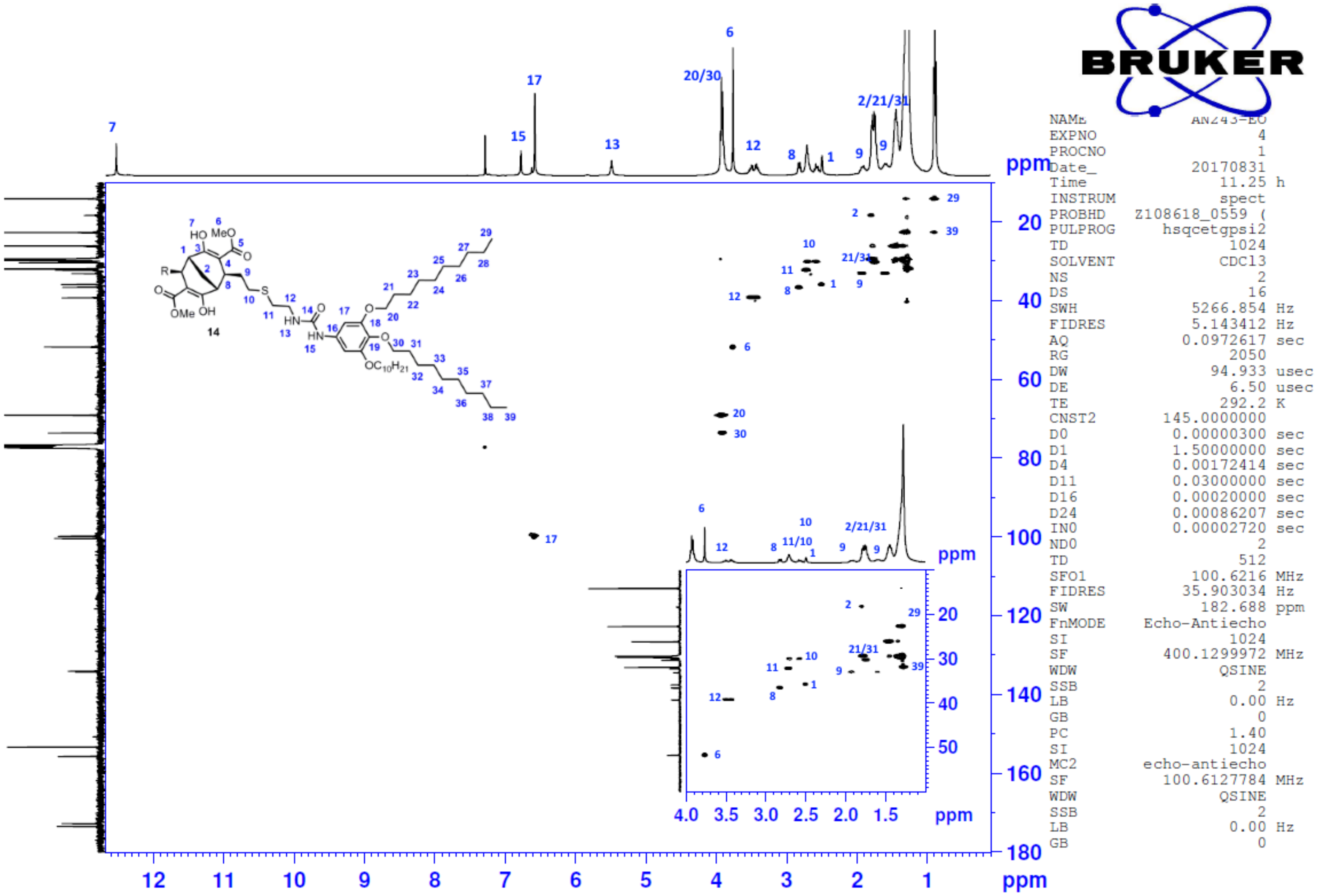

Figure S40: HSQC NMR spectrum of **14**.

return to Synthesis

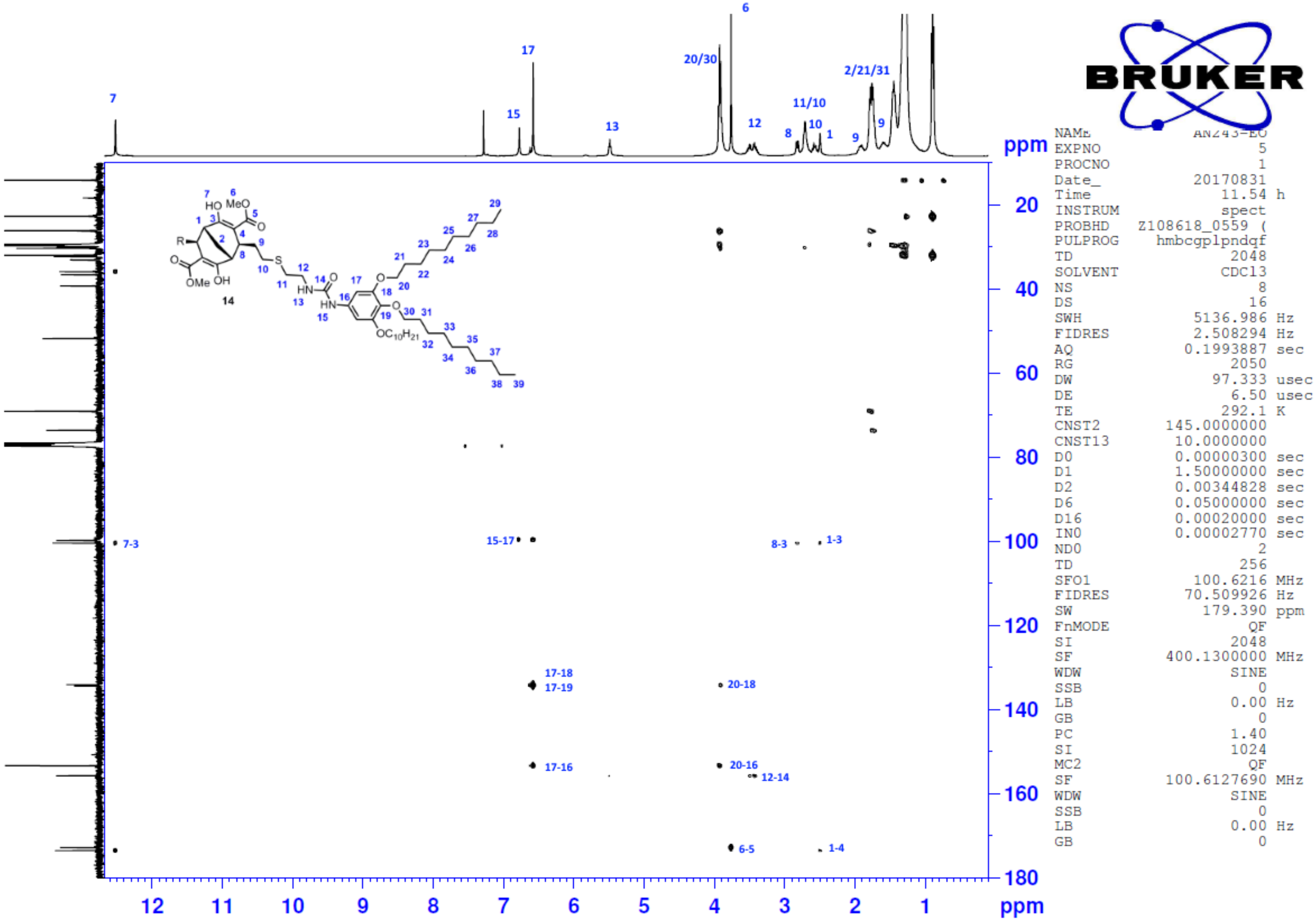

Figure S41: HMBC NMR spectrum of **14**.

return to Synthesis

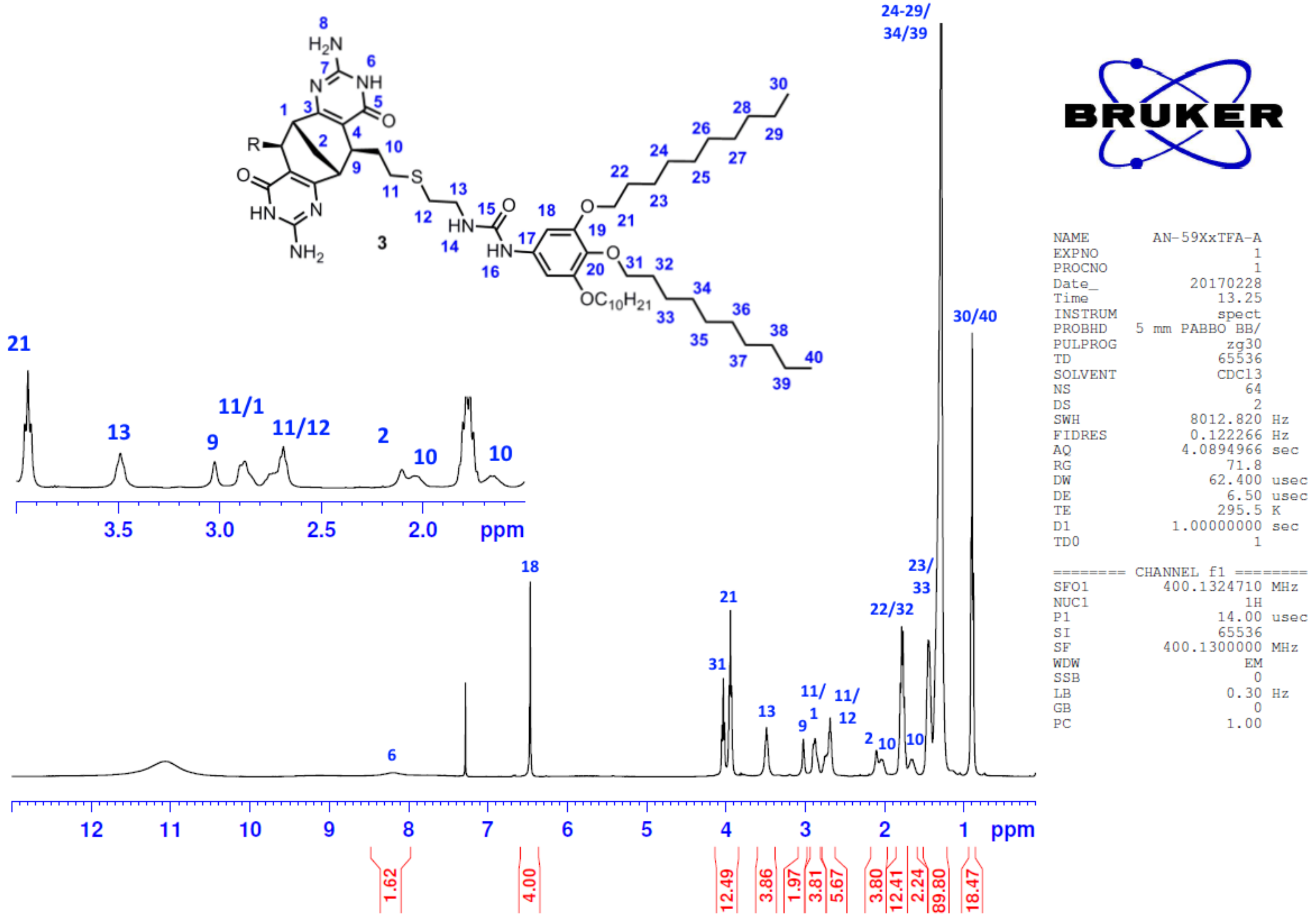

Figure S42: ^{1}H NMR spectrum of **3**.

return to Synthesis

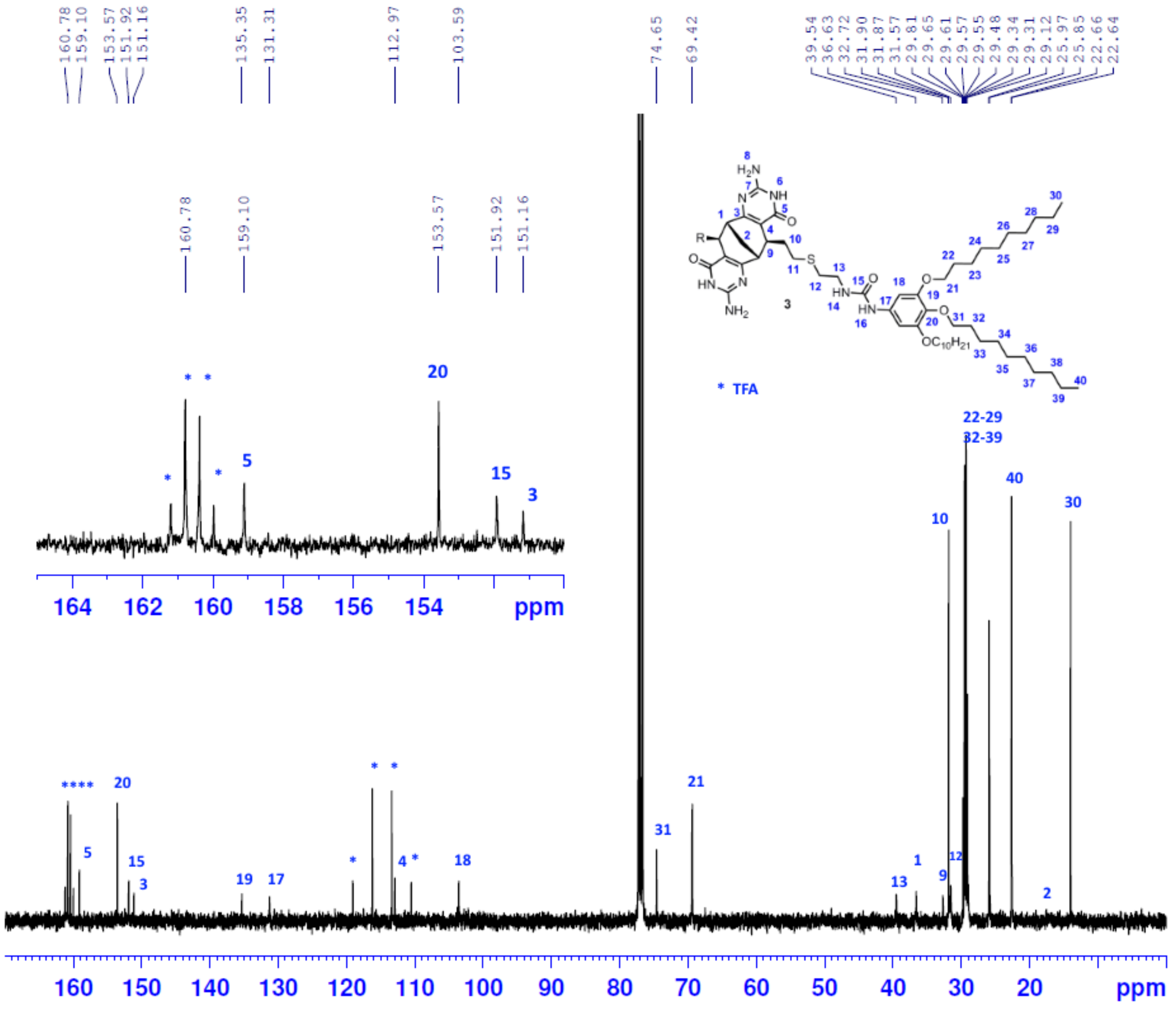

NAME AN-59XxTFA-A
EXPNO 3
PROCNO 1
Date_ 20170228
Time 14.12
INSTRUM spect
PROBHD 5 mm PABBO BB/
PULPROG zgpg30
TD 65536
SOLVENT CDCl3
NS 659
DS 4
SWH 24038.461 Hz
FIDRES 0.366798 Hz
AQ 1.3631988 sec
RG 90.5
DW 20.800 usec
DE 6.50 usec
TE 297.0 K
D1 2.00000000 sec
D11 0.03000000 sec
TD0 1

======== CHANNEL f1 ========
SFO1 100.6228293 MHz
NUC1 13C
P1 9.80 usec
SI 32768
SF 100.6127690 MHz
WDW EM
SSB 0
LB 1.00 Hz
GB 0
PC 1.40

Figure S43: ^{13}C NMR spectrum of **3**.

return to Synthesis

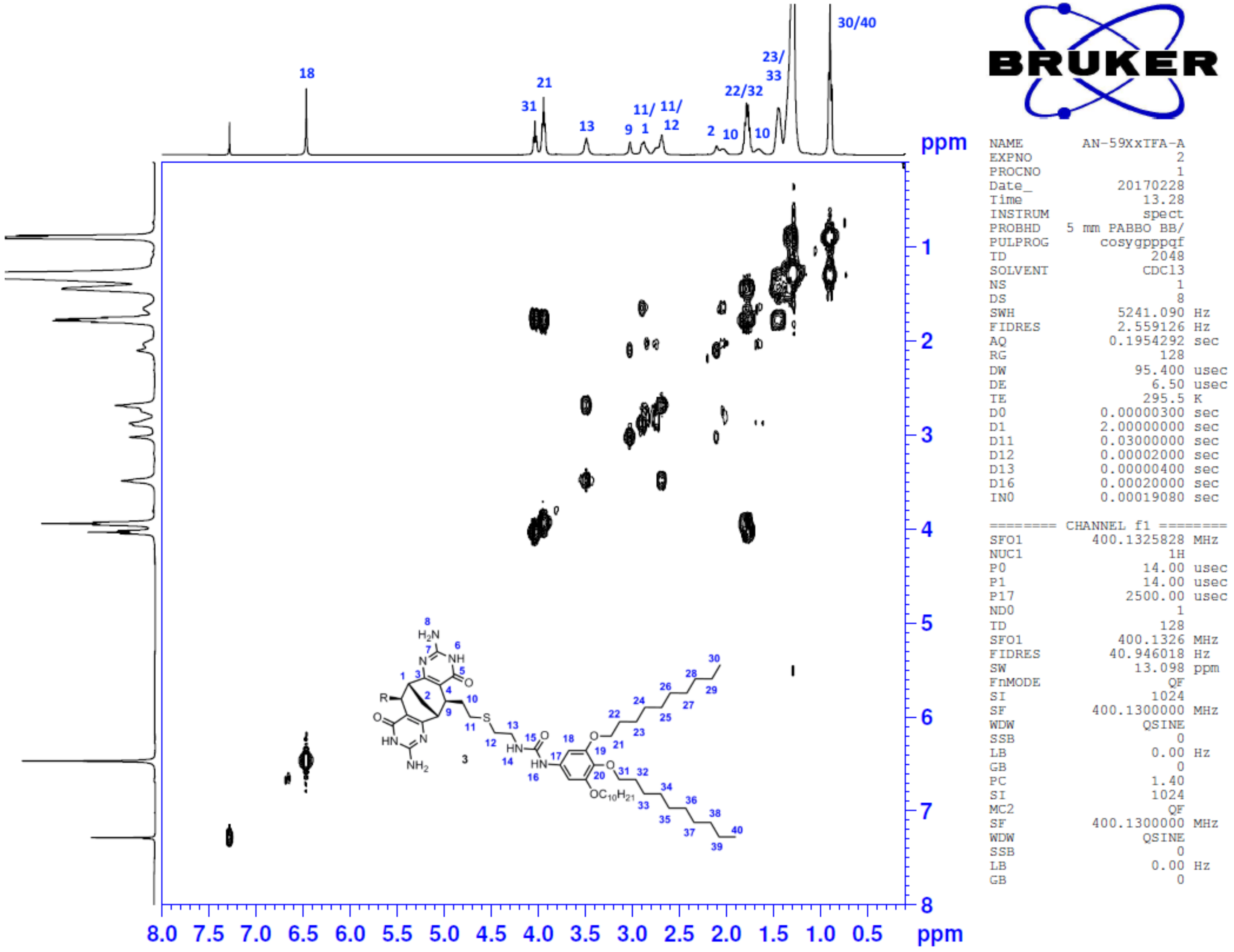

Figure S44: COSY NMR spectrum of **3**.

return to Synthesis

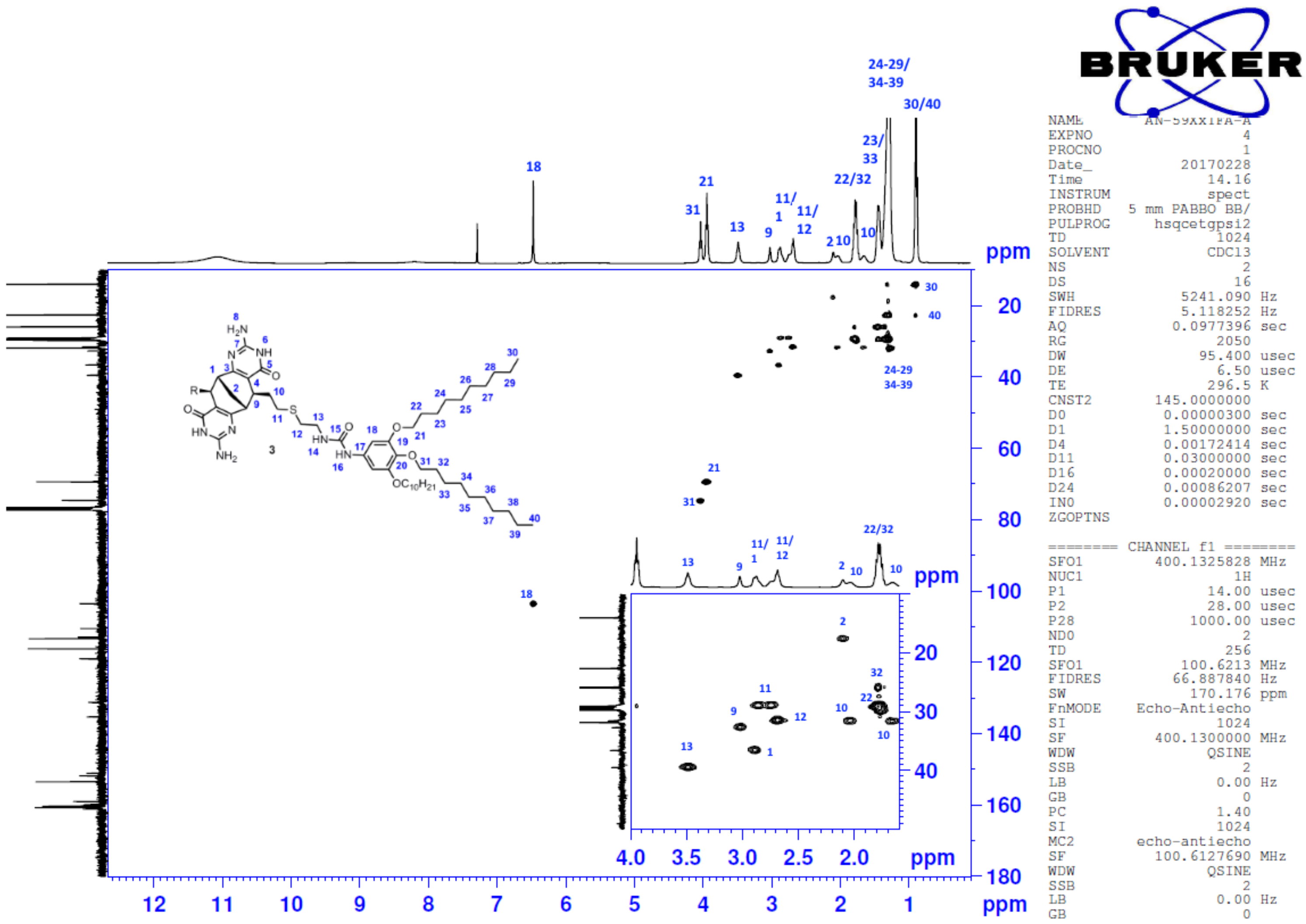

Figure S45: HSQC NMR spectrum of **3**.

return to Synthesis

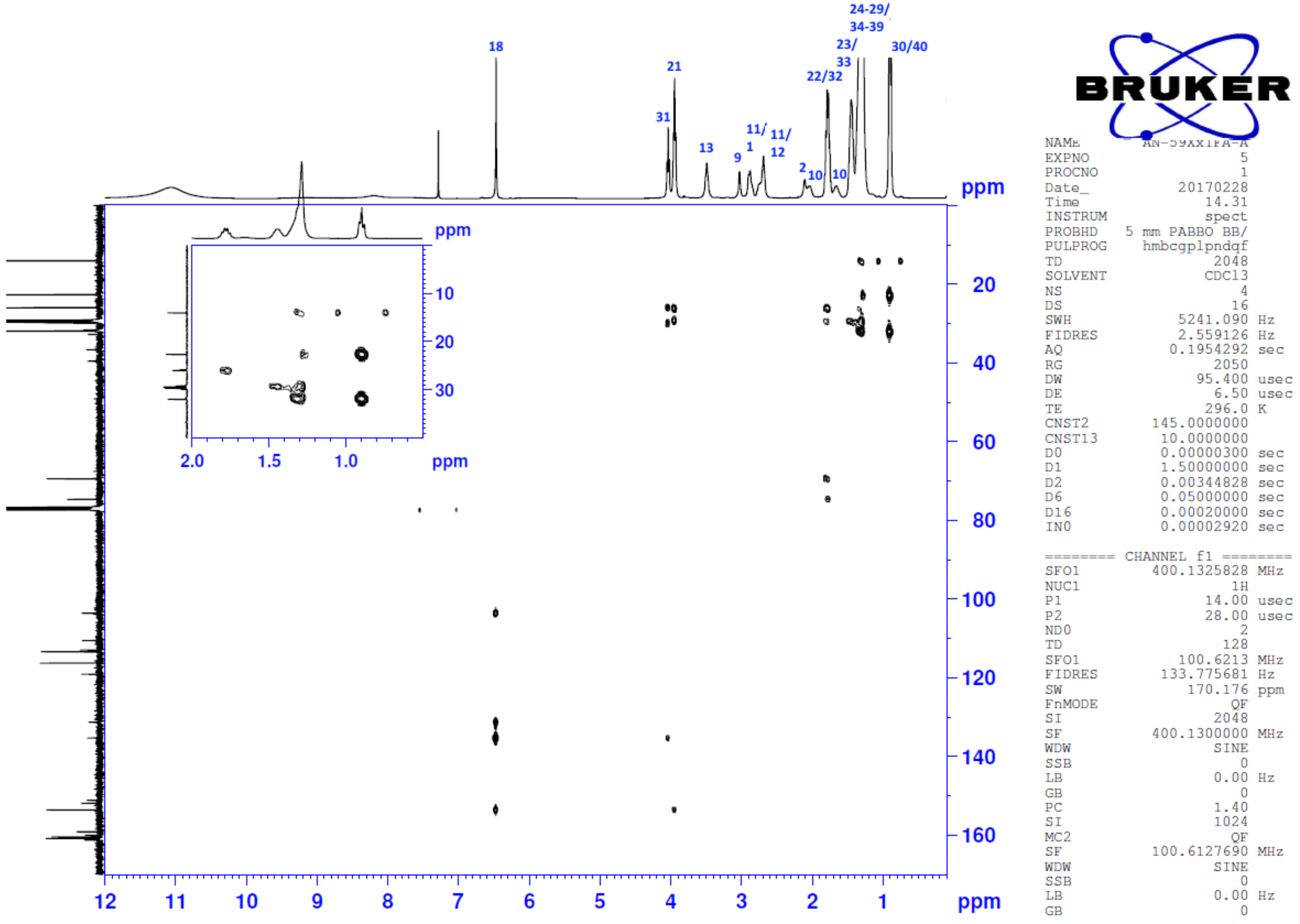

Figure S46: HMBC NMR spectrum of **3**.

return to Synthesis

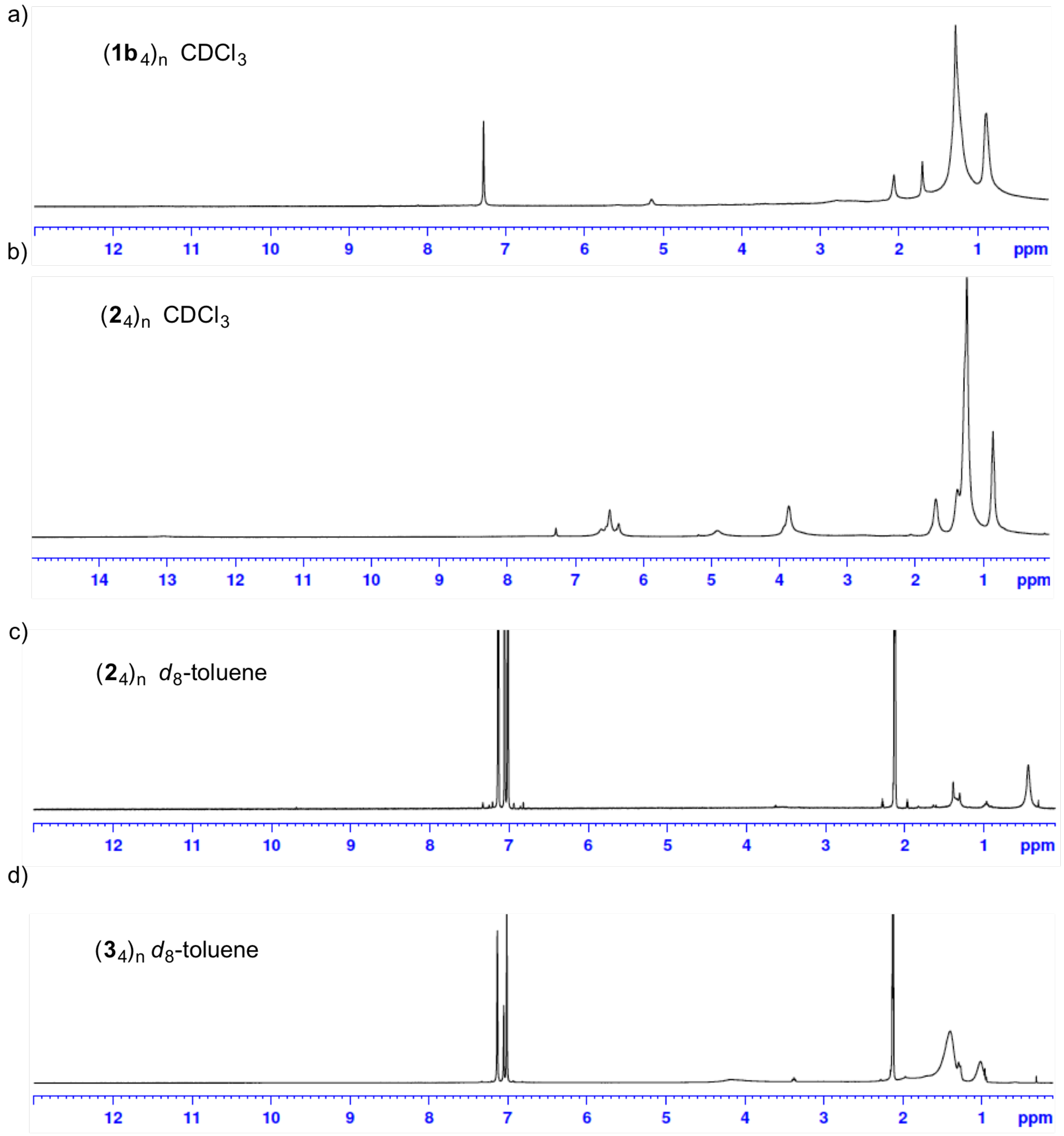

Figure S47: ^{1}H NMR spectra of **1-3** in polymeric state

S9. References

[1] E. Orentas, C.-J. Wallentin, K.-E. Bergquist, M. Lund, E. Butkus, K. Wärnmark, *Angew. Chem. Int. Ed.* **2011**, *50*, 2071–2074.

[2] D. Rackauskaite, R. Gegevicius, Y. Matsuo, K. Wärnmark, E. Orentas, *Angew. Chem. Int. Ed.* **2016**, *55*, 208–212.

[3] S. Hayashi, K. Hayamizu, *Bull. Chem. Soc. Jpn.* **1991**, *64*, 685–687.

[4] C. R. Morcombe, K. W. Zilm, *J. Magn. Reson.* **2003**, *162*, 479–486.

[5] S. Hayashi, K. Hayamizu, *Bull. Chem. Soc. Jpn* **1991**, *64*, 688–690.

[6] R. K. Harris, E. D. Becker, S. M. C. De Menezes, P. Granger, R. E. Hoffman, K. W. Zilm, et al., *Solid State Nucl. Magn. Reson.* **2008**, *33*, 41–56.

[7] G. E. Martin, C. E. Hadden, *J. Nat. Prod.* **2000**, *63*, 543–585.

[8] S. P. Brown, *Solid State Nucl. Magn. Reson.* **2012**, *41*, 1–27.

[9] S. P. Brown in *Modern Methods in Solid-state NMR, The Royal Society of Chemistry,Cambridge, Ed. P. Hodgkinson*, **2018**, pp. 39–74.

[10] W Sommer, J Gottwald, D. Demco, H. W. Spiess, *J. Magn. Reson. Series A* **1995**, *113*, 131–134.

[11] I. Schnell, A. Lupulescu, S. Hafner, D. E. Demco, H. W. Spiess, *J. Magn. Reson.* **1998**, *133*, 61–69.

[12] Z. Gan, J. P. Amoureux, J. Trébosc, *Chem. Phys. Lett.* **2007**, *435*, 163–169.

[13] A. S. Tatton, J. P. Bradley, D. Iuga, S. P. Brown, *Z. Phys. Chem.* **2012**, *226*, 1187–1204.

[14] T. Oas, R. Griffin, M. Levitt, *J. Chem. Phys.* **1988**, *89*, 692–695.

[15] G. Metz, X. Wu, S. O. Smith, *J. Magn. Reson. Series A* **1994**, *110*, 219–227.

[16] B. Fung, A. Khitrin, K. Ermolaev, *J. Magn. Reson.* **2000**, *142*, 97–101.

[17] N. Dalal, B. Triggs in Computer Vision and Pattern Recognition, 2005. CVPR 2005. IEEE Computer Society Conference on, *Vol. 1*, IEEE, **2005**, 886–893.
We thank Mindaugas Morkunas and Povilas Treigys for suggesting the HOG method for the estimation of the preferential orientation angles.

[18] Spartan'10, *Wavefunction Inc. 18401 Von Karman Avenue Suite 370. Irvine CA 92612 U.S.A.*
